# Supplementary material for: Uptake and effectiveness of a tailor-made online lifestyle programme targeting modifiable risk factors for dementia among middle-aged descendants of people with recently diagnosed dementia: study protocol of a cluster randomised controlled trial (Demin study)
Source: BMJ Open. 2020 Oct 16;10(10):e039439. doi: 10.1136/bmjopen-2020-039439 (PMC7569992; doi:10.1136/bmjopen-2020-039439)
Supplement: Supplementary data [file bmjopen-2020-039439supp001.pdf]

Supplementary file 1: Measures Dementia Risk Assessment

Through the online questionnaire and physical examination, data on thirteen currently known protective (i.e. Mediterranean diet, low/moderate alcohol consumption, cognitive activity) and risk factors (i.e. physical inactivity, smoking, loneliness, cardiovascular diseases, hypertension, high cholesterol, diabetes mellitus, obesity, renal dysfunction, depression) for dementia are collected (6,14,41). The measurements of these risk and protective factors are described below.

Protective factors

Mediterranean diet

The Mediterranean-DASH diet intervention for neurodegenerative delay (MIND) has shown to slow down cognitive decline (1) and to decrease the risk of developing AD (2). Therefore, adherence to the MIND-diet is determined with a number of items of the Food Frequency Questionnaire (FFQ), which is a reliable and valid instrument to measure intake of a specified list of food items in the general populations (3,4). The following healthy food groups of the MIND-diet were included in the questionnaire, such as vegetables (especially green leafy vegetables), nuts, berries, beans, whole grains, seafood, poultry, olive oil (1,2). Also five unhealthy food groups of the MIND-diet including red meat, butter, cheese, sweets and fried/fast food were asked (1,2). Based on the intake of the food groups, adherence to the MIND-diet is determined (0-14). A score of 14 represent good adherence to the MIND-diet *(See **Table 1** for the MIND-diet scoring table).*

Table 1. MIND-diet scoring table (2)

MIND components	Recommended quantity	Max score
Whole grains	≥ 3 serving spoons / day	1
Green leafy	≥ 6 serving spoons / week	1
Other vegetables	≥ 1 serving spoon / day	1
Berries (including other fruits)	≥ 2 portions / week *	1
Red Meats and products	< 4 portions / week	1

Fish	≥ 1 portion / week	1
Poultry	≥ 2 portions / week	1
Beans	> 3 serving spoons /week	1
Nuts	≥ 5 portions /week *	1
Fast/ fried food	< 1time / week	1
Butter, margarine	< 1 teaspoon/ day	1
Cheese	< 1 slice / week	1
Pastries, sweets	< 5 portions / week	1
Olive Oil (used as primary oil)	yes	1
**Total score**		**14**

** One portion is a handful of the given component*

Low/moderate alcohol consumption

Alcohol consumption was measured using the FFQ (5), including questions regarding the frequency of alcohol use (e.g. no consumption last month, 1 day per month, 2-3 days per month, 1 day per week, 2-3 days per week, 4-5 days per week, 6-7 days per week) and the average number of glasses of alcohol per day (range from zero to more than twelve) was asked. Subsequently, the average number of glasses per month was calculated in order to classify participants into: (i) non-alcohol consumers, (ii) low/moderate alcohol consumers or (iii) excessive alcohol consumers (6). Participants adhere to the national recommendations for no to low/moderate alcohol consumption, if participants drink one glass or less alcohol per day on average, without binge drinking (more than three glasses alcohol on one day for females and more than four glasses alcohol on one day for males)) (7).

High cognitive activity

Cognitive activity is assessed with the leisure time section of the Cognitive Reserve Index questionnaire (CRIq) (22). CRIq aims to measure cognitive reserve (CR), which is based on education, working activity and leisure time activity. For this study we are interested in the current cognitive

activities of the participants. Therefore, cognitive activity is determined by measuring working activity and leisure time activity. The frequency of eighteen leisure time activity is asked (e.g. (i) never, (ii) less than once a month, (iii) once a month, (iv) once every 2 weeks, (v) several times a week). Subsequently, a leisure time cognitive activity score is calculated, ranging from to 18 to 108, where a score of 50 or higher represent high cognitive activity (based on results of a survey on the knowledge, beliefs and attitudes towards dementia risk reduction among the general population of Groningen, see Table 2 and 3).

Additionally, participants are asked if they have a paid job and if so how many hours they spend on their job per week. High cognitive activity is defined as (i) working at least 24 hours per week or (ii) a leisure time cognitive activity score of at least 50.

Table 2. Cognitive activity (leisure time) scores stratified for education level and having a paid job in a survey conducted among the general population in Groningen

**Education level**	**Work**	**Leisure time score**	
		mean(SD)	(min-max)
Low (n=105)	no work (n=75)	39.57 (11.16)	13 – 63
	work (n=30)	41.73 (11.12)	25 – 68
Middle (n=154)	no work (n=72)	47.03 (9.95)	26 – 76
	work (n=82)	45.20 (9.49)	25 – 64
High (n=390)	no work (n= 135)	51.93 (10.19)	18 – 75
	work (n=255)	48.32 (8.97)	23 – 74

Table 3. Cognitive activity (leisure time) scores stratified for education level and having a paid job in a survey conducted among the general population in Groningen (subgroup: 40 – 60 year old)

**Education level**	**Work**	**Leisure time score**	
		mean(SD)	(min-max)

Low (n=29)	no work (n=7)	39.71 (9.67)	21 – 49
	work (n=22)	40.50 (10.52)	25 – 58
Middle (n=68)	no work (n=9)	43. 89 (13.15)	26 – 66
	work (n=59)	46.34 (8.87)	25 – 64
High (n=140)	no work (n= 16)	50.56 (10.59)	37 – 69
	work (n=124)	49.91 (9.24)	23 – 74

Risk factors

Physical inactivity

Physical activity levels are determined using the Short Questionnaire to Asses Health enhancing physical activity (SQUASH), a self-reported questionnaire and commonly used instrument in the Netherlands to assess physical activity (8). The SQUASH questionnaire has shown to be valid and reliable in measuring physical activity among the Dutch population (9–12). The SQUASH questionnaire includes questions on multiple activities referring to an average week in the last month, including actively commuting (walking, cycling) to (voluntary) work or school, physical activity at (voluntary) work or school, household activities and leisure time activities, including walking, cycling, gardening and sports. Participants were asked to fill in how many days a week they engaged in the activities (frequency), the average time per day spent on each activity (hours and minutes; duration) and the intensity at which they did the activity (low, moderate, high) (8). A standardized methodology was followed to calculate physical activity levels. Briefly, results from the SQUASH questionnaire are automatically converted to minutes per week spent in light (LPA) and moderate to vigorous (MVPA) intensity activities based on Metabolic Equivalent Tasks (METs) derived from the Ainsworth's compendium of physical activity (13). Physical activity levels are divided into the following categories: 0 minutes MVPA per week, 0 to 149 minutes MVPA per week, 150 to 299 minutes MVPA per week and 300 minutes MVPA per week and more. Physical inactivity is defined as less than 150 minutes per week MVPA (14).

Additionally, the questionnaire contained information on sitting behaviour, which is divided into sitting during transportation, working hours, watching television or using the computer at home. Participants are asked to fill in the number of hours and minutes on an average day in the past seven days during the week and on an average day during the weekend. This is similar to the sitting measure of the International Physical Activity Questionnaire (IPAQ) which has shown to be valid and reliable (15). Sitting time was divided into the following 4 categories: (i) less than 4 hours a day, (ii) 4 to 8 hours a day, (iii) 8 to 11 hours a day and (iv) at least 11 hours a day or more (16). Prolonged sitting time was defined as sitting at least for 8 hours a day or more.

Participants are physically inactive if they (i) are sitting on average more than 8 hours a day, irrespective of the physical activity, or (ii) are sitting on average 4 hours or more a day and are less active than 150 minutes MVPA per week.

Smoking

Participants are asked two questions to measure smoking behaviour: (i) whether they have smoked in the past month, and (ii) whether they have smoked in the past, for at least one year (17). Smoking behaviour is categorized into non-smoker, past smoker and current smoker. Current smokers are defined as people who reported smoking in the past month. Past smokers reported smoking for at least one year, but did not smoke in the past month.

Loneliness

Loneliness is measured using De Jong Gierveld Loneliness Scale , which is a reliable and valid instrument to measure emotional, social and overall loneliness (18). Possible answers on this 6-item scale are: (i) yes!, (ii) yes, (iii) more or less, (iv), no, (v) no!. The overall loneliness score is calculated by counting the neutral and negative ("no!", "no", or "more or less") answers on items 4, 5 and 6 (social loneliness score) and by counting the positive ("more or less"", "yes" or "yes!") answers on items 1,2 and 3 (emotional loneliness score). Subsequently, the overall loneliness score is categorized into: (i) not lonely (0-1), (ii) moderate lonely (2-4), (iii) severe lonely (5-6). Loneliness is defined as an overall loneliness score of 2 or higher (18).

Cardiovascular diseases

Participants are asked whether they have suffered or still suffer from one of the following cardiovascular diseases: angina pectoris, myocardial infarction, transient ischemic attack (TIA), stroke or peripheral arterial diseases (yes/no). Presence of a cardiovascular disease is defined as having at least one of the above mentioned diseases.

Hypertension

Hypertension is determined based on the blood pressure measurement in which the systolic and diastolic blood pressure is measured both three times consecutively. The average of the second and the third measurement is used to determine the presence of hypertension. Hypertension is present: (i) if the systolic blood pressure is higher than 140 mmHg or diastolic blood pressure is higher than 90 mmHg (19), or (ii) if participants indicate that they receive medication (i.e. diuretics, beta blockers, ACE-inhibitors, angiotensin 2 antagonists and calcium antagonists) for their hypertension .

High cholesterol

High cholesterol is defined based on direct laboratory measurements using the fasting blood samples and self-reported questionnaires. High cholesterol is present if (i) the Low Density Lipoprotein (LDL) is higher than 2.5 mmol/l or (ii) the ratio of total cholesterol (TC) and High Density Lipoprotein (HDL) is higher than 5 mmol/l (19) or (iii) participants indicate that they receive medication (i.e. simvastinin, atorvastatin, rosuvastatin, pravastatin, ezetimib) to lower their cholesterol levels.

Diabetes Mellitus

The presence of diabetes mellitus (or impaired blood glucose levels) is based on direct laboratory measurements using the fasting blood samples and self-reported questionnaires. Diabetes Mellitus is defined as: (i) glucose (fasting capillary blood) of 7.0 mmol/l or higher, or (ii) glucose (fasting capillary blood) lower than 7.0 mmol/l accompanied by HbA1C levels higher than 53 mmol/mol (20).

HbA1C provides additional information on the average blood glucose levels during the previous month, while glucose may differ during the day (20).

Obesity

Body weight and body height are measured during physical examination in order to determine their Body Mass Index (BMI=kg/m^2)(21). Obesity is present if BMI is 30 kg/m^2 or higher (22).

Renal dysfunction

The presence of renal dysfunction is based on direct laboratory measurements (serum creatinine levels) using the fasting blood samples and self-reported questionnaires (23). Subsequently, the estimated glomerular filtration rate (eGFR) is calculated using the 2009 CKD-EPI creatinine equation (24,25) in order to determine participant's renal function (25). Renal dysfunction is present if (i) eGFR is lower than 60 ml/min/1.73 m^2 (26), or (ii) participants indicate that they receive medical treatment for the established renal dysfunction.

Depression

The level of depressive symptoms is measured using the Centre for Epidemiologic Studies Depression scale (CES-D). The CES-D consists of 20 items and is a reliable and valid tool to measure the current level of depressive symptoms in the general population (27). Answer options for each item are: rarely or none of the time (0), some or a little of the time (1), occasionally or a moderate amount of time (2), and most of all of the time (3). Total score varying from 0 to 60, indicates the level of depressive symptoms, a higher score reflects a higher level of depressive symptoms. Depression is defined as (i) having a score of 16 or higher (27), or (ii) participants indicate that they receive medical treatment for their depressive symptoms.

REFERENCES

1. Morris MC, Tangney CC, Wang Y, Sacks FM, Barnes LL, Bennett DA, et al. MIND diet slows cognitive decline with aging. Alzheimer's Dement. 2015 Sep;11(9):1015–22.
2. Morris MC, Tangney CC, Wang Y, Sacks FM, Bennett DA, Aggarwal NT. MIND diet associated with reduced incidence of Alzheimer's disease. Alzheimer's Dement. 2015 Sep;11(9):1007–14.
3. Molag ML, de Vries JHM, Duif N, Ocké MC, Dagnelie PC, Goldbohm RA, et al. Selecting informative food items for compiling food-frequency questionnaires: comparison of procedures. Br J Nutr. 2010 Aug 8;104(03):446–56.
4. Siebelink E, Geelen A, de Vries JHM. Self-reported energy intake by FFQ compared with actual energy intake to maintain body weight in 516 adults. Br J Nutr. 2011 Jul 22;106(02):274–81.
5. Molag ML, de Vries JHM, Duif N, Ocké MC, Dagnelie PC, Goldbohm RA, et al. Selecting informative food items for compiling food-frequency questionnaires: comparison of procedures. Br J Nutr. 2010 Aug;104(03):446–56.
6. Anstey KJ, Mack HA, Cherbuin N. Alcohol Consumption as a Risk Factor for Dementia and Cognitive Decline: Meta-Analysis of Prospective Studies. Am J Geriatr Psychiatry. 2009 Jul;17(7):542–55.
7. Kromhout D, Spaaij CJK, de Goede J, et al. The 2015 Dutch food-based dietary guidelines. Eur J Clin Nutr 2016; 70: 869-78.
8. Wendel-Vos G, Schuit A. SQUASH: Short Questionnaire to Assess Health enhancing physical activity. Bilthoven, Netherlands; 2004.
9. Wagenmakers R, van den Akker-Scheek I, Groothoff JW, Zijlstra W, Bulstra SK, Kootstra JW, Wendel-Vos GC, van Raaij JJ, Stevens M. Reliability and validity of the short questionnaire to assess health-enhancing physical activity (SQUASH) in patients after total hip arthroplasty. BMC Musculoskelet Disord. 2008 Oct 17;9:141.
10. de Hollander EL, Zwart L, de Vries SI, Wendel-Vos W. The SQUASH was a more valid tool than the OBiN for categorizing adults according to the Dutch physical activity and the combined guideline. J Clin Epidemiol. 2012 Jan;65(1):73–81.
11. Wendel-Vos G. Reproducibility and relative validity of the short questionnaire to assess health-enhancing physical activity. J Clin Epidemiol. 2003 Dec;56(12):1163–9.
12. Nicolaou M, Gademan MGJ, Snijder MB, Engelbert RHH, Dijkshoorn H, Terwee CB, et al. Validation of the SQUASH Physical Activity Questionnaire in a Multi-Ethnic Population: The HELIUS Study. Buchowski M, editor. PLoS One. 2016 Aug;11(8):e0161066.
13. Ainsworth BE, Haskell WL, Herrmann SD, Meckes N, Bassett DR, Tudor-Locke C, et al. 2011 Compendium of Physical Activities: a second update of codes and MET values. Med Sci Sports Exerc. 2011 Aug;43(8):1575–81.
14. Weggemans RM, Backx FJG, Borghouts L, et al. The 2017 Dutch Physical Activity Guidelines. *Int J Behav Nutr Phys Act*. 2018;15(1):58. doi:10.1186/s12966-018-0661-9
15. Craig CL, Marshall AL, Sjostrom M, Bauman AE, Booth ML, Ainsworth BE, et al. International Physical Activity Questionnaire: 12-Country Reliability and Validity. Med Sci Sport Exerc. 2003 Aug;35(8):1381–95.
16. van der Ploeg HP, Chey T, Korda RJ, Banks E, Bauman A. Sitting Time and All-Cause Mortality Risk in 222 497 Australian Adults. Arch Intern Med. 2012 Mar 26;172(6):494.
17. World Health Organization. Global Adult Tobacco Survey Collaborative Group. Tobacco Questions for Surveys: A Subset of Key Questions from the Global Adult Tobacco Survey (GATS) 2 nd Edition. 2011;6–9.
18. Gierveld JDJ, Tilburg T Van. A 6-Item Scale for Overall, Emotional, and Social Loneliness. Res Aging. 2006 Sep;28(5):582–98.
19. Nederlandse Huisartsen Genootschap. Praktische handleiding bij de NHG-Standaard CVRM (2019). 2019.
20. Rutten G, De Grauw W, Nijpels G, Houweling S, Van de Laar F, Bilo H, et al. NHG-Standaard Diabetes mellitus type 2. Huisarts Wet. 2013; 56:512–25.
21. World Health Organization (WHO). Body mass index - BMI [Internet]. World Health Organization; [cited 2019 May 6]. Available from: http://www.euro.who.int/en/health-

topics/disease-prevention/nutrition/a-healthy-lifestyle/body-mass-index-bmi
22. Van Binsbergen J, Langens F, Dapper A, Van Halteren M, Glijsteen R, Cleyndert G, et al. NHG-Standaard Obesitas. Huisarts Wet. 2010; 53(11):609-25.
23. Perrone RD, Madias NE, Levey AS. Serum creatinine as an index of renal function: new insights into old concepts. Clin Chem. 1992;38(10).
24. Levey AS, Stevens LA, Schmid CH, Zhang YL, Castro AF, Feldman HI, et al. A new equation to estimate glomerular filtration rate. Ann Intern Med. 2009;150(9):604–12.
25. Levey AS, Inker LA, Coresh J. GFR estimation: From physiology to public health. Am J Kidney Dis. 2014;63(5):820–34.
26. De Grauw W, De Leest K, Schenk P, Scherpbier-De Haan N, Tjin-A-Ton J, Tuut M, et al. NHG-Standaard Chronische Nierschade. TPO - Prakt. 2018;13(5):26–9.
27. Smarr KL, Keefer AL. Measures of depression and depressive symptoms: Beck Depression Inventory-II (BDI-II), Center for Epidemiologic Studies Depression Scale (CES-D), Geriatric Depression Scale (GDS), Hospital Anxiety and Depression Scale (HADS), and Patient Health Questionna. Arthritis Care Res (Hoboken). 2011 Nov;63(S11):S454–66.
