# Supplementary material for: Uptake and effectiveness of a tailor-made online lifestyle programme targeting modifiable risk factors for dementia among middle-aged descendants of people with recently diagnosed dementia: study protocol of a cluster randomised controlled trial (Demin study)
Source: BMJ Open. 2020 Oct 16;10(10):e039439. doi: 10.1136/bmjopen-2020-039439 (PMC7569992; doi:10.1136/bmjopen-2020-039439)
Supplement: Supplementary data [file bmjopen-2020-039439supp002.pdf]

Supplementary file 2. Overview of assessment measures at baseline and follow up

**Table 1.** Assessment measures at baseline and follow up					
	**Baseline**	**3 months**	**6 months**	**9 months**	**12 months**
**GENERAL INFORMATION**					
Age, gender, ethnicity, education and postal code	Q				
Participation in the Lifelines cohort	Q				
Medical family history	Q				
Health literacy (S-TOFHLA, 3-items)	Q				
**RISK AND PROTECTIVE FACTORS**					
Smoking	Q	Q	Q	Q	Q
Physical inactivity (SQUASH, IPAQ sitting measure)	Q	Q	Q	Q	Q
Mediterranean diet (FFQ)	Q	Q	Q	Q	Q
Alcohol consumption (FFQ)	Q	Q	Q	Q	Q
High cognitive activity (CRIq adapted)	Q	Q	Q	Q	Q
Loneliness (de Jong Gierveld, 6-items)	Q	Q	Q	Q	Q
Cardiovascular diseases (CVD)	Q	Q	Q	Q	Q
Obesity (body weight, height)	Q+PE	Q	Q	Q	Q+PE
Hypertension (SBD, DBP)	Q+PE	Q	Q	Q	Q+PE
High cholesterol (LDL, HDL, TC)	Q+BS	Q	Q	Q	Q+BS
Diabetes Mellitus (glucose,	Q+BS	Q	Q	Q	Q+BS

HbA1C)					
Renal dysfunction (eGFR)	Q+BS	Q	Q	Q	Q+BS
Depression (CES-D)	Q	Q	Q	Q	Q
**OTHER PARAMETERS**					
Medical treatment of disease	Q	Q	Q	Q	Q
Motivation to change lifestyle (MCLHB-DRR)	Q	Q	Q	Q	Q
Stages of change	Q	Q	Q	Q	Q
Hearing problems	Q	Q	Q	Q	Q
Subjective stress (LDI)	Q				Q
Memory complaints	Q				
Quality of life (2 items of SF36, VAS-score)	Q				Q
Perceived living environment	Q				Q
Compliance lifestyle advice per individual health behaviour		Q	Q	Q	Q
Compliance advice contact with GP		Q	Q	Q	Q

SQUASH Short Questionnaire to Assess Health-enhancing physical activity, *IPAQ* International Physical Activity Questionnaire, *FFQ* Food Frequency Questionnaire, *CRIq* Cognitive Reserve Index questionnaire, *CVD* Cardiovascular diseases, *SBP* Systolic Blood Pressure, *DBP* Diastolic Blood Pressure, *HDL* high-density lipoproteins, *LDL* low-density lipoproteins, *TC* total cholesterol, *HbA1C* Hemoglobin A1C, *eGFR* estimated Glomerular Filtration Rate, *CES-D* Centre for Epidemiological Studies Depression Scale, *MCLHB-DRR* Motivation to Change Lifestyle and Health Behavior for Dementia Risk Reduction Scale, LDI Long-term Difficulties Inventory, *SF36* Short Form 36 items, *VAS* Visual Analogue Scale

Q: Online questionnaire; PE: Physical examination; BS: Blood sample
