# Supplementary material for: Uptake and effectiveness of a tailor-made online lifestyle programme targeting modifiable risk factors for dementia among middle-aged descendants of people with recently diagnosed dementia: study protocol of a cluster randomised controlled trial (Demin study)
Source: BMJ Open. 2020 Oct 16;10(10):e039439. doi: 10.1136/bmjopen-2020-039439 (PMC7569992; doi:10.1136/bmjopen-2020-039439)
Supplement: Supplementary data [file bmjopen-2020-039439supp003.pdf]

Voornaam Achternaam
Straatnaam 12
1234AB Plaatsnaam
0612345678
demin@umcg.nl
ABCDE
Aangemeld op: 01-02-2020 10:01:16

01-02-2020 10:06:24

Beste Voornaam Achternaam,

Indien u wilt deelnemen aan dit onderzoek, vragen wij u dit toestemmingformulier door middel van een elektronische handtekening te ondertekenen. Hieronder vindt u de voorwaarde voor deelname aan dit onderzoek.

Wanneer u het toestemmingsformulier ondertekent verklaart u dat:
- u de informatiefolder met bijlagen en bovenstaande informatie heeft gelezen en hiermee voldoende bent geïnformeerd over het doel en de uitvoering van het onderzoek.
- U de mogelijkheid heeft gehad om aanvullende vragen te stellen (telefonisch of per mail), welke naar tevredenheid zijn beantwoord.
- u genoeg tijd had om te beslissen of u wilt deelnemen.
- u weet dat deelname vrijwillig is en dat u op ieder moment kan beslissen om toch niet mee te doen of te stoppen met het onderzoek.
- u weet dat u op de hoogte gesteld kan worden van medische relevante bevindingen.

U geeft toestemming:

– voor deelname aan het landelijk proef-bevolkingsonderzoek naar de beschermende en risicofactoren voor dementie (Demin studie).
– dat u in de toekomst opnieuw benaderd kan worden voor deelname aan aanvullend onderzoek.
– om uw onderzoeksgegevens te koppelen aan gegevens van het Centraal Bureau voor Statistiek (CBS), zoals uw gegevens over woonomgeving (bijvoorbeeld sportfaciliteiten).

Met vriendelijke groet,

Het Demin team
www.demin.nl

Ik verklaar dat ik deze proefpersoon volledig heb geïnformeerd over het genoemde onderzoek.

Als er tijdens het onderzoek informatie bekend wordt die de toestemming van de proefpersoon zou kunnen beïnvloeden, dan breng ik hem/haar daarvan tijdig op de hoogte.

Naam hoofdonderzoeker:
Handtekening: Datum:
Tijdstip:

De deelnemer krijgt een volledige informatiebrief, samen met een kopie van het getekende toestemmingsformulier.
