# Supplementary material for: Uptake and effectiveness of a tailor-made online lifestyle programme targeting modifiable risk factors for dementia among middle-aged descendants of people with recently diagnosed dementia: study protocol of a cluster randomised controlled trial (Demin study)
Source: BMJ Open. 2020 Oct 16;10(10):e039439. doi: 10.1136/bmjopen-2020-039439 (PMC7569992; doi:10.1136/bmjopen-2020-039439)
Supplement: Author's manuscript [file bmjopen-2020-039439.draft_revisions.pdf]

BMJ Open is committed to open peer review. As part of this commitment we make the peer review history of every article we publish publicly available.

When an article is published we post the peer reviewers' comments and the authors' responses online. We also post the versions of the paper that were used during peer review. These are the versions that the peer review comments apply to.

The versions of the paper that follow are the versions that were submitted during the peer review process. They are not the versions of record or the final published versions. They should not be cited or distributed as the published version of this manuscript.

BMJ Open is an open access journal and the full, final, typeset and author-corrected version of record of the manuscript is available on our site with no access controls, subscription charges or pay-per-view fees (http://bmjopen.bmj.com).

If you have any questions on BMJ Open's open peer review process please email info.bmjopen@bmj.com

BMJ Open

# The uptake and effectiveness of a tailor-made online lifestyle program targeting modifiable risk factors for dementia among middle-aged descendants of people with recently diagnosed dementia: study protocol of a cluster randomised controlled trial (Demin study)



| | |
|---|---|
| Journal: | *BMJ Open* |
| Manuscript ID | bmjopen-2020-039439 |
| Article Type: | Protocol |
| Date Submitted by the Author: | 15-Apr-2020 |



| | |
|---|---|
| Complete List of Authors: | Vrijsen, Joyce; University of Groningen, University Medical Centre Groningen, Epidemiology<br>Abu-Hanna, Ameen; University of Amsterdam, Amsterdam UMC, Medical Informatics<br>Maeckelberghe, Els; University of Groningen, University Medical Centre Groningen, Wenckebach Institute for Training and Education<br>De Deyn, Peter Paul; University of Groningen, University Medical Centre Groningen, Neurology and Alzheimer Centre Groningen<br>de Winter, Andrea; University of Groningen, University Medical Centre Groningen, Health Sciences<br>Reesink, Fransje; University of Groningen, University Medical Centre Groningen, Neurology and Alzheimer Centre Groningen<br>Oude Voshaar, Richard; University of Groningen, University Medical Center Groningen, Psychiatry<br>Buskens, Erik; University of Groningen, University Medical Centre Groningen, Epidemiology<br>de Rooij, Sophia; Medical Spectrum Twente, Medical School<br>Smidt, Nynke; University of Groningen, University Medical Centre Groningen, Epidemiology |



| | |
|---|---|
| Keywords: | Dementia < NEUROLOGY, Risk management < HEALTH SERVICES ADMINISTRATION & MANAGEMENT, EPIDEMIOLOGY, PREVENTIVE MEDICINE, PUBLIC HEALTH |


SCHOLARONE™ Manuscripts

*I, the Submitting Author has the right to grant and does grant on behalf of all authors of the Work (as defined in the below author licence), an exclusive licence and/or a non-exclusive licence for contributions from authors who are: i) UK Crown employees; ii) where BMJ has agreed a CC-BY licence shall apply, and/or iii) in accordance with the terms applicable for US Federal Government officers or employees acting as part of their official duties; on a worldwide, perpetual, irrevocable, royalty-free basis to BMJ Publishing Group Ltd ("BMJ") its licensees and where the relevant Journal is co-owned by BMJ to the co-owners of the Journal, to publish the Work in this journal and any other BMJ products and to exploit all rights, as set out in our licence.*

*The Submitting Author accepts and understands that any supply made under these terms is made by BMJ to the Submitting Author unless you are acting as an employee on behalf of your employer or a postgraduate student of an affiliated institution which is paying any applicable article publishing charge ("APC") for Open Access articles. Where the Submitting Author wishes to make the Work available on an Open Access basis (and intends to pay the relevant APC), the terms of reuse of such Open Access shall be governed by a Creative Commons licence – details of these licences and which Creative Commons licence will apply to this Work are set out in our licence referred to above.*

*Other than as permitted in any relevant BMJ Author's Self Archiving Policies, I confirm this Work has not been accepted for publication elsewhere, is not being considered for publication elsewhere and does not duplicate material already published. I confirm all authors consent to publication of this Work and authorise the granting of this licence.*

For peer review only

**TITLE (WORD COUNT: 3998)**

The uptake and effectiveness of a tailor-made online lifestyle program targeting modifiable risk factors for dementia among middle-aged descendants of people with recently diagnosed dementia: study protocol of a cluster randomised controlled trial (Demin study)

**Issue date:** 13 April 2020

**AUTHORS**

Vrijsen J[1]*, Abu-Hanna A[2], Maeckelberghe ELM[3], De Deyn PP[4], de Winter AF[5], Reesink FE[4], Oude Voshaar RC[6], Buskens E[1], de Rooij SEJA[7], Smidt N[1], for the Demin consortium.

*Corresponding author. University Medical Centre Groningen, Department of Epidemiology, Hanzeplein 1, PO Box 30 001, FA40, 9700 RB Groningen, The Netherlands. E-mail: j.vrijsen@umcg.nl; Phone: +31625650782

**Author affiliations**

1 University of Groningen, University Medical Centre Groningen, Department of Epidemiology, Groningen, the Netherlands

2 University of Amsterdam, Amsterdam UMC, Department of Medical Informatics, Amsterdam, the Netherlands

3 University of Groningen, University Medical Centre Groningen, Wenckebach Institute for Training and Education, Groningen, the Netherlands

4 University of Groningen, University Medical Centre Groningen, Department of Neurology and Alzheimer Centre Groningen, Groningen, the Netherlands

5 University of Groningen, University Medical Centre Groningen, Department of Health Sciences, Groningen, the Netherlands

6 University of Groningen, University Medical Centre Groningen, Department of Psychiatry, Groningen, the Netherlands

7 Medical Spectrum Twente, Medical School, Enschede, the Netherlands



For peer review only - http://bmjopen.bmj.com/site/about/guidelines.xhtml

**Demin consortium (28)**

Abma EM, Abu-Hanna A, Buskens E, Claassen JAHR, De Deyn PP, Feitsma T , Hempenius L, Hoogmoed J, Kamper A, Koek HL, Kragt JJ, Lagro J, Lambooij SLE, Langedijk M, Maeckelberghe ELM, Mattace Raso FUS, Meinardi MC, Oude Voshaar RC, Reesink FE, de Rooij SEJA, Scheepmaker AJTM, Smidt N, Spies P, Taekema D, Verkuyl J, Vingerhoets R, Vrijsen J, de Winter AF.

For peer review only



For peer review only - http://bmjopen.bmj.com/site/about/guidelines.xhtml

## ABSTRACT

**Introduction** Descendants of dementia patients have a higher risk to develop dementia. This study aims to investigate the uptake and effectiveness of an online tailor-made lifestyle program for Dementia Risk Reduction (DRR) among middle-aged descendants of people with recently diagnosed late-onset dementia.

**Methods and analysis** Demin is a cluster randomised controlled trial, aiming to include 21 memory clinics of which thirteen will be randomly allocated to the passive (poster and flyer in waiting room) and eight to the active recruitment strategy (additional personal invitation by members of the team of the memory clinic). We aim to recruit 378 participants, aged 40-60 years, with a parent who is recently diagnosed with Alzheimer's Disease or Vascular Dementia at one of the participating memory clinics. All participants receive a dementia risk assessment (online questionnaire, physical examination and fasting blood sample) and subsequently an online tailor-made lifestyle advice regarding protective (Mediterranean diet, low/moderate alcohol consumption, high cognitive activity) and risk factors (physical inactivity, smoking, loneliness, cardiovascular disease, hypertension, high cholesterol, diabetes, obesity, renal dysfunction, depression) for dementia. The primary outcome is the difference in uptake between the two recruitment strategies. Secondary outcome measures are the change(s) in 1) the Lifestyle for Brain Health (LIBRA) score, 2) individual health behaviours, 3) health beliefs and attitudes towards DRR and 4) compliance to the tailor-made lifestyle advice and that of the general practitioner. Outcomes will be measures at 3, 6, 9 and 12 months after baseline assessment. The effectiveness of this online tailor-made lifestyle program will be evaluated by comparing Demin participants to a matched control group (Lifelines cohort).

**Ethics and dissemination** This study has been approved by the Dutch Ministry of Health, Welfare and Sport according to the Population Screening Act. All participants have to give online informed consent using SMS-tan.

**Trial registration number** NTR7434

For peer review only

## ARTICLE SUMMARY

### Strengths and limitations of this study

- This is the first multicentre trial that focuses on dementia risk reduction in middle-aged descendants of recently diagnosed patients with Alzheimer's disease or Vascular dementia.
- The program gives participants insight in their risk and protective factors for dementia and provides a tailor-made online lifestyle advice with regard to thirteen modifiable risk factors for dementia, taking the stages of (health behaviour) change into account.
- The application ensures the privacy of the participants by using SMS-tan for logging in their personal account and signing the electronic informed consent form.
- The web-based application (demin.nl) functions fully automatically, making it easy to implement the study in other memory clinics and settings.
- Changing health behaviour is difficult and it is unclear whether a tailor-made online lifestyle advice is sufficient to change health behaviour and to maintain a healthy lifestyle.

**KEY WORDS**

Dementia

Health behavior

Risk reduction behavior

Lifestyle

Middle aged



For peer review only - http://bmjopen.bmj.com/site/about/guidelines.xhtml

## INTRODUCTION

Dementia is considered a major public health concern [1]. Due to the ageing population the number of dementia cases will increase substantially in the next decades. In 2015, more than 46 million people worldwide were affected by dementia and this number is expected to increase to 131 million by 2050 [2]. This rise in people with dementia carries a high economic and social burden for society [1]. In 2015, global costs of dementia reached 818 billion US dollars and will increase further [3]. Currently, no curative treatments are available. Therefore, prevention is a key element to counteract the dementia epidemic [4,5].

The most common types of dementia are Alzheimer's disease (AD) (60-70%) ,Vascular dementia (VD) (15-20%) or a combination of AD and VD (mixed dementia) [6–8]. The presence of a first-degree relative with AD doubles the risk for developing AD [9]. This increased risk has several reasons. Firstly, descendants of people with AD more often have a higher genetic predisposition for AD (e.g. carrier of the Apo lipoprotein E (APOE) ε4 allele) [9]. Secondly, high blood pressure, vascular diseases and other vascular risk factors (i.e. diabetes type 2, obesity, hypercholesterolemia) often cluster in families [10]. Lastly, psychosocial behaviour runs in the family and also affects health behaviour and lifestyle [11,12]. Not surprisingly, individuals with a parent who is recently diagnosed with AD or VD often worry about their own risk of developing dementia. Therefore, this life event (parental diagnosis of dementia) might encourage the willingness of individuals to change their health behaviour [13].

Parental family history has been associated with an increased risk of dementia independently of known genetic risk factors [9,14]. Therefore, a healthy lifestyle might be beneficial for individuals with a positive family history. Over the last decade, evidence of modifiable risk factors for dementia has been mounting [4,6,15]. The Lancet commission on dementia prevention, intervention and care demonstrated that 35% of the dementia cases is attributable to modifiable risk factors (i.e. less education, hearing loss, midlife hypertension, midlife obesity, smoking, depression, physical inactivity, social isolation and diabetes) and recommended to start interventions including more



For peer review only - http://bmjopen.bmj.com/site/about/guidelines.xhtml

For peer review only

childhood education, promotion of physical exercise, reduction of smoking, maintaining social engagement and management of hypertension, diabetes, obesity, depression and hearing loss [4,6,16]. Other major risk factors are hyperlipidaemia, coronary heart disease, renal dysfunction, Mediterranean diet and cognitive activity [15].

Only few studies examined the effectiveness of targeting these modifiable factors on cognitive decline and dementia incidence through a multi-domain intervention, such as the (Finnish Geriatric Intervention Study to Prevent Cognitive Impairment and Disability) FINGER study [17], the (Prevention of Dementia by Intensive Vascular care) PreDIVA study [18] and the (The Multi-domain Alzheimer Preventive Trial) MAPT study [19]. These studies, with a follow-up varying from two to six years, found small or non-significant effects on cognition in older participants (e.g. >60 years) [17–19]. Starting multi-domain interventions earlier in life might be promising as cognitive decline begins already in midlife [20,21]. Furthermore, tailoring interventions improves the effectiveness of health behaviour change interventions [22]. Web-based interventions have the potential to support health behaviour change as there is the opportunity to tailor lifestyle advice [23–26]. They were especially effective when a theoretical basis or conceptual framework (e.g. Health belief model (HBM), Trans theoretical model (TTM), Theory of planned behaviour (TPB), I(integrated)-Change model [27–31]), behaviour change techniques (e.g. providing feedback on performance and information on the consequences of unhealthy behaviour) and several modes of delivery had been used [32]. Additionally, the effectiveness of web-based interventions is enhanced by using automated follow-up messages by email of text message (SMS) [22].

A challenge of health behaviour change interventions is to achieve a high level of uptake for screening (e.g. assessing risk and protective factors for dementia). A systematic review identified a large variation in uptake in health checks and lifestyle intervention programs [33], depending on the type of recruitment strategy. Uptake also depends on other factors as described in social cognition models (e.g. knowledge, perceived susceptibility and severity, facilitators, barriers and attitude towards such interventions) [27–31]. Therefore, information on dementia, the risk and protective factors for



For peer review only - http://bmjopen.bmj.com/site/about/guidelines.xhtml

dementia, heritability, and how to tackle risk and protective factors for dementia are important factors in the development of a web-based intervention.

To our knowledge, none of the health behaviour intervention studies were aimed at a specific group of middle-aged adults with increased risk for dementia due to their positive parental family history for dementia. Therefore, this study aims to investigate the uptake and effectiveness of a tailor-made online lifestyle program for dementia risk reduction among middle-aged descendants of recently diagnosed (in the last six months) people with AD or VD in the Netherlands. It is expected that middle-aged descendants of recently diagnosed people with AD or VD are more willing to assess their risk and motivated to adopt a healthier lifestyle as they just realized their (family) risk.

## METHODS AND ANALYSIS

### Study setting and design

This study is a pragmatic cluster randomized controlled trial (RCT), including 21 participating memory clinics in the Netherlands who are randomly allocated to a passive or active recruitment of participants. Memory clinics allocated to the active recruitment strategy invite potential participants face-to-face by a member of the team of the memory clinic to participate in the tailor-made online lifestyle program for dementia risk reduction (also called the Demin study), next to posters and flyers that are placed in the waiting room of the memory clinic. Memory clinics allocated to the passive recruitment strategy, do not invite potential participants pro-actively, but invite potential participants to participate in the Demin study by posters and flyers that are placed in the waiting room of the memory clinic.

Patients with AD or VD (or their caregivers) receive an envelope either at the registration desk of the memory clinic or after the consult of the patient (only with active recruitment). This envelope is addressed to the middle-aged descendants of patients with recently diagnosed AD or VD and includes a patient information form (PIF) with information about the content of the study, the advantages and disadvantages of study participation and how potential participants can participate. Potential



For peer review only

167 participants (one family member per patient) are asked to register themselves (e.g. making an account)
168 on the Demin website (www.demin.nl), by using the memory clinic specific login access code, which
169 is reported on the front page of the PIF and represents the memory clinic in which the parent was
170 diagnosed. The decision to participate is confirmed by the participants by signing the online informed
171 consent form (electronic signature by using SMS-tan). After signing this form, individuals from both
172 recruitment strategies are able to log in to their personalized website 'My Demin' and continue the
173 intervention in an equal manner. The personalized website 'My Demin' is secured and only accessible
174 for the participant by logging in with their personal e-mail address, password and SMS-tan code. 'My
175 Demin' contains the following information: 1) My personal (account) information, 2) Message inbox,
176 3) My online questionnaires, 4) My personal health profile including online tailor-made lifestyle
177 advice. After participants have completed the online questionnaire, they automatically receive a
178 message with a request to make an appointment for physical examination including a fasting blood
179 sample. Moreover, participants can invite siblings to participate in the study in 'My Demin'.
180 The functionalities provided by the Demin website are based on the literature and input we received
181 from people with a parent with dementia (focus group discussions).
182

183 **Randomization of memory clinics**

184 To prevent contamination between the two recruitment strategies, randomization is performed at the
185 level of the memory clinics. To enhance comparability between the intervention (participants of the
186 active recruitment strategy) and control group (participants of the passive recruitment strategy), the
187 memory clinics will be matched and randomised by a statistician, who is blind to the identity of the
188 memory clinics and not involved in the study. Firstly, all participating memory clinics will be matched
189 into pairs based on the following criteria: (i) number of newly diagnosed dementia (VD, AD or mixed
190 dementia) patients seen per year (range vary from 60 to 350 patients per year) and (ii) the average
191 social economic position (SEP) of the population living around the memory clinic (neighbourhood
192 SEP), based on data from Statistics Netherlands [34]. Secondly, the matched memory clinics will be
193 randomized (pairwise randomization) to an active recruitment strategy or passive recruitment strategy
194 using a computer-generated random number list. As we expect a higher response rate in the active



For peer review only - http://bmjopen.bmj.com/site/about/guidelines.xhtml

For peer review only

recruitment strategy group, we use an active : passive recruitment strategy ratio of 8:13 (see sample size calculations).

**Study population**

Eligible participants are middle-aged individuals (40-60 years old) with a parent who is recently (less than 6 months ago) diagnosed with AD or VD (or mixed dementia) at one of the participating memory clinics in the Netherlands (see acknowledgement). Individuals should provide informed consent, be able to fill out an online Dutch questionnaire. Pregnant women are excluded from participation.

**Sample size calculations**

The primary outcome measure is uptake, which is defined as the percentage of eligible individuals that signed the online informed consent form and completed baseline assessment (online questionnaire and physical examination and a fasting blood sample). In order to detect a difference of 20% in uptake between the passive and active recruitment strategy (30% versus 50%), we need 94 participants in each group to achieve a power of 80% with alpha levels of 0.05 (total = 188 participants). To take cluster randomization into account, we use the formula 1+ ((n-1)*ICC) (inflation factor), where n is the average number of included participants per memory clinic and the ICC the Intra Class Correlation [35]. The ICC is unknown, but an ICC of 0.05 is a common value for cluster randomized controlled trials in hospitals [36]. The estimated average of included participants per memory clinic per year is n=15 using a passive recruitment strategy and n=25 using an active recruitment strategy, taking into account non-response. With unequal cluster sizes, 'n' is replaced by 'm', where m is the sum of $(M)^2$/sum(M) $((15^2+25^2) / (15+25))$ [37]. This results in a sample size inflation factor of (1+ ((21.25-1)*0.05)= 2.01. Therefore, the total number of participants needed is 378 (2.01 * 188). In order to recruit 378 participants, we need 21 memory clinics, of which eight memory clinics (responsible for 189 included participants) will be allocated to the active recruitment strategy and thirteen memory clinics (responsible for 189 included participants) will be allocated to the passive recruitment strategy.

**Demin website**



For peer review only - http://bmjopen.bmj.com/site/about/guidelines.xhtml

The Demin website is available for everyone and provides information about dementia, heredity of dementia, risk and protective factors for dementia, and how to tackle potential risk factors for dementia. The health information will be provided by written text and in an audio-visual format, such as a spoken animation, to assure inclusion of participants with different levels of health literacy. [38].. According to the cognitive theory of multimedia learning (CTML), people process visual and auditory information through different channels [39,40]. It is known that health information provided by various channels, such as written text and spoken animations, improves information processing compared to information only provided through written text or spoken animations [39,40]. The instructions for registration (making an account, signing informed consent) are also provided as written text as visual screenshots representing the steps of the registration process.

**Online tailor-made lifestyle program for dementia risk reduction**

After participants give online informed consent, participants have access to the online tailor-made lifestyle program for dementia risk reduction, which consists of 1) a dementia risk assessment and 2) an online tailor-made lifestyle advice including a personal health profile targeting risk and protective factors for dementia.

**1. Dementia risk assessment**

The dementia risk assessment consists of filling out an online questionnaire (in 'My Demin') and physical examination, including a fasting blood sample, at one of the 21 participating memory clinics in order to determine whether risk and protective factors are present. In order to minimize the amount of missing data, validation and skip-and-fail rules were implemented in the online questionnaire. Furthermore, automatic reminders are sent to the participant if the online questionnaire was not filled in within two weeks. Physical examination will be conducted by the team of the local memory clinic and includes the following measurements: height (in cm) (SECA 222 stadiometer), body weight (in kg) without shoes (SECA 761 scale), waist- and hip circumference (in cm) (SECA 200 measuring tape), and three measurements of diastolic and systolic blood pressure (in mmHg) (Welch Allyn 'Spot Vital Signs' [41]). After physical examination, which takes approximately 15 minutes, a fasting blood



For peer review only - http://bmjopen.bmj.com/site/about/guidelines.xhtml

sample (maximum of 21 ml) is taken for direct laboratory measurement of glucose, HbA1C, total cholesterol, High-density-lipoprotein (HDL), Low-density-lipoprotein (LDL), triglycerides and serum creatinine. The results of the physical examination (height, body weight, blood pressure, waist- and hip circumference) are sent to the researcher (J. Vrijsen) to check the entry of the results by the participants. The results of the direct laboratory measurements are sent to the medical doctor (E.M. Abma) of the University Medical Centre Groningen to check for deviating values.

**Risk and protective factors for dementia**

Through the online questionnaire and physical examination, data on thirteen currently known protective (i.e. Mediterranean diet, low/moderate alcohol consumption, cognitive activity) and risk factors (i.e. physical inactivity, smoking, loneliness, cardiovascular diseases, hypertension, high cholesterol, diabetes mellitus, obesity, renal dysfunction, depression) for dementia are collected [6,15,42]. See **Table 1** for an overview of the assessment measures. The measurements of these risk and protective factors are described in **Supplementary file 1**.

**Table 1.** Assessment measures at baseline and follow up

| | **Baseline** | **3 months** | **6 months** | **9 months** | **12months** |
|---|---|---|---|---|---|
| **RISK AND PROTECTIVE FACTORS** | | | | | |
| Smoking | Q | Q | Q | Q | Q |
| Physical inactivity (SQUASH, IPAQ) | Q | Q | Q | Q | Q |
| Mediterranean diet (FFQ) | Q | Q | Q | Q | Q |
| Alcohol consumption (FFQ) | Q | Q | Q | Q | Q |
| High cognitive activity (CRIq) | Q | Q | Q | Q | Q |
| Loneliness (de Jong Gierveld, 6-item) | Q | Q | Q | Q | Q |
| Cardiovascular diseases (CVD) | Q | Q | Q | Q | Q |
| Obesity (body weight, height) | Q+ PE | Q | Q | Q | Q+PE |
| Hypertension (SBD, DBP) | Q+PE | Q | Q | Q | Q+PE |



For peer review only - http://bmjopen.bmj.com/site/about/guidelines.xhtml

| | | | | | |
|---|---|---|---|---|---|
| High cholesterol (LDL, HDL, TC) | Q+FBS | Q | Q | Q | Q+FBS |
| Diabetes Mellitus (glucose, HbA1C) | Q+FBS | Q | Q | Q | Q+FBS |
| Renal dysfunction (eGFR) | Q+FBS | Q | Q | Q | Q+FBS |
| Depression (CES-D) | Q | Q | Q | Q | Q |

*SQUASH* Short Questionnaire to Assess Health-enhancing physical activity, *IPAQ* International Physical Activity Questionnaire, *FFQ* Food Frequency Questionnaire, *CRIq* Cognitive Reserve Index questionnaire (adapted), *CVD* Cardiovascular diseases, *SBP* Systolic Blood Pressure, *DBP* Diastolic Blood Pressure, *HDL* high-density lipoproteins, *LDL* low-density lipoproteins, *TC* total cholesterol, *HbA1C* Haemoglobin A1C, *eGFR* estimated Glomerular Filtration Rate, *CES-D* Centre for Epidemiological Studies Depression Scale

Q: Online questionnaire, PE: Physical examination, FBS: Fasting blood sample

**2a. Personal health profile**

After completion of the baseline dementia risk assessment (including the data entry of the physical examination and laboratory measurements), a personal health profile is automatically provided in the personal account of the participants (My Demin). The personal health profile gives an overview of the presence of the risk and protective factors for dementia. According to the Lifestyle for Brain Health (LIBRA) score, each risk and protective factor [15,42,43] is categorized into one of the following categories: 1) room for improvement, 2) remember to manage well, 3) keep this up (see **Table 2**). The "Keep this up" category represent factors that participants are currently managing well or diseases they do not have. The "Room for improvement" category represents the factors that could be improved by health behaviour change (e.g. quit smoking, become more physical active, change diet, drink less alcohol). The category "Remember to manage well" is assigned when a risk factor (i.e. cardiovascular disease, hypertension, high cholesterol, diabetes mellitus, renal dysfunction and depression) is present, but the disease is managed well as participants have regular meetings with their general practitioner for disease control (diabetes mellitus) or use medication for disease management (cardiovascular disease, hypertension, high cholesterol, renal dysfunction and depression) (see **Figure 1**).

**[INSERT FIGURE 1 ABOUT HERE]**



For peer review only - http://bmjopen.bmj.com/site/about/guidelines.xhtml

For peer review only

**Table 2.** Definition for the 3 categories in the personal health profile at baseline

| Modifiable risk factors | Keep this up | Remember to manage well | Room for improvement |
|---|---|---|---|
| **Diet** | MIND-diet score = 14 points | n.a. | MIND-diet score < 14 points |
| **Alcohol consumption** | Average number of units of alcohol per week ≤ 7 and number of units per day is: ≤ 3 for women or ≤ 4 for men | n.a. | Average number of units of alcohol per week > 7 or number of units per day is: > 3 for women or > 4 for men |
| **Cognitive activity** | paid working hours ≥ 24 or CRIq score ≥ 50 | n.a. | paid working hours < 24 and CRIq score < 50 |
| **Physical activity** | (MVPA / week ≥ 150 and Sitting time ≤ 8 hours / day) or (MVPA / week < 150 and sitting time < 4 hours / day) | n.a. | (Sitting time > 8 hours / day) or (Sitting time ≥ 4 hours / day and MVPA / week < 150) |
| **Smoking** | Past or never smoker | n.a. | Current smoker |
| **Loneliness** | De Jong Gierveld score < 2 | n.a. | De Jong Gierveld score ≥ 2 |
| **Cardiovascular diseases (CVD)** | no CVD | at least one CVD and receives medical treatment | at least one CVD and no medical treatment |
| **Weight** | BMI ≥ 18.5 and BMI < 25.0 | n.a. | BMI < 18.5 or BMI ≥ 25.0 |
| **Blood pressure** | DBP < 90 mmHg and SBP < 140 and | DBP < 90 mmHg and SBP < 140 and | DBP ≥ 90 mmHg or |



For peer review only - http://bmjopen.bmj.com/site/about/guidelines.xhtml

| | | | |
|---|---|---|---|
| | no medical treatment | medical treatment | SBP ≥ 140 mmHg |
| **Cholesterol** | (LDL ≤ 2.5 mmol/l and TC/HDL ≤ 8) and no medical treatment | (LDL ≤ 2.5 mmol/l and TC/HDL ≤ 8) and medical treatment | LDL > 2.5 mmol/l or TC/HDL > 8 |
| **Diabetes Mellitus** | glucose < 7.0 mmol and HbA1C ≤ 53 mmol/mol | (HbA1C ≤ 53 mmol/mol and medical treatment) or (glucose < 7.0 mmol and HbA1C > 53 mmol/mol and medical treatment) | (HbA1C > 53 mmol/mol and no medical treatment) or (glucose ≥ 7.0 mmol and HbA1C > 53 mmol/mol) or (glucose ≥ 7.0 mmol and HbA1C ≤ 53 mmol/mol and no medical treatment) |
| **Kidney** | eGFR ≥ 60 ml/min/1.73 $m^2$ | eGFR < 60 ml/min/1.73 $m^2$ and medical treatment | eGFR < 60 ml/min/1.73 $m^2$ and no medical treatment |
| **Depression** | CES-D < 16 points | CES-D ≥ 16 points and medical treatment | CES-D ≥ 16 points and no medical treatment |

*MIND-diet* Mediterranean-DASH Diet Intervention for Neurodegenerative Delay, *CRIq* Cognitive Reserve Index questionnaire, *MVPA* Moderate to vigorous physical activity, CVD Cardiovascular diseases, *BMI* Body mass index, *DBP* diastolic blood pressure, *SBP* systolic blood pressure, *LDL* low-density lipoproteins, *TC* total cholesterol, *HDL* high-density lipoproteins, *HbA1C* Haemoglobin A1C, *eGFR* estimated Glomerular Filtration Rate, *CES-D* Centre for Epidemiological Studies Depression Scale



For peer review only - http://bmjopen.bmj.com/site/about/guidelines.xhtml

**2b. Tailor-made online lifestyle advice for dementia risk reduction**

Participants also receive an online tailor-made lifestyle advice targeting risk factors associated with dementia and following the Dutch guidelines for a healthy diet, alcohol consumption, physical activity, diabetes mellitus, renal dysfunction and cardiovascular health including cholesterol levels and BMI [44–48]. For each risk and protective factor, information is given about (i) the norm (cut-off point for not having this risk factor), (ii) the association between the risk factor and dementia and (iii) lifestyle advice how to tackle this factor. The online lifestyle advice was tailored to the participants based on (i) the presence of risk factors, (ii) the strength of the association between the risk factors and dementia [15,42] and (iii) the stages of change of the health behaviour related risk factors (physical inactivity, diet, alcohol consumption, smoking behaviour, cognitive activity, social activity). The stages of change are determined by asking "Which statement fits best for you?", where each answer option reflects one of the following stages of change: pre-contemplation, contemplation, preparation, action and maintenance [28]. It is known that participants who are in the preparation and action stage are more willing to change their health behaviour, therefore lifestyle advice for these factors are given first [28].

In case medically relevant findings are found, including untreated diabetes mellitus (glucose ≥ 7.0 mmol/l or (glucose ≥ 6.1 mmol/l and HbA1C > 53 mmol/mol)), untreated renal dysfunction (estimated Glomerular Filtration Rate (eGFR) ≤ 60 ml/min/1.73 m2) and increased risk for developing cardiovascular diseases (CVD) (CVD risk ≥ 10% according to the Dutch SCORE formula [48]), participants receive, in addition to the online tailor-made lifestyle advice, a separate message in their personal inbox with the recommendation to contact their general practitioner to verify the results and discuss whether treatment is needed.

**Outcome measures and measurements**

Participants are invited to fill in the online questionnaire at baseline and four times (3, 6, 9 and 12 months after baseline measurement) during one year follow-up . Physical examination, including the fasting blood sample for direct laboratory measurements, is only done at baseline and 12 months after



For peer review only - http://bmjopen.bmj.com/site/about/guidelines.xhtml

314 baseline measurement (see **Supplementary file 2**). Data from the online questionnaires and physical examination are stored automatically in an electronic Case Report Form (eCRF) data management program, which is only accessible by the researchers involved in this study. Data from the direct laboratory measurement are entered manually in the electronic Case Report Form (eCRF) data management program. Every month, memory clinics are requested to provide information about 1) the number of eligible participants (e.g. new cases of AD and VD), 2) the number of envelopes that are given away, and 3) any difficulties with the recruitment of participants. In order to keep participating memory clinics involved in the study, every three months newsletters are sent around and memory clinics are contacted monthly to evaluate the uptake.

**Primary outcome**

The primary outcome is the difference in uptake (e.g. the percentage of eligible people that signed the online informed consent form and completed risk assessment of the total number of eligible people) between the active and passive recruitment strategy. The total number of eligible people in each recruitment group (active versus passive) are based on the number of new cases of AD or VD in all memory clinics during the recruitment period, assuming an average of one child per dementia patient receiving the envelope with the PIF including a login access number.

**Secondary outcomes**

Secondary outcomes include:

**1)** The change in Lifestyle for Brain Health (LIBRA) score. The LIBRA score has been validated among individuals in midlife and reflects an individual's potential to reduce their risk on developing late-onset dementia [42]. The LIBRA score consists of twelve currently known protective (i.e. Mediterranean diet, low/moderate alcohol consumption, cognitive activity) and risk factors (i.e. physical inactivity, smoking, cardiovascular diseases, hypertension, high cholesterol, diabetes mellitus, obesity, renal dysfunction, depression) for dementia (13, 14,31) and ranges from -5.9 (low risk for developing dementia) to 12.7 (high risk for developing dementia).



For peer review only - http://bmjopen.bmj.com/site/about/guidelines.xhtml

A one point increase in the LIBRA score is associated with a 19% higher risk for dementia [42,49]. The definitions and corresponding scores for the three protective and ten risk factors for dementia are described in **Table 3**.

**Table 3.** Definition of risk and protective factors for dementia in the LIBRA score and corresponding scores

| Modifiable risk factors | | **Definition** | **Score** |
|---|---|---|---|
| **Protective factors** | | | |
| **1** | High cognitive activity | Score ≥ 50 points on the Cognitive Reserve Index questionnaire (leisure time activities) (CRIq) or hours of paid work ≥ 24 hours | -3.2 |
| **2** | Mediterranean diet | MIND-diet score (0-14) = 14 points | -1.7 |
| **3** | Low/moderate alcohol consumption | Average number of glasses of alcohol a week ≤ 7 and number of glasses a day is:<br>≤ 3 glasses for women (no binge drinking)<br>≤ 4 glasses for men (no binge drinking) | -1.0 |
| **Risk factors** | | | |
| **4** | Cardiovascular diseases (CVD) | Presence of at least one of the follow diseases: history of angina pectoris, myocardial infarction, transient ischemic attacks, stroke or peripheral arterial diseases | +1.0 |
| **5** | Physical inactivity | Not fulfilling Dutch Norm for Physical activity defined as ≥ 150 min/week physical activity of moderate to vigorous intensity, measured with the SQUASH questionnaire | +1.1 |
| **6** | Renal dysfunction | Estimated glomerular filtration rate ≤ 60 ml/min/1.73 | +1.1 |
| **7** | Diabetes Mellitus | Glucose (capillary blood) > 7.0 mmol/l or HbA1c > 53 mmol/mol | +1.3 |
| **8** | High cholesterol | LDL > 2.5 mmol/l or TC/HDL ≥ 8 | +1.4 |
| **9** | Smoking | Current smoker | +1.5 |



For peer review only - http://bmjopen.bmj.com/site/about/guidelines.xhtml

| | | | |
|---|---|---|---|
| **10** | Obesity | BMI ≥ 30 | +1.6 |
| **11** | Hypertension | SBP > 140 mmHg or DBP > 90 mmHg | +1.6 |
| **12** | Depression | Score ≥ 16 points on the Centre for Epidemiologic Studies Depression scale (CES-D) | +2.1 |

*LDL* low-density lipoproteins, *TC* total cholesterol, *HDL* high-density lipoproteins, *BMI* Body mass index, *SBP* systolic blood pressure, *DBP* diastolic blood pressure

**2)** The change in the individual health behaviours, including physical activity (minutes of MVPA per week), diet (MIND-diet score; 0-14), alcohol consumption (number of glasses of alcohol per week), smoking behaviour (current smoker (yes/no) and number of cigarettes/cigars a day), cognitive activity (leisure-time cognitive activity score and number of hours paid work), loneliness (overall loneliness score; 0-6) and social activity (number of contacts per two weeks) and their stage of change over time. The stages of change are categorized into pre-contemplation (1), contemplation (2), preparation (3), action (4) and maintenance (5) [28].

**3)** Changes in beliefs and attitudes with regard to dementia risk reduction are measured using the Motivation to Change Lifestyle and Health Behaviour for Dementia Risk Reduction Scale (MCLHB-DRR scale) [50,51]. The MCLHB-DRR scale is based on the Health Belief Model [27], which explains health-related behaviours. Seven subscales of the Health Belief Model were included: perceived susceptibility, perceived severity, perceived benefits, perceives barriers, cues to action, general health motivation and self-efficacy. Participants are asked to rate all items on a 5-point Likert scale, ranging from strongly disagree (score=1) to strongly agree (score=5). A minimum score of 23 and a maximum score of 115 can be achieved. A higher score reflects a higher motivation to change their lifestyle and health behaviour for dementia risk reduction. The Dutch version of the MCLHB-DRR scale, consisting of 23 items, has shown to be valid in the Dutch general population aged between 30 and 80 years old (unpublished observations, see **Supplementary file 3**).

For peer review only

366 **4)** Percentage of participants that indicated in the questionnaire that they have followed up the tailor-
367 made online lifestyle advice ("On what risk factors did you receive lifestyle advice?" and "Did you
368 follow up the tailor-made lifestyle advice since the last questionnaire (with regard to [risk factor])"? ,
369 but also the percentage of participants that indicated that they have followed up the advice to consult
370 their General Practitioner ("Did you have contact with your general practitioner after receiving
371 feedback on the risk and protective factors?").
372

373 **Statistical analyses**

374 First, descriptive characteristics will be explored. The difference in uptake between the two
375 recruitment strategies will be examined using multilevel logistic regression analyses in order to correct
376 for clustering at memory clinic level. We will calculate the percentage with the corresponding 95%
377 confidence interval (CI) and use an alpha of 0.05 to test statistical significance.
378 The effectiveness of the online tailor-made lifestyle program for dementia risk reduction will be
379 determined by, firstly comparing the change in LIBRA score, the individual risk factors and the
380 MCLHB-DRR score between the active and passive recruitment strategy, and secondly comparing
381 participants of the Demin study (active and passive recruitment strategy) to a control group consisting
382 of Lifelines participants (large population-based cohort study (n> 167.000)) (www.lifelines.nl)[52] in
383 outcome. Lifelines participants (age 40 – 60 years) with a parent with dementia will be matched (using
384 propensity scores) on non-modifiable risk factors (age, gender and education) for dementia to
385 participants of the Demin. Subsequently, multilevel analyses will be performed to examine the change
386 in the LIBRA score and the individual health behaviours over time. In addition, possible confounding
387 and interaction effects will be identified and corrected for in the analysis. We will calculate relative
388 risks (RR) with 95% confidence intervals (CI) and use an alpha of 0.05 to test significance.
389

390 **Adverse events**

391 The risk classification of this intervention is considered negligible, since only information and health
392 advice is provided. Serious adverse events as a result of the intervention are not expected, thus no data
393 safety and monitoring board is installed. Potential participants are informed about possible adverse



For peer review only - http://bmjopen.bmj.com/site/about/guidelines.xhtml

events. For example dementia risk assessment may help raising the awareness of their susceptibility in order to motivate health behaviour change [27], however risk assessment could also have an unfavourable effect. Participants may become anxious about developing dementia and could experience more stress if they receive their health profile. Therefore, participants are clearly informed that the presence or absence of risk and protective factors is not a reassurance that they will develop dementia later in life. Furthermore, participants are informed that there is the possibility that unexpected medical findings can be found. In this case, participants receive a separate message in their personal inbox with the recommendation to contact their general practitioner to verify the results (hypertension, high cholesterol, renal dysfunction, diabetes) and discuss whether treatment is needed. Participants may consider online risk assessment as a privacy risk. In this study, all personal information is kept separately from the research data, and participants use a SMS-tan code to login in their personal account.

**Ethics and dissemination**

This study is approved by the Dutch ministry of Health, Welfare and Sport according to the Dutch Population Screening Act. All participants give informed consent to participate in this study, by signing an electronic informed consort form using SMS-tan (see **Supplementary file 4**). Authorship will be allocated using the guidelines for authorship defined by the International Committees of Medical Journal Editors (ICMJE) [53]. The results of the trial will be submitted to an international peer-reviewed journal and presented at national and international conferences.

**Acknowledgements** The authors would like to thank the Board of Directors and the staff members of the participating memory clinics for the local approval and collaboration to conduct this multicentre study: Albert Schweitzer hospital (Dordrecht), Gelre hospital (Apeldoorn), University Medical Centre Groningen (Groningen), Medical Centre Leeuwarden (Leeuwarden), Nij Smellinghe (Drachten), Isala Zwolle (Zwolle), Martini hospital (Groningen), Haga hospital (Den Haag), Scheper hospital (Emmen), Refaja hospital (Stadskanaal), St. Jans Gasthuis (Weert), University Medical Centre Utrecht (Utrecht), Reinier de Graaf Gasthuis (Delft), Maxima Medical Centre (Eindhoven), Radboud

For peer review only



For peer review only - http://bmjopen.bmj.com/site/about/guidelines.xhtml

University Medical Centre (Nijmegen), TweeSteden hospital (Tilburg), Erasmus Medical Centre (Rotterdam), Ommelanden Hospital Groningen (Scheemda), Rijnstate hospital (Arnhem and Zevenaar).

**Collaborators** The members of the Demin consortium are: Elske Marije Abma (MD), Ameen Abu-Hanna (PhD), Erik Buskens (MD, PhD), Jürgen Claassen (MD, PhD), Peter Paul De Deyn (MD, PhD), Theo Feitsma (MD), Liesbeth Hempenius (MD, PhD), Jan Hoogmoed (MD), Ad Kamper (MD, PhD), Dineke Koek (MD, PhD), Jolijn Kragt (MD, PhD), Joep Lagro (MD, PhD), Els Lambooij (MD), Marc Langedijk (MD), Els Maeckelberghe (PhD), Francesco Mattace Raso (MD, PhD), Marieke Meinardi (MD, PhD), Richard Oude Voshaar (MD, PhD), Fransje Reesink (MD, PhD), Sophia de Rooij (MD, PhD), Antoinette Scheepmaker (MD), Nynke Smidt (PhD), Petra Spies (MD), Diana Taekema (MD, PhD), Jos Verkuyl (MD), Ralf Vingerhoets (MD), Joyce Vrijsen (MSc), Andrea de Winter (PhD).

**Author contributions** All authors are part of the Demin consortium. Nynke Smidt is the Principal Investigator. Joyce Vrijsen wrote the final manuscript. All authors contributed to the design of the study and have read, adjusted and approved the final version of the manuscript.

**Funding** This study was supported by grants from the Netherlands Organisation for Health Research and Development (ZonMw), subprogram prevention program (project number: 531002008).

**Competing interests** None declared

**Data availability** The data collected during this study will be available from the corresponding author upon reasonable request.

**Patient consent** Obligatory

**Ethics approval** This study is approved by the Dutch ministry of Health, Welfare and Sport according to the population screening act. In addition, all participating memory clinics approved the study.

**Supplementary files**

Supplementary file 1 Measures Dementia Risk Assessment

Supplementary file 2 Overview of measurements at baseline and follow up

Supplementary file 3 Manuscript in submission



For peer review only

For peer review only - http://bmjopen.bmj.com/site/about/guidelines.xhtml

450 Supplementary file 4 Consent form model



For peer review only - http://bmjopen.bmj.com/site/about/guidelines.xhtml

For peer review only

## REFERENCES

1 World Health Organization. Dementia: a public health priority. *World Heal Organ* 2012;:112.

2 Prince M, Wimo A, Guerchet M, *et al.* World Alzheimer Report 2015: The Global Impact of Dementia - An analysis of prevalence, incidence, cost and trends. *Alzheimer's Dis Int* 2015;:84. doi:10.1111/j.0963-7214.2004.00293.x

3 Wimo A, Guerchet M, Ali G-C, *et al.* The worldwide costs of dementia 2015 and comparisons with 2010. *Alzheimer's Dement* 2017;**13**:1–7. doi:10.1016/j.jalz.2016.07.150

4 Norton S, Matthews FE, Barnes DE, *et al.* Potential for primary prevention of Alzheimer's disease: an analysis of population-based data. *Lancet Neurol* 2014;**13**:788–94. doi:10.1016/S1474-4422(14)70136-X

5 Sindi S, Mangialasche F, Kivipelto M. Advances in the prevention of Alzheimer's Disease. *F1000Prime Rep* 2015;**7**:50. doi:10.12703/P7-50

6 Livingston G, Sommerlad A, Orgeta V, *et al.* The Lancet Commissions Dementia prevention, intervention, and care. Published Online First: 2017. doi:10.1016/S0140-6736(17)31363-6

7 Fratiglioni L, Launer LJ, Andersen K, *et al.* Incidence of dementia and major subtypes in Europe: A collaborative study of population-based cohorts. *Neurology* 2000;**54**:1–10.

8 Rizzi L, Rosset I, Roriz-Cruz M. Global Epidemiology of Dementia: Alzheimer's and Vascular Types. *Biomed Res Int* 2014;**2014**:1–8. doi:10.1155/2014/908915

9 Scarabino D, Gambina G, Broggio E, *et al.* Influence of family history of dementia in the development and progression of late-onset Alzheimer's disease. *Am J Med Genet Part B Neuropsychiatr Genet* 2016;**171**:250–6. doi:10.1002/ajmg.b.32399

10 van Exel E, Eikelenboom P, Comijs H, *et al.* Vascular factors and markers of inflammation in offspring with a parental history of late-onset Alzheimer disease. *Arch Gen Psychiatry* 2009;**66**:1263–70. doi:10.1001/archgenpsychiatry.2009.146

11 Muñoz M, Pong-Wong R, Canela-Xandri O, *et al.* Evaluating the contribution of genetics and familial shared environment to common disease using the UK Biobank. *Nat Genet* 2016;**48**:980–3. doi:10.1038/ng.3618

12 Borenstein AR, Copenhaver CI, Mortimer JA. Early-Life Risk Factors for Alzheimer Disease.

For peer review only



For peer review only - http://bmjopen.bmj.com/site/about/guidelines.xhtml

*Alzheimer Dis Assoc Disord* 2006;**20**:63–72. doi:10.1097/01.wad.0000201854.62116.d7

13 Andersson L, Stanich J. Life events and their impact on health attitudes and health behavior. *Arch Gerontol Geriatr* 1996;**23**:163–77. doi:10.1016/0167-4943(96)00716-9

14 Wolters FJ, van der Lee SJ, Koudstaal PJ, *et al.* Parental family history of dementia in relation to subclinical brain disease and dementia risk. *Neurology* 2017;**88**:1642–9. doi:10.1212/WNL.0000000000003871

15 Deckers K, van Boxtel MPJ, Schiepers OJG, *et al.* Target risk factors for dementia prevention: a systematic review and Delphi consensus study on the evidence from observational studies. *Int J Geriatr Psychiatry* 2015;**30**:234–46. doi:10.1002/gps.4245

16 de Bruijn RF, Bos MJ, Portegies ML, *et al.* The potential for prevention of dementia across two decades: the prospective, population-based Rotterdam Study. *BMC Med* 2015;**13**:132. doi:10.1186/s12916-015-0377-5

17 Ngandu T, Lehtisalo J, Solomon A, *et al.* A 2 year multidomain intervention of diet, exercise, cognitive training, and vascular risk monitoring versus control to prevent cognitive decline in at-risk elderly people (FINGER): a randomised controlled trial. *Lancet (London, England)* 2015;**385**:2255–63. doi:10.1016/S0140-6736(15)60461-5

18 van Charante EPM, Richard E, Eurelings LS, *et al.* Effectiveness of a 6-year multidomain vascular care intervention to prevent dementia (preDIVA): a cluster-randomised controlled trial. *Lancet* 2016;**388**:797–805. doi:10.1016/S0140-6736(16)30950-3

19 Vellas B, Carrie I, Gillette-Guyonnet S, *et al.* MAPT Study: A Multidomain Approach for Preventing Alzheimer's Disease: Design and Baseline Data. *J Prev Alzheimer's Dis* 2014;**1**:13–22.

20 Singh-Manoux A, Kivimaki M, Glymour MM, *et al.* Timing of onset of cognitive decline: results from Whitehall II prospective cohort study. *BMJ* 2012;**344**:d7622. doi:10.1136/BMJ.D7622

21 Sperling RA, Aisen PS, Beckett LA, *et al.* Toward defining the preclinical stages of Alzheimer's disease: recommendations from the National Institute on Aging-Alzheimer's Association workgroups on diagnostic guidelines for Alzheimer's disease. *Alzheimers Dement* 2011;**7**:280–92. doi:10.1016/j.jalz.2011.03.003

For peer review only



For peer review only - http://bmjopen.bmj.com/site/about/guidelines.xhtml

22 Webb TL, Joseph J, Yardley L, *et al.* Using the internet to promote health behavior change: a systematic review and meta-analysis of the impact of theoretical basis, use of behavior change techniques, and mode of delivery on efficacy. *J Med Internet Res* 2010;**12**:e4. doi:10.2196/jmir.1376

23 van Stralen MM, de Vries H, Mudde AN, *et al.* The long-term efficacy of two computer-tailored physical activity interventions for older adults: main effects and mediators. *Health Psychol* 2011;**30**:442–52. doi:10.1037/a0023579

24 Schulz DN, Kremers SPJ, Vandelanotte C, *et al.* Effects of a web-based tailored multiple-lifestyle intervention for adults: a two-year randomized controlled trial comparing sequential and simultaneous delivery modes. *J Med Internet Res* 2014;**16**:e26. doi:10.2196/jmir.3094

25 Jahangiry L, Montazeri A, Najafi M, *et al.* An interactive web-based intervention on nutritional status, physical activity and health-related quality of life in patient with metabolic syndrome: a randomized-controlled trial (The Red Ruby Study). *Nutr Diabetes* 2017;**7**:e240. doi:10.1038/nutd.2016.35

26 Murray E. Web-based interventions for behavior change and self-management: potential, pitfalls, and progress. *Med 20* 2012;**1**:e3. doi:10.2196/med20.1741

27 Janz NK, Becker MH. The Health Belief Model: A Decade Later. *Heal Educ Behav* 1984;**11**:1–47. doi:10.1177/109019818401100101

28 Prochaska JO, Velicer WF. The transtheoretical model of health behavior change. *Am J Health Promot* 1997;**12**:38–48.

29 Hagger MS, Chatzisarantis NLD. Integrating the theory of planned behaviour and self-determination theory in health behaviour: A meta-analysis. *Br J Health Psychol* 2009;**14**:275–302. doi:10.1348/135910708X373959

30 Bandura A. Social Cognitive Theory: An Agentic Perspective. *Annu Rev Psychol* 2001;**52**:1–26. doi:10.1146/annurev.psych.52.1.1

31 de Vries H. An Integrated Approach for Understanding Health Behavior; The I-Change Model as an Example. *Psychol Behav Sci Int J* 2017;**2**. doi:10.19080/pbsij.2017.02.555585

32 Webb TL, Joseph J, Yardley L, *et al.* Using the internet to promote health behavior change: a

For peer review only

systematic review and meta-analysis of the impact of theoretical basis, use of behavior change techniques, and mode of delivery on efficacy. *J Med Internet Res* 2010;**12**:e4. doi:10.2196/jmir.1376

33 Koopmans B, Nielen MM, Schellevis FG, *et al.* Non-participation in population-based disease prevention programs in general practice. *BMC Public Health* 2012;**12**:856. doi:10.1186/1471-2458-12-856

34 Centraal Bureau voor de Statistiek. Percentage hoger opgeleiden van 15 laar en ouder - Bevolkingskern (2011). 2011.http://www.cbsinuwbuurt.nl/#bevolkingskern2011_percentage_inwoners_hogere_opleiding (accessed 18 Feb 2019).

35 Donner A, Birkett N, Buck C. Randomization by cluster. *Am J Epidemiol* 1981;**114**:906–14. doi:10.1093/oxfordjournals.aje.a113261

36 Kul S, Vanhaecht K, Panella M. Intraclass correlation coefficients for cluster randomized trials in care pathways and usual care: hospital treatment for heart failure. *BMC Health Serv Res* 2014;**14**:84. doi:10.1186/1472-6963-14-84

37 Rutterford C, Copas A, Eldridge S. Methods for sample size determination in cluster randomized trials. *Int J Epidemiol* 2015;**44**:1051–67. doi:10.1093/ije/dyv113

38 Meppelink CS, Weert JC van, Haven CJ, *et al.* The Effectiveness of Health Animations in Audiences With Different Health Literacy Levels: An Experimental Study. *J Med Internet Res* 2015;**17**. doi:10.2196/jmir.3979

39 Mayer R. Multimedia Learning. In: Ross BH, ed. *The psychology of learning and motivation*. San Diego: : Academic Press 2002. 85–139.

40 Paivio A. *Mental representations : a dual coding approach*. Oxford University Press 1986.

41 Jones CR, Taylor K, Poston L, *et al.* Validation of the Welch Allyn 'Vital Signs' oscillometric blood pressure monitor. *J Hum Hypertens* 2001;**15**:191–5. doi:10.1038/sj.jhh.1001141

42 Schiepers OJG, Köhler S, Deckers K, *et al.* Lifestyle for Brain Health (LIBRA): a new model for dementia prevention. *Int J Geriatr Psychiatry* Published Online First: February 2017. doi:10.1002/gps.4700



For peer review only - http://bmjopen.bmj.com/site/about/guidelines.xhtml

43 Vos SJB, van Boxtel MPJ, Schiepers OJG, *et al.* Modifiable Risk Factors for Prevention of Dementia in Midlife, Late Life and the Oldest-Old: Validation of the LIBRA Index. *J Alzheimer's Dis* 2017;**58**:537–47. doi:10.3233/JAD-161208

44 De Grauw W, De Leest K, Schenk P, *et al.* NHG-Standaard Chronische Nierschade. *TPO - Prakt* 2018;**13**:26–9. doi:10.1007/s12503-018-0065-y

45 Health Council of the Netherlands. Richtlijnen goede voeding 2015. Den Haag: 2015.

46 Health Council of the Netherlands. Beweegrichtlijnen 2017. Den Haag: 2017.

47 Rutten G, De Grauw W, Nijpels G, *et al.* NHG-Standaard Diabetes mellitus type 2. 2013.

48 NHG-Standaard Cardiovasculair risicomanagement(Tweede herziening). *Huisarts Wet* 2012;**1**:14–28.

49 Deckers K, Köhler S, van Boxtel M, *et al.* Lack of associations between modifiable risk factors and dementia in the very old: findings from the Cambridge City over-75s cohort study. *Aging Ment Health* 2017;:1–7. doi:10.1080/13607863.2017.1280767

50 Kim S, Sargent-Cox K, Cherbuin N, *et al.* Development of the motivation to change lifestyle and health behaviours for dementia risk reduction scale. *Dement Geriatr Cogn Dis Extra* 2014;**4**:172–83. doi:10.1159/000362228

51 Beaton DE, Bombardier C, Guillemin F, *et al.* Guidelines for the Process of Cross-Cultural Adaptation of Self-Report Measures. *Spine (Phila Pa 1976)* 2000;**25**:3186–91. doi:10.1097/00007632-200012150-00014

52 Scholtens S, Smidt N, Swertz MA, *et al.* Cohort Profile: LifeLines, a three-generation cohort study and biobank. *Int J Epidemiol* 2015;**44**:1172–80. doi:10.1093/ije/dyu229

53 Editorial A. Recommendations for the Conduct, Reporting, Editing and Publication of Scholarly Work in Medical Journals. *Curr Pediatr* 2017;**16**:90–106. doi:10.15690/vsp.v16i2.1710

For peer review only



For peer review only - http://bmjopen.bmj.com/site/about/guidelines.xhtml

**License statement** I, the Submitting Author has the right to grant and does grant on behalf of all authors of the Work (as defined in the below author licence), an exclusive licence and/or a non-exclusive licence for contributions from authors who are: i) UK Crown employees; ii) where BMJ has agreed a CC-BY licence shall apply, and/or iii) in accordance with the terms applicable for US Federal Government officers or employees acting as part of their official duties; on a worldwide, perpetual, irrevocable, royalty-free basis to BMJ Publishing Group Ltd ("BMJ") its licensees and where the relevant Journal is co-owned by BMJ to the co-owners of the Journal, to publish the Work in BMJ Open and any other BMJ products and to exploit all rights, as set out in our licence.

The Submitting Author accepts and understands that any supply made under these terms is made by BMJ to the Submitting Author unless you are acting as an employee on behalf of your employer or a postgraduate student of an affiliated institution which is paying any applicable article publishing charge ("APC") for Open Access articles. Where the Submitting Author wishes to make the Work available on an Open Access basis (and intends to pay the relevant APC), the terms of reuse of such Open Access shall be governed by a Creative Commons licence – details of these licences and which Creative Commons licence will apply to this Work are set out in our licence referred to above.

For peer review only

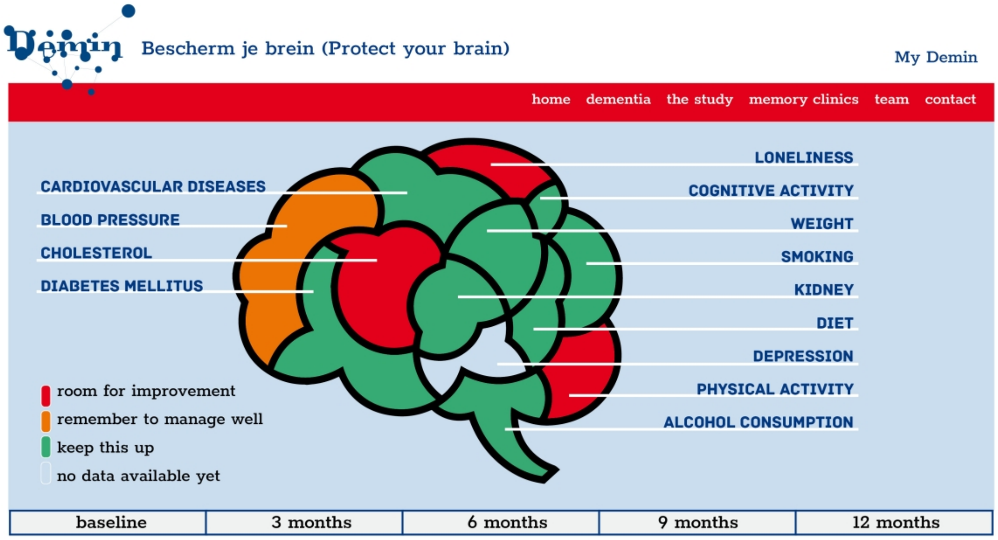


Figure 1. Example of a personal health profile

291x158mm (300 x 300 DPI)

**Supplementary file 1: Measures Dementia Risk Assessment**

Through the online questionnaire and physical examination, data on thirteen currently known protective (i.e. Mediterranean diet, low/moderate alcohol consumption, cognitive activity) and risk factors (i.e. physical inactivity, smoking, loneliness, cardiovascular diseases, hypertension, high cholesterol, diabetes mellitus, obesity, renal dysfunction, depression) for dementia are collected [1–3]. The measurements of these risk and protective factors are described below.

**Protective factors**

**Mediterranean diet**

The Mediterranean-DASH diet intervention for neurodegenerative delay (MIND) has shown to slow down cognitive decline [4] and to decrease the risk of developing AD [5]. Therefore, adherence to the MIND-diet is determined with a number of items of the Food Frequency Questionnaire (FFQ), which is a reliable and valid instrument to measure intake of a specified list of food items in the general populations [6,7]. The following healthy food groups of the MIND-diet were included in the questionnaire, such as vegetables (especially green leafy vegetables), nuts, berries, beans, whole grains, seafood, poultry, olive oil [4,5]. Also five unhealthy food groups of the MIND-diet including red meat, butter, cheese, sweets and fried/fast food were asked [4,5]. Based on the intake of the food groups, adherence to the MIND-diet is determined (0-14). A score of 14 represent good adherence to the MIND-diet *(See **Table 1** for the MIND-diet scoring table).*

For peer review only

**Table 1.** MIND-diet scoring table [5]

| MIND components | Recommended quantity | Max score |
|---|---|---|
| Whole grains | ≥ 3 serving spoons / day | 1 |
| Green leafy | ≥ 6 serving spoons / week | 1 |
| Other vegetables | ≥ 1 serving spoon / day | 1 |
| Berries (including other fruits) | ≥ 2 portions / week * | 1 |
| Red Meats and products | < 4 portions / week | 1 |

| | | |
|---|---|---|
| Fish | ≥ 1 portion / week | 1 |
| Poultry | ≥ 2 portions / week | 1 |
| Beans | > 3 serving spoons /week | 1 |
| Nuts | ≥ 5 portions /week * | 1 |
| Fast/ fried food | < 1time / week | 1 |
| Butter, margarine | < 1 teaspoon/ day | 1 |
| Cheese | < 1 slice / week | 1 |
| Pastries, sweets | < 5 portions / week | 1 |
| Olive Oil (used as primary oil) | yes | 1 |
| **Total score** | | **14** |

** One portion is a handful of the given component*

**Low/moderate alcohol consumption**

Alcohol consumption was measured using the FFQ [6], including questions regarding the frequency of alcohol use (e.g. no consumption last month, 1 day per month, 2-3 days per month, 1 day per week, 2-3 days per week, 4-5 days per week, 6-7 days per week) and the average number of glasses of alcohol per day (range from zero to more than twelve) was asked. Subsequently, the average number of glasses per month was calculated in order to classify participants into: (i) non-alcohol consumers, (ii) low/moderate alcohol consumers or (iii) excessive alcohol consumers [8]. Participants adhere to the national recommendations for no to low/moderate alcohol consumption, if participants drink one glass or less alcohol per day on average, without binge drinking (more than three glasses alcohol on one day for females and more than four glasses alcohol on one day for males)) [9].

**High cognitive activity**

Cognitive activity is assessed with the leisure time section of the Cognitive Reserve Index questionnaire (CRIq) (22). CRIq aims to measure cognitive reserve (CR), which is based on education, working activity and leisure time activity. For this study we are interested in the current cognitive

For peer review only

activities of the participants. Therefore, cognitive activity is determined by measuring working activity and leisure time activity. The frequency of eighteen leisure time activity is asked (e.g. (i) never, (ii) less than once a month, (iii) once a month, (iv) once every 2 weeks, (v) several times a week). Subsequently, a leisure time cognitive activity score is calculated, ranging from to 18 to 108, where a score of 50 or higher represent high cognitive activity (based on results of a survey on the knowledge, beliefs and attitudes towards dementia risk reduction among the general population of Groningen, see Table 2 and 3).

Additionally, participants are asked if they have a paid job and if so how many hours they spend on their job per week. High cognitive activity is defined as (i) working at least 24 hours per week or (ii) a leisure time cognitive activity score of at least 50.

**Table 2.** Cognitive activity (leisure time) scores stratified for education level and having a paid job in a survey conducted among the general population in Groningen

| **Education level** | **Work** | **Leisure time score** | |
|---|---|---|---|
| | | mean(SD) | (min-max) |
| Low (n=105) | no work (n=75) | 39.57 (11.16) | 13 – 63 |
| | work (n=30) | 41.73 (11.12) | 25 – 68 |
| Middle (n=154) | no work (n=72) | 47.03 (9.95) | 26 – 76 |
| | work (n=82) | 45.20 (9.49) | 25 – 64 |
| High (n=390) | no work (n= 135) | 51.93 (10.19) | 18 – 75 |
| | work (n=255) | 48.32 (8.97) | 23 – 74 |

**Table 3.** Cognitive activity (leisure time) scores stratified for education level and having a paid job in a survey conducted among the general population in Groningen (subgroup: 40 – 60 year old)

| **Education level** | **Work** | **Leisure time score** | |
|---|---|---|---|
| | | mean(SD) | (min-max) |

For peer review only

| | | | |
|---|---|---|---|
| Low (n=29) | no work (n=7) | 39.71 (9.67) | 21 – 49 |
| | work (n=22) | 40.50 (10.52) | 25 – 58 |
| Middle (n=68) | no work (n=9) | 43. 89 (13.15) | 26 – 66 |
| | work (n=59) | 46.34 (8.87) | 25 – 64 |
| High (n=140) | no work (n= 16) | 50.56 (10.59) | 37 – 69 |
| | work (n=124) | 49.91 (9.24) | 23 – 74 |

For peer review only

**Risk factors**

**Physical inactivity**

Physical activity levels are determined using the Short Questionnaire to Asses Health enhancing physical activity (SQUASH), a self-reported questionnaire and commonly used instrument in the Netherlands to assess physical activity [10]. The SQUASH questionnaire has shown to be valid and reliable in measuring physical activity among the Dutch population [11–14]. The SQUASH questionnaire includes questions on multiple activities referring to an average week in the last month, including actively commuting (walking, cycling) to (voluntary) work or school, physical activity at (voluntary) work or school, household activities and leisure time activities, including walking, cycling, gardening and sports. Participants were asked to fill in how many days a week they engaged in the activities (frequency), the average time per day spent on each activity (hours and minutes; duration) and the intensity at which they did the activity (low, moderate, high) [10]. A standardized methodology was followed to calculate physical activity levels. Briefly, results from the SQUASH questionnaire are automatically converted to minutes per week spent in light (LPA) and moderate to vigorous (MVPA) intensity activities based on Metabolic Equivalent Tasks (METs) derived from the Ainsworth’s compendium of physical activity [15]. Physical activity levels are divided into the following categories: 0 minutes MVPA per week, 0 to 149 minutes MVPA per week, 150 to 299 minutes MVPA per week and 300 minutes MVPA per week and more. Physical inactivity is defined as less than 150 minutes per week MVPA [16].

Additionally, the questionnaire contained information on sitting behaviour, which is divided into sitting during transportation, working hours, watching television or using the computer at home. Participants are asked to fill in the number of hours and minutes on an average day in the past seven days during the week and on an average day during the weekend. This is similar to the sitting measure of the International Physical Activity Questionnaire (IPAQ) which has shown to be valid and reliable [17]. Sitting time was divided into the following 4 categories: (i) less than 4 hours a day, (ii) 4 to 8 hours a day, (iii) 8 to 11 hours a day and (iv) at least 11 hours a day or more [18]. Prolonged sitting time was defined as sitting at least for 8 hours a day or more.

Participants are physically inactive if they (i) are sitting on average more than 8 hours a day, irrespective of the physical activity, or (ii) are sitting on average 4 hours or more a day and are less active than 150 minutes MVPA per week.

**Smoking**

Participants are asked three questions to measure smoking behaviour: (i) whether they have smoked in the past month, and (ii) whether they have smoked in the past, for at least one year [19]. Smoking behaviour is categorized into non-smoker, past smoker and current smoker. Current smokers are defined as people who reported smoking in the past month. Past smokers reported smoking for at least one year, but did not smoke in the past month.

**Loneliness**

Loneliness is measured using De Jong Gierveld Loneliness Scale , which is a reliable and valid instrument to measure emotional, social and overall loneliness [20]. Possible answers on this 6-item scale are: (i) yes!, (ii) yes, (iii) more or less, (iv), no, (v) no!. The overall loneliness score is calculated by counting the neutral and negative ("no!", "no", or "more or less") answers on items 4, 5 and 6 (social loneliness score) and by counting the positive ("more or less"", "yes" or "yes!") answers on items 1,2 and 3 (emotional loneliness score). Subsequently, the overall loneliness score is categorized into: (i) not lonely (0-1), (ii) moderate lonely (2-4), (iii) severe lonely (5-6). Loneliness is defined as an overall loneliness score of 2 or higher [20].

For peer review only

**Cardiovascular diseases**

Participants are asked whether they have suffered or still suffer from one of the following cardiovascular diseases: angina pectoris, myocardial infarction, transient ischemic attack (TIA), stroke or peripheral arterial diseases (yes/no). Presence of a cardiovascular disease is defined as having at least one of the above mentioned diseases.

**Hypertension**

Hypertension is determined based on the blood pressure measurement in which the systolic and diastolic blood pressure is measured both three times consecutively. The average of the second and the third measurement is used to determine the presence of hypertension. Hypertension is present: (i) if the systolic blood pressure is higher than 140 mmHg or diastolic blood pressure is higher than 90 mmHg [21], or (ii) if participants indicate that they receive medication (i.e. diuretics, beta blockers, ACE-inhibitors, angiotensin 2 antagonists and calcium antagonists) for their hypertension .

**High cholesterol**

High cholesterol is defined based on direct laboratory measurements using the fasting blood samples and self-reported questionnaires. High cholesterol is present if (i) the Low Density Lipoprotein (LDL) is higher than 2.5 mmol/l or (ii) the ratio of total cholesterol (TC) and High Density Lipoprotein (HDL) is higher than 8 mmol/l [22] or (iii) participants indicate that they receive medication (i.e. simvastinin, atorvastatin, rosuvastatin, pravastatin, ezetimib) to lower their cholesterol levels.

**Diabetes Mellitus**

The presence of diabetes mellitus (or impaired blood glucose levels) is based on direct laboratory measurements using the fasting blood samples and self-reported questionnaires. Diabetes Mellitus is defined as: (i) glucose (fasting capillary blood) of 7.0 mmol/l or higher, or (ii) glucose (fasting capillary blood) lower than 7.0 mmol/l accompanied by HbA1C levels higher than 53 mmol/mol [23].

For peer review only

HbA1C provides additional information on the average blood glucose levels during the previous month, while glucose may differ during the day [23].

**Obesity**

Body weight and body height are measured during physical examination in order to determine their Body Mass Index (BMI=kg/$m^2$)[24]. Obesity is present if BMI is 30 kg/$m^2$ or higher [25].

**Renal dysfunction**

The presence of renal dysfunction is based on direct laboratory measurements (serum creatinine levels) using the fasting blood samples and self-reported questionnaires [26]. Subsequently, the estimated glomerular filtration rate (eGFR) is calculated using the 2009 CKD-EPI creatinine equation [27,28] in order to determine participant's renal function [28]. Renal dysfunction is present if (i) eGFR is lower than 60 ml/min/1.73 $m^2$ [29], or (ii) participants indicate that they receive medical treatment for the established renal dysfunction.

**Depression**

The level of depressive symptoms is measured using the Centre for Epidemiologic Studies Depression scale (CES-D). The CES-D consists of 20 items and is a reliable and valid tool to measure the current level of depressive symptoms in the general population [30]. Answer options for each item are: rarely or none of the time (0), some or a little of the time (1), occasionally or a moderate amount of time (2), and most of all of the time (3). Total score varying from 0 to 60, indicates the level of depressive symptoms, a higher score reflects a higher level of depressive symptoms. Depression is defined as (i) having a score of 16 or higher [31], or (ii) participants indicate that they receive medical treatment for their depressive symptoms.

For peer review only

**REFERENCES**

1 Livingston G, Sommerlad A, Orgeta V, *et al.* The Lancet Commissions Dementia prevention, intervention, and care. Published Online First: 2017. doi:10.1016/S0140-6736(17)31363-6

2 Deckers K, van Boxtel MPJ, Schiepers OJG, *et al.* Target risk factors for dementia prevention: a systematic review and Delphi consensus study on the evidence from observational studies. *Int J Geriatr Psychiatry* 2015;**30**:234–46. doi:10.1002/gps.4245

3 Schiepers OJG, Köhler S, Deckers K, *et al.* Lifestyle for Brain Health (LIBRA): a new model for dementia prevention. *Int J Geriatr Psychiatry* Published Online First: 28 February 2017. doi:10.1002/gps.4700

4 Morris MC, Tangney CC, Wang Y, *et al.* MIND diet slows cognitive decline with aging. *Alzheimer's Dement* 2015;**11**:1015–22. doi:10.1016/j.jalz.2015.04.011

5 Morris MC, Tangney CC, Wang Y, *et al.* MIND diet associated with reduced incidence of Alzheimer's disease. *Alzheimer's Dement* 2015;**11**:1007–14. doi:10.1016/j.jalz.2014.11.009

6 Molag ML, de Vries JHM, Duif N, *et al.* Selecting informative food items for compiling food-frequency questionnaires: comparison of procedures. *Br J Nutr* 2010;**104**:446–56. doi:10.1017/S0007114510000401

7 Siebelink E, Geelen A, de Vries JHM. Self-reported energy intake by FFQ compared with actual energy intake to maintain body weight in 516 adults. *Br J Nutr* 2011;**106**:274–81. doi:10.1017/S0007114511000067

8 Anstey KJ, Mack HA, Cherbuin N. Alcohol Consumption as a Risk Factor for Dementia and Cognitive Decline: Meta-Analysis of Prospective Studies. *Am J Geriatr Psychiatry* 2009;**17**:542–55. doi:10.1097/JGP.0b013e3181a2fd07

9 Gezondheidsraad. Richtlijnen goede voeding 2015 Gezondheidsraad. https://www.gezondheidsraad.nl/sites/default/files/201524_richtlijnen_goede_voeding_2015.pdf (accessed 20 Mar 2017).

For peer review only

10 Wendel-Vos G, Schuit A. SQUASH: Short Questionnaire to Assess Health enhancing physical activity. Bilthoven, Netherlands: 2004.

11 Wagenmakers R, van den Akker-Scheek I, Groothoff JW, *et al.* Reliability and validity of the short questionnaire to assess health-enhancing physical activity (SQUASH) in patients after total hip arthroplasty. *BMC Musculoskelet Disord* 2008;**9**:141. doi:10.1186/1471-2474-9-141

12 de Hollander EL, Zwart L, de Vries SI, *et al.* The SQUASH was a more valid tool than the OBiN for categorizing adults according to the Dutch physical activity and the combined guideline. *J Clin Epidemiol* 2012;**65**:73–81. doi:10.1016/j.jclinepi.2011.05.005

13 Wendel-Vos G. Reproducibility and relative validity of the short questionnaire to assess health-enhancing physical activity. *J Clin Epidemiol* 2003;**56**:1163–9. doi:10.1016/S0895-4356(03)00220-8

14 Nicolaou M, Gademan MGJ, Snijder MB, *et al.* Validation of the SQUASH Physical Activity Questionnaire in a Multi-Ethnic Population: The HELIUS Study. *PLoS One* 2016;**11**:e0161066. doi:10.1371/journal.pone.0161066

15 Ainsworth BE, Haskell WL, Herrmann SD, *et al.* 2011 Compendium of Physical Activities: a second update of codes and MET values. *Med Sci Sports Exerc* 2011;**43**:1575–81. doi:10.1249/MSS.0b013e31821ece12

16 Gezondheidsraad. Beweegrichtlijnen 2017. Den Haag: 2017. file://zkh/dfs/Gebruikers22/VrijsenJ/Data/Downloads/Beweegrichtlijnen_2017.pdf (accessed 14 Sep 2017).

17 CRAIG CL, MARSHALL AL, SJÖSTRÖM M, *et al.* International Physical Activity Questionnaire: 12-Country Reliability and Validity. *Med Sci Sport Exerc* 2003;**35**:1381–95. doi:10.1249/01.MSS.0000078924.61453.FB

18 van der Ploeg HP, Chey T, Korda RJ, *et al.* Sitting Time and All-Cause Mortality Risk in 222 497 Australian Adults. *Arch Intern Med* 2012;**172**:494. doi:10.1001/archinternmed.2011.2174


For peer review only

19 World Health Organization. Global Adult Tobacco Survey Collaborative Group. Tobacco Questions for Surveys: A Subset of Key Questions from the Global Adult Tobacco Survey (GATS) 2 nd Edition. 2011;:6–9. doi:10.1007/s00406-006-0651-9

20 Gierveld JDJ, Tilburg T Van. A 6-Item Scale for Overall, Emotional, and Social Loneliness. *Res Aging* 2006;**28**:582–98. doi:10.1177/0164027506289723

21 NHG-Standaard Cardiovasculair risicomanagement(Tweede herziening). *Huisarts Wet* 2012;**1**:14–28.https://www.nhg.org/standaarden/volledig/cardiovasculair-risicomanagement (accessed 20 Jun 2017).

22 NHG-Standaard Cardiovasculair risicomanagement(Tweede herziening). *Huisarts Wet* 2012;**1**:14–28.

23 Rutten G, De Grauw W, Nijpels G, *et al.* NHG-Standaard Diabetes mellitus type 2. 2013.

24 World Health Organization (WHO). Body mass index - BMI. http://www.euro.who.int/en/health-topics/disease-prevention/nutrition/a-healthy-lifestyle/body-mass-index-bmi (accessed 6 May 2019).

25 Van Binsbergen J, Langens F, Dapper A, *et al.* NHG-Standaard Obesitas | NHG. 2010. https://www.nhg.org/standaarden/volledig/nhg-standaard-obesitas (accessed 4 Dec 2019).

26 Perrone RD, Madias NE, Levey AS. Serum creatinine as an index of renal function: new insights into old concepts. *Clin Chem* 1992;**38**.http://clinchem.aaccjnls.org/content/38/10/1933.short (accessed 4 Apr 2017).

27 Levey AS, Stevens LA, Schmid CH, *et al.* A new equation to estimate glomerular filtration rate. *Ann Intern Med* 2009;**150**:604–12. doi:10.7326/0003-4819-150-9-200905050-00006

28 Levey AS, Inker LA, Coresh J. GFR estimation: From physiology to public health. *Am J Kidney Dis* 2014;**63**:820–34. doi:10.1053/j.ajkd.2013.12.006

29 De Grauw W, De Leest K, Schenk P, *et al.* NHG-Standaard Chronische Nierschade. *TPO - Prakt* 2018;**13**:26–9. doi:10.1007/s12503-018-0065-y

For peer review only

30 Smarr KL, Keefer AL. Measures of depression and depressive symptoms: Beck Depression Inventory-II (BDI-II), Center for Epidemiologic Studies Depression Scale (CES-D), Geriatric Depression Scale (GDS), Hospital Anxiety and Depression Scale (HADS), and Patient Health Questionna. *Arthritis Care Res (Hoboken)* 2011;**63**:S454–66. doi:10.1002/acr.20556
31 Smarr KL, Keefer AL. Measures of depression and depressive symptoms: Beck Depression Inventory-II (BDI-II), Center for Epidemiologic Studies Depression Scale (CES-D), Geriatric Depression Scale (GDS), Hospital Anxiety and Depression Scale (HADS), and Patient Health Questionna. *Arthritis Care Res (Hoboken)* 2011;**63**:S454–66. doi:10.1002/acr.20556

For peer review only

**Supplementary file 2. Overview of assessment measures at baseline and follow up**

| **Table 1.** Assessment measures at baseline and follow up | | | | | |
|---|---|---|---|---|---|
| | **Baseline** | **3 months** | **6 months** | **9 months** | **12months** |
| **GENERAL INFORMATION** | | | | | |
| Age, gender, ethnicity, education and postal code | Q | | | | |
| Participation in the Lifelines cohort | Q | | | | |
| Medical family history | Q | | | | |
| Health literacy (S-TOFHLA, 3-items) | Q | | | | |
| **RISK AND PROTECTIVE FACTORS** | | | | | |
| Smoking | Q | Q | Q | Q | Q |
| Physical inactivity (SQUASH, IPAQ sitting measure) | Q | Q | Q | Q | Q |
| Mediterranean diet (FFQ) | Q | Q | Q | Q | Q |
| Alcohol consumption (FFQ) | Q | Q | Q | Q | Q |
| High cognitive activity (CRIq adapted) | Q | Q | Q | Q | Q |
| Loneliness (de Jong Gierveld, 6-items) | Q | Q | Q | Q | Q |
| Cardiovascular diseases (CVD) | Q | Q | Q | Q | Q |
| Obesity (body weight, height) | Q+ PE | Q | Q | Q | Q+PE |
| Hypertension (SBD, DBP) | Q+PE | Q | Q | Q | Q+PE |
| High cholesterol (LDL, HDL, TC) | Q+BS | Q | Q | Q | Q+BS |
| Diabetes Mellitus[1] (glucose, | Q+BS | Q | Q | Q | Q+BS |

For peer review only - http://bmjopen.bmj.com/site/about/guidelines.xhtml

| | | | | | |
|---|---|---|---|---|---|
| HbA1C) | | | | | |
| Renal dysfunction (eGFR) | Q+BS | Q | Q | Q | Q+BS |
| Depression (CES-D) | Q | Q | Q | Q | Q |
| **OTHER PARAMETERS** | | | | | |
| Medical treatment of disease | Q | Q | Q | Q | Q |
| Motivation to change lifestyle (MCLHB-DRR) | Q | Q | Q | Q | Q |
| Stages of change | Q | Q | Q | Q | Q |
| Hearing problems | Q | Q | Q | Q | Q |
| Subjective stress (LDI) | Q | | | | Q |
| Memory complaints | Q | | | | |
| Quality of life (2 items of SF36, VAS-score) | Q | | | | Q |
| Perceived living environment | Q | | | | Q |
| Compliance lifestyle advice per individual health behaviour | | Q | Q | Q | Q |
| Compliance advice contact with GP | | Q | Q | Q | Q |

*SQUASH* Short Questionnaire to Assess Health-enhancing physical activity, *IPAQ* International Physical Activity Questionnaire, *FFQ* Food Frequency Questionnaire, *CRIq* Cognitive Reserve Index questionnaire, *CVD* Cardiovascular diseases, *SBP* Systolic Blood Pressure, *DBP* Diastolic Blood Pressure, *HDL* high-density lipoproteins, *LDL* low-density lipoproteins, *TC* total cholesterol, *HbA1C* Hemoglobin A1C, *eGFR* estimated Glomerular Filtration Rate, *CES-D* Centre for Epidemiological Studies Depression Scale, *MCLHB-DRR* Motivation to Change Lifestyle and Health Behavior for Dementia Risk Reduction Scale, LDI Long-term Difficulties Inventory, *SF36* Short Form 36 items, *VAS* Visual Analogue Scale

Q: Online questionnaire; PE: Physical examination; BS: Blood sample

For peer review only

**TITLE**

Cross-cultural validation of the Motivation to Change Lifestyle and Health Behaviours for Dementia Risk Reduction scale in the Dutch general population

**AUTHORS**

Tessa Joxhorst[1], Joyce Vrijsen[1]*, Jacobien Niebuur[1] and Nynke Smidt[1,2]

[1] Department of Epidemiology, University of Groningen, University Medical Centre Groningen, the Netherlands

[2] Department of Geriatrics, University of Groningen, University Medical Centre Groningen, Groningen, The Netherlands.

*Corresponding author. University Medical Centre Groningen, Department of Epidemiology, Hanzeplein 1, PO Box 30 001, FA40, 9700 RB Groningen, The Netherlands. E-mail: j.vrijsen@umcg.nl

For peer review only



For peer review only - http://bmjopen.bmj.com/site/about/guidelines.xhtml

## ABSTRACT

BACKGROUND: This study aims to translate and validate the Motivation to Change Lifestyle and Health Behaviours for Dementia Risk Reduction (MCLHB-DRR) scale in the Dutch general population.

METHODS: A random sample of Dutch residents aged between 30 and 80 years old were invited to complete an online questionnaire including the translated MCLHB-DRR scale. Exploratory and confirmatory factor analyses (EFA and CFA) were conducted to assess construct validity. Cronbach's alpha was calculated to assess internal consistency.

RESULTS: 618 participants completed the questionnaire. EFA and Cronbach's alpha showed that four items were candidate for deletion. CFA confirmed that deleting these items led to an excellent fit (RMSEA = 0.043, CFI = 0.960, TLI = 0.951, $\chi^2/df = 2.130$). Cronbach's alpha ranged from 0.69 to 0.93, indicating good internal consistency.

CONCLUSION: The Dutch MCLHB-DRR scale demonstrated to have good validity to assess the health beliefs and attitudes towards dementia risk reduction.



## KEY WORDS

Dementia risk reduction

Behaviour change

Lifestyle change

Cross-cultural validation




For peer review only - http://bmjopen.bmj.com/site/about/guidelines.xhtml

## BACKGROUND

Dementia is a major public health concern for society. The prevalence of dementia increases rapidly, from 47 million cases worldwide in 2015 to an estimated 131 million cases in 2050 (1). The increasing number of dementia patients carries a high socioeconomic burden for society because of the associated rising health care costs and the burdensome effects of the disease on patients, their families and caregivers (2). The World Health Organization highlights dementia as a public health priority and advocates for action to decrease its social and economic burden (3).

The increase in the number of dementia patients is mainly attributable to population ageing, since age is the most important risk factor for dementia (1,4). In addition to non-modifiable risk factors for dementia like age and genetics, several studies suggested potential modifiable risk factors that are associated with dementia and in particular AD and vascular dementia (5-8). Recently, the evidence for these potential modifiable risk factors for dementia was summarized by Livingston et al. (2017) (8). They found that 35% of all dementia cases worldwide are attributable to nine modifiable risk factors and recommended to start interventions including more childhood education, promotion of exercise, reduction of smoking, maintaining social engagement and management of hypertension, diabetes, obesity, depression and hearing loss. It is estimated that these interventions might delay or prevent a third of all dementia cases (8).

Currently, there is no cure for dementia, so prevention of dementia is the key in fighting this disease. A diversity of multi-domain lifestyle interventions is conducted in elderly and people at risk for dementia in order to decrease the risk of developing dementia, including the



For peer review only - http://bmjopen.bmj.com/site/about/guidelines.xhtml

FINGER study, MAPT study, preDIVA study and HATICE trial (9-12). The aforementioned studies showed some evidence for effectiveness of a multi-domain approach to prevent elderly from cognitive decline, but further research is needed (13-15). Although behavioural change is crucial for dementia risk reduction, changing behaviour is complex and many factors are related to the chances for successfully altering behaviour according to different social cognitive theories and models (16-20). Measuring beliefs and attitudes towards lifestyle adaptations for dementia risk reduction may help to predict a person's willingness to change lifestyle and behaviour aiming to reduce one's risk of developing dementia.

The Motivation to Change Lifestyle and Health Behaviours for Dementia Risk Reduction (MCLHB-DRR) scale is developed in Australia and measures the beliefs and attitudes towards dementia and dementia risk reduction (21). The MCLHB-DRR scale was based on the Health Belief Model (HBM), since the HBM turned out to be the best-suited social cognitive model for dementia risk reduction (21). The HBM suggests that engagement in health-promoting behaviour is defined by a person's subjective risk assessment of getting a condition and how serious this condition and its consequences are, the perceived benefits and barriers of performing this behaviour, a stimulus to trigger this behaviour, the desire to achieve an outcome, and the confidence in one's ability to take action (22). The MCLHB-DRR scale consists of 27 items and includes all seven subscales of the HBM: perceived susceptibility, perceived severity, perceived benefits, perceived barriers, cues to action, general health motivation and self-efficacy. The MCLHB-DRR scale is considered to be valid and reliable in Australians aged 50 years and older (21). To our knowledge, the MCLHB-DRR scale has not yet been cross-culturally validated in any other language since its development in Australia.



For peer review only - http://bmjopen.bmj.com/site/about/guidelines.xhtml

There is currently no instrument available to measure attitudes and beliefs towards lifestyle and health behavioural changes for dementia risk reduction in the Netherlands. The MCLHB-DRR scale could be used to measure the attitudes and beliefs towards lifestyle adaptations for dementia risk reduction in the Dutch population. This induces the opportunity to use this scale in developing tailored interventions or education programs focused on lifestyle adjustments for dementia risk reduction. Therefore, the aim of the current study is to translate and validate the MCLHB-DRR scale in the Dutch general population aged between 30 and 80 years old.

For peer review only



For peer review only - http://bmjopen.bmj.com/site/about/guidelines.xhtml

## METHODS

### Study design and participants

MCLHB-DRR data were collected among a random sample of residents of the municipality of Groningen aged between 30 and 80 years old. From the 101,518 residents of the municipality of Groningen, 4,500 residents stratified for age (30-39, 40-49, 50-59, 60-69 and 70-80 years old) and gender (male, female) were randomly selected by a staff member of the municipality of Groningen. This staff member was not involved in the data collection nor data analyses of this study. The selected 4,500 residents were invited by mail to participate in an online survey about 'Lifestyle and dementia'. The translated MCLHB-DRR scale was the last part of this survey. The survey was built in Survey Monkey (SurveyMonkey Inc., San Mateo, California, VS, www.surveymonkey.com). In order to increase the response rate, participants were able to win a voucher of 20 Euros. Furthermore, participants were offered to receive the survey results on population level if they would finish the complete survey.

A pilot study was conducted to test the final version of the online survey 'Lifestyle and dementia'. A total of 25 people aged 30 to 80 years who were living outside the municipality of Groningen participated in the pilot study. They were recruited within the network of the research team members. Results of the pilot study did not lead to any changes in the final Dutch version of the MCLHB-DRR scale.

### Questionnaire

The MCLHB-DRR scale is a self-reported questionnaire aiming to measure the attitudes and beliefs towards dementia and dementia risk reduction (21). The MCLHB-DRR scale consists of 27 items covering seven subscales: perceived susceptibility (4 items), perceived severity (5



For peer review only - http://bmjopen.bmj.com/site/about/guidelines.xhtml

items), perceived benefits (4 items), perceived barriers (4 items), cues to action (4 items), general health motivation (4 items) and self-efficacy (2 items). Items are answered on a 5 point Likert-scale, ranging from ‘strongly disagree’ (score = 1) to ‘strongly agree’ (score = 5) (21).

**Scale translation**

For the translation of the MCLHB-DRR scale, we used the method of Beaton et al. (2000) (23). Briefly, the MCLHB-DRR scale was translated into Dutch by three native Dutch translators, independently. Two of these translators were familiar with the concepts being examined in the questionnaire (the so-called informed translators). The third translator was not familiar with the content or concepts of the questionnaire (uninformed translator). All items, instructions and the response options of the questionnaire were translated.

Subsequently, the three translated versions were synthesized to one Dutch version by the informed translators. The discrepancies between the three translated versions were discussed between the informed translators, taking the original questionnaire into account.

Secondly, the synthesized Dutch version of the questionnaire was translated back into English by two independent native English speakers (uninformed translators). Both translators were not involved in the translation of the questionnaire from English to Dutch and were blinded to the original version of the questionnaire.

Afterwards all versions of the questionnaire, including the original version, the three translated versions, the synthesized Dutch version, the two back translations and all written reports about the decisions being made during the translation process were discussed by the informed translators. Special attention was paid to achieve semantic, idiomatic, experiential and conceptual equivalence between the source and target version of the questionnaire. After



For peer review only - http://bmjopen.bmj.com/site/about/guidelines.xhtml

a comprehensive review of all versions of the questionnaire, consensus about the pre-final version of the questionnaire was reached.

Finally, the two back translations were combined in the best possible way and this version was send to the developers of the original scale (21) to check whether the meaning of the translated items was equivalent to the meaning of the original items. Their feedback was discussed, resulting in a small change in the translation of item 20 and item 25. Afterwards, the Dutch final version of the MCLHB-DRR scale was established.

**Statistical analysis**

First, study population characteristics and characteristics of the MCLHB-DRR scale were calculated using descriptive statistics. Second, exploratory factor analysis (EFA) was performed. Maximum Likelihood estimation or Principal Axis Factoring was used depending on whether the data was roughly normally distributed or non-normally distributed, respectively. Oblique rotation was used as rotation method (delta (0)), which is taking into account correlations among factors. If the correlations between all factors were below 0.32, we changed to Varimax rotation (24). Items that did not have a correlation of 0.20 or higher with any of the other items were deleted immediately. Items with a high correlation (> 0.70) with any of the other items, were considered carefully. Items with a factor loading below 0.30 on any of the factors were deleted immediately. Deletion of an item was considered if the item did not load sufficiently on one of the factors (< 0.50) or if an item had a cross-loading greater than 0.30 (25).

Internal consistency of the subscales was evaluated by item-total correlations and Cronbach's alpha. Deletion of an item was considered when the item-total correlation of an item was below 0.30 (25). Cronbach's alpha values of 0.70 or higher were considered acceptable (26).

For peer review only

168 In addition, confirmatory factor analysis (CFA) was conducted. The following fit indices and their required levels were used to verify construct validity of the MCLHB-DRR scale: Root Mean Squared Error of Approximation (RMSEA) < 0.08 (moderate) and < 0.05 (excellent), Comparative Fit Index (CFI) and Tucker-Lewis Index (TLI) > 0.90 (moderate) and > 0.95 (excellent) and $\chi^2$/df < 3.0 (27,28).

EFA was performed using IBM SPSS Statistics software version 23 (SPSS Inc., Chicago, IL, USA). CFA was analysed using Stata version 13 (StataCorp. 2013. Stata Statistical Software: Release 13. College Station, TX: StataCorp LP.). Participants who did not complete the whole MCLHB-DRR scale were excluded from data analysis.

**Ethics**

The Medical Ethics Review Board of the UMCG concluded that this study was not subject to the Medical Research Involving Human Subjects Act. Respondents participated in this study voluntary. They could withdraw at any time without any consequence or penalty. Data were collected and analysed anonymously. All participants provided informed consent.



For peer review only - http://bmjopen.bmj.com/site/about/guidelines.xhtml

## RESULTS

### Participant recruitment

From the 4,500 selected potential participants, 621 participants completed the survey. The data of the 'cues to action' subscale of three participants were missing. These participants were excluded, leaving a total of 618 participants for data analysis.

### Characteristics of the study population

The characteristics of the study population (n = 618) are presented in **Table 1**. The mean age of the participants was 57.3 ± 13.5 years and more than half of the participants were female (54%) and were married or had a registered partnership (54%). Most participants completed tertiary education (59%), followed by upper secondary education (24%), lower secondary education (14%) and elementary education (2%). About 58% of the participants were currently working. The percentages of participants knowing a relative with dementia or a non-relative with dementia were 45% and 21%, respectively.

### Analysis of the psychometric characteristics of the MCLHB-DRR scale

### Scale descriptives

The average MCLHB-DRR total scale score was 75.1 ± 11.1 (SD), ranging from 30 to 115. The total scale score was approximately normally distributed, although the scores were moderately leptokurtic (skewness = -0.394, kurtosis = 0.994). The mean subscale scores were 10.1 ± 2.7 (range = 4 to 18) for perceived susceptibility, 13.9 ± 3.7 (range = 5 to 25) for perceived severity, 12.6 ± 2.9 (range = 4 to 20) for perceived benefits, 8.0 ± 2.5 (range = 4 to 15) for perceived barriers, 10.2 ± 3.1 (range = 4 to 19) for cues to action, 14.5 ± 2.3 (range =



For peer review only - http://bmjopen.bmj.com/site/about/guidelines.xhtml

4 to 20) for general health motivation and 5.8 ± 1.7 (range = 2 to 10) for self-efficacy. All subscale scores were approximately normally distributed. Item response scores of the MCLHB-DRR scale ranged from 1.9 ± 0.8 (item 15) to 4.1 ± 0.7 (item 24).

**Exploratory factor analysis**

EFA analysis with extraction method Maximum Likelihood and Oblimin rotation was used to assess the number of factors, because the data were roughly normally distributed. First, a seven factor solution was evaluated as the original MCLHB-DRR scale consists of seven subscales. All items had an inter-item correlation greater than 0.20 with at least one of the other items. The correlation between item 1 and item 2 was 0.86 ($p < 0.001$), the correlation between item 1 and item 3 was 0.77 ($p < 0.001$) and the correlation between item 2 and item 3 was 0.82 ($p < 0.001$). Although these items had high inter-item correlations, they still measured something else ($r < 0.90$) and loaded on their intended factors. Therefore, none of these items was deleted. All other inter-item correlations did not exceed 0.70. The Bartlett's test of sphericity was significant, indicating that the data was adequate for factor analysis ($p < 0.001$). The first seven factors had eigenvalues greater than 1.00. The eigenvalues and the cumulative percentages of explained variance of the first seven factors in brackets were 5.86 (21.7%), 2.94 (32.6%), 2.52 (41.9%), 2.10 (49.7%), 1.56 (55.5%), 1.23 (60.0%) and 1.03 (63.9%), respectively. The scree plot also suggested a seven factor model.

Almost all items loaded on their intended subscales and did not have any significant cross-loadings. However, item 10 did not have a factor loading greater than 0.30 on any of the factors. Therefore, item 10 was deleted. Item 26 had a significant cross-loading (cross-loading = 0.37) on the perceived benefits subscale. Furthermore, items 4, 5, 7, 9, 13, 25 and 26 had factor loadings between 0.30 and 0.50 on their intended factors (**Table 2**). Inclusion of these



For peer review only - http://bmjopen.bmj.com/site/about/guidelines.xhtml

items was assessed in the next step by evaluating the internal consistency of their subscale. The inter-scale correlations between the subscale factors ranged from -0.13 to 0.51.

**Internal consistency**

Item-total correlation analysis showed that all items were positively correlated with the total MCLHB-DRR scale score. Item-total correlations ranged from 0.15 to 0.67. The item-total correlations of item 14 ($r = 0.28$), item 22 ($r = 0.15$), item 23 ($r = 0.26$) and item 24 ($r_s = 0.19$) were lower than 0.30. All other items had an item-total correlation above 0.30. Cronbach's alpha values were $\alpha = 0.86$ for perceived susceptibility, $\alpha = 0.76$ for perceived severity, $\alpha = 0.76$ for perceived benefits, $\alpha = 0.77$ for perceived barriers, $\alpha = 0.84$ for cues to action, $\alpha = 0.64$ for general health motivation and $\alpha = 0.81$ for self-efficacy, all indicating good internal consistency. Cronbach's alpha of the perceived susceptibility, perceived benefits and general health motivation subscales could be elevated by deleting an item. Items 4, 13 and 25 already had low factor loadings (factor loadings < 0.50) and were therefore eliminated. After deletion of these items, Cronbach's alpha values were $\alpha = 0.93$ for perceived susceptibility, $\alpha = 0.80$ for perceived benefits and $\alpha = 0.69$ for general health motivation. Cronbach's alpha of all subscales could not be raised any further after deleting these items (**Table 3**).

**Confirmatory factor analysis**

CFA with Maximum Likelihood method was conducted to explore the model fit of the MCLHB-DRR scale. A seven factor model with 23 items (excluding items 4, 10, 13 and 25) was evaluated with CFA (model 1). All fit indices were indicating an excellent fit (RMSEA = 0.043, CFI = 0.960, TLI = 0.951, $\chi^2$/df = 2.130) (**Table 4**). The factor loadings ranged from 0.395 to 0.978 and were all statistically significant (**Table 5**). A seven factor model including



For peer review only - http://bmjopen.bmj.com/site/about/guidelines.xhtml

257 all 27 items (model 2) showed a moderate fit (RMSEA = 0.053, CFI = 0.920, TLI = 0.907,
258 $\chi^2$/df = 2.743), indicating model 1 had a better fit to the data than model 2.

For peer review only



For peer review only - http://bmjopen.bmj.com/site/about/guidelines.xhtml

## DISCUSSION

We demonstrated that the Dutch version of the MCLHB-DRR scale consisting of 23 items is a valid instrument to measure the beliefs and attitudes towards lifestyle and health behavioural changes for dementia risk reduction in people aged between 30 and 80 years old. EFA showed that nearly all items loaded on their intended factors without cross-loadings. Cronbach's alpha varied from 0.69 to 0.93, indicating good internal consistency. CFA confirmed that a seven factor model including 23 items (without items 4, 10, 13 and 25) had an excellent fit to the data (RMSEA = 0.043, CFI = 0.960, TLI = 0.951, $\chi^2$/df = 2.130).

Items 4, 10, 13 and 25 had low factor loadings and were therefore not included in the final Dutch version of the instrument. This could possibly be explained by differences in knowledge of dementia and dementia prevention between residents of Australia and the Netherlands. Australia is leading in the field of dementia prevention with the world first publicly-funded dementia prevention program (29). This could lead to increased public awareness about dementia and the prevention of dementia in Australia. In general, the Australian population scored higher on all subscales of the MCLHB-DRR scale. Besides, differences in cultural beliefs about general health, health behaviours and the prestige of health professionals may play a role. Another possible explanation is the age difference between the Australian and Dutch study populations (21). The study population of the Australian study was 50 years and older whereas our population was between 30 and 80 years. People aged below 50 years might be less scared to develop dementia in the upcoming 10 years and might be less concerned about their health in comparison to people aged 50 years and over. However, our sensitivity analysis in which we only included people aged 50 years and over did not change our results in any way. Deficiencies in the translation process



For peer review only - http://bmjopen.bmj.com/site/about/guidelines.xhtml

could be a third explanation. The translation of item 10 slightly changed, as the part of the sentence 'may give me something that I never thought of' is not included in the Dutch translation.

**Strengths and limitations**

To our knowledge, this was the first study that validated the MCLHB-DRR scale in the Dutch general population. A major strength of the current study was the random sample, as the information letter was send to randomly selected residents of the municipality of Groningen. Another strength is the adequate sample size, consisting of a total number of 618 participants. Besides, we followed formal guidelines presented by Beaton et al. (2000) during the translation process (23).

This study also had certain limitations. The response rate of the current study was 14%, which is relatively low. However, we used several methods which have shown to increase the response rate to electronic surveys, such as a lottery to win a voucher, an offer to receive survey results on population level, a personalised invitation letter, an easily accessible link to the survey and a deadline to complete the survey (30,31). In our study, 59% of the participants completed tertiary education, which is higher than the percentage completing tertiary education in Dutch residents aged 45 years and over (26 %) (32). Therefore, the sample is not fully representative for the Dutch general population.

**Recommendations for future research**

First, assessing reliability and responsiveness of the Dutch MCLHB-DRR scale would be a valuable addition for future research. Second, a part of the study population might not be familiar with the health behaviours that decrease the risk of developing dementia. Future research could consider informing participants about these health behaviours before filling in



For peer review only - http://bmjopen.bmj.com/site/about/guidelines.xhtml

the MCLHB-DRR scale. Further research should also examine the association between the motivation to change lifestyle and health behaviours for dementia risk reduction and actually conducting this behaviour in daily life.

**Implications**

This scale can be useful in developing and evaluating interventions aimed at dementia risk reduction in various ways. Firstly, this instrument might help to predict people who will comply with an intervention program aimed at dementia prevention. Secondly, this instrument can be used in developing tailored interventions based on a person's motivations and beliefs. For example, if an individual scores low on the perceived benefits subscale, it would be convenient to educate this individual about how changing lifestyle and health behaviours could reduce its risk of dementia. Finally, assessing the beliefs towards lifestyle and health behavioural changes in the community population of the Netherlands may help to develop media campaigns or education programs focused on dementia prevention.

**Conclusion**

In summary, we have demonstrated that the translated and adapted Dutch version of the MCLHB-DRR scale consisting of 23 items is a valid instrument to assess beliefs and attitudes towards dementia and dementia risk reduction in the Dutch general population aged between 30 and 80 years old. The MCLHB-DRR scale can be used in the development and evaluation of lifestyle interventions and media campaigns aimed at dementia risk reduction.



For peer review only - http://bmjopen.bmj.com/site/about/guidelines.xhtml

330 **LIST OF ABBREVIATIONS**

331

332 MCLHB-DRR: Motivation to Change Lifestyle and Health Behaviours for Dementia Risk
333 Reduction

334 EFA: Exploratory factor analyses

335 CFA: Confirmatory factor analyses

336 RMSEA: Root Mean Squared Error of Approximation

337 CFI: Comparative Fit Index

338 TLI: Tucker-Lewis Index

339 AD: Alzheimer's disease

340 HBM: Health Belief Model

341 SD: Standard deviation

342

For peer review only

## DECLARATIONS

**Ethics approval**

The Medical Ethics Review Board of the UMCG concluded that this study was not subject to the Medical Research Involving Human Subjects Act. Respondents participated in this study voluntary. They could withdraw at any time without any consequence or penalty. Data were collected and analysed anonymously.

**Patient consent** All participants provided informed consent.

**Availability of data and materials** The datasets analysed during the current study are available from the corresponding author on reasonable request.

**Competing interests** None declared

**Funding** This research did not receive any specific grant from funding agencies in the public, commercial, or not-for-profit sectors.

**Authors' contributions** All authors contributed to the design of the study. All co-authors read and approved the final manuscript.

**Acknowledgements**

The authors would like to thank Sarang Kim for her help during the translation process. The authors also gratefully acknowledge the municipality of Groningen for the selection of the potential participants.

For peer review only



For peer review only - http://bmjopen.bmj.com/site/about/guidelines.xhtml

# FIGURES AND TABLES

**Table 1**: Study population characteristics

| **Characteristic** | **All participants (N=618)**[1] |
|---|---|
| Age, years (mean ± SD) | 57.3 ± 13.5 |
| Gender (% male) | 281 (46%) |
| Marital status | |
| - Married/registered partnership | 336 (54%) |
| - Domestic partnership | 99 (16%) |
| - Living apart together (LAT) | 23 (4%) |
| - Single | 90 (15%) |
| - Widow/widower | 20 (3%) |
| - Divorced | 46 (7%) |
| - Others | 4 (1%) |
| Education | |
| - Elementary | 11 (2%) |
| - Lower secondary | 88 (14%) |
| - Upper secondary | 150 (24%) |
| - Tertiary | 363 (59%) |
| - Others | 6 (1%) |
| Working status (% currently working) | 357 (58%) |
| Relative with dementia | 276 (45%) |
| Non-relative with dementia | 132 (21%) |

[1] The numbers of participants (percentages) are shown unless otherwise stated.

For peer review only

**Table 2**: Exploratory factor analysis of the MCLHB-DRR scale (N=618, Maximum Likelihood with Oblimin rotation)

| | Factor 1 | Factor 2 | Factor 3 | Factor 4 | Factor 5 | Factor 6 | Factor 7 |
|---|---|---|---|---|---|---|---|
| Q1 My chances of developing dementia are great | -0.02 | **0.90** | 0.00 | -0.03 | -0.03 | 0.03 | 0.04 |
| Q2 I feel that my chances of developing dementia in the future are high | 0.00 | **0.97** | 0.02 | -0.04 | -0.03 | 0.00 | -0.00 |
| Q3 There is a strong possibility that I will develop dementia | 0.04 | **0.86** | -0.03 | 0.01 | 0.02 | -0.03 | 0.04 |
| Q4 Within the next 10 years I will develop dementia | -0.04 | **0.33** | 0.07 | 0.25 | 0.07 | -0.04 | -0.12 |
| Q5 The thought of dementia scares me | 0.01 | 0.08 | 0.02 | **0.49** | 0.05 | 0.09 | 0.10 |
| Q6 When I think about dementia my heart beats faster | -0.06 | -0.00 | 0.10 | **0.81** | 0.01 | -0.02 | -0.04 |
| Q7 My feelings about myself would change if I develop dementia | 0.04 | -0.01 | -0.12 | **0.43** | -0.01 | 0.03 | 0.10 |
| Q8 When I think about dementia I feel nauseous | -0.03 | -0.03 | 0.03 | **0.80** | 0.06 | -0.05 | -0.12 |
| Q9 It would be more serious for me to develop dementia than if I developed other diseases | 0.03 | 0.05 | 0.06 | **0.45** | -0.04 | 0.00 | -0.03 |
| Q10 Information and advice from experts may give me something that I never thought of, and may reduce my chance of developing dementia | 0.15 | 0.01 | 0.18 | 0.17 | -0.09 | 0.01 | 0.20 |
| Q11 Changing my lifestyle and health habits can help me reduce my chance of developing dementia | 0.07 | 0.06 | 0.06 | 0.01 | -0.04 | -0.05 | **0.77** |
| Q12 I have a lot to gain by changing my lifestyle and health behaviour | -0.03 | 0.03 | 0.08 | 0.01 | 0.05 | -0.01 | **0.77** |
| Q13 Adapting to a healthier lifestyle and behaviour would prevent dementia for me | 0.13 | -0.06 | 0.10 | 0.06 | 0.10 | 0.01 | **0.38** |
| Q14 I am too busy to change my lifestyle and health habits | 0.02 | -0.03 | 0.00 | -0.02 | **0.61** | -0.05 | -0.01 |
| Q15 My financial situation does not allow me to change my lifestyle and behaviour | 0.02 | -0.02 | 0.06 | 0.05 | **0.62** | 0.05 | -0.07 |
| Q16 Family responsibilities make it hard for me to change my lifestyle and behaviour | -0.01 | 0.04 | -0.09 | -0.02 | **0.78** | 0.03 | 0.05 |
| Q17 Changing lifestyle and behaviour interferes with my schedule | -0.02 | -0.02 | 0.07 | -0.00 | **0.68** | -0.06 | 0.06 |



For peer review only - http://bmjopen.bmj.com/site/about/guidelines.xhtml

| | | | | | | | |
|---|---|---|---|---|---|---|---|
| Q18 Being forgetful makes me think I have to change my lifestyle and behaviour | 0.02 | 0.01 | **0.68** | -0.02 | 0.05 | 0.01 | -0.03 |
| Q19 Having risk factor(s) for dementia makes me think I have to change my lifestyle and behaviour | 0.01 | 0.03 | **0.81** | -0.03 | -0.01 | 0.01 | 0.04 |
| Q20 Learning more about dementia from the media makes me think I have to change my lifestyle and behaviour | -0.01 | -0.03 | **0.71** | 0.02 | -0.03 | 0.03 | 0.17 |
| Q21 Knowing family member(s) with dementia makes me think I have to change my lifestyle and behaviour | 0.07 | 0.04 | **0.64** | 0.03 | 0.08 | -0.02 | 0.00 |
| Q22 Nothing is as important to me as good health | -0.03 | -0.11 | 0.05 | 0.10 | -0.08 | **0.51** | -0.10 |
| Q23 I often think about my health | 0.00 | -0.01 | -0.03 | -0.02 | 0.03 | **0.85** | 0.01 |
| Q24 I think I have to pay attention to my own health | 0.07 | 0.05 | -0.05 | -0.09 | -0.01 | **0.63** | -0.00 |
| Q25 I am concerned about my health | -0.07 | 0.09 | 0.17 | 0.12 | 0.06 | **0.32** | 0.12 |
| Q26 I am certain that I can change my lifestyle and behaviour so I can reduce the risk of developing dementia | **0.47** | 0.03 | 0.16 | -0.05 | -0.03 | 0.03 | **0.37** |
| Q27 I am able to make differences that will change the risk of developing dementia | **1.02** | -0.00 | 0.02 | 0.03 | 0.04 | 0.02 | -0.07 |

The factor loadings greater than 0.30 are shown in bold. The boxes show the predicted subscales according to the results from Kim et al. 2014 (21)



For peer review only - http://bmjopen.bmj.com/site/about/guidelines.xhtml

**Table 3**: Internal consistency of the subscales

| Subscale | Dutch MCLHB-DRR scale; N = 618 | | | | English MCLHB-DRR scale; N = 617 | | | |
|---|---|---|---|---|---|---|---|---|
| | No. of items | Range of scores | Mean ± SD | α | No. of items | Range of scores | Mean ± SD | α |
| Perceived susceptibility | 4 | 4-18 | 10.1±2.7 | 0.86[2] | 4 | 4-19 | NK | 0.86 |
| Perceived severity | 5 | 5-25 | 13.9±3.7 | 0.76 | 5 | 5-25 | NK | 0.73 |
| Perceived benefits | 3[1] | 3-15 | 9.1±2.3 | 0.76[3] | 4 | 4-20 | NK | 0.69 |
| Perceived barriers | 4 | 4-15 | 8.0±2.5 | 0.77 | 4 | 4-20 | NK | 0.74 |
| Cues to action | 4 | 4-19 | 10.2±3.1 | 0.84 | 4 | 4-20 | NK | 0.68 |
| General health motivation | 4 | 4-20 | 14.5±2.3 | 0.64[4] | 4 | 4-20 | NK | 0.61 |
| Self-efficacy | 2 | 2-10 | 5.8±1.7 | 0.81 | 2 | 2-10 | NK | 0.66 |

[1] Item 10 is deleted.

[2] Cronbach's alpha elevated to 0.93 if item 4 was deleted.

[3] Cronbach's alpha elevated to 0.80 if item 13 was deleted.

[4] Cronbach's alpha elevated to 0.69 if item 25 was deleted.

*Abbreviations:* α = Cronbach's alpha, MCLHB-DRR = Motivation to Change Lifestyle and Health Behaviours for Dementia Risk Reduction, NK = not known.

For peer review only

**Table 4**: Goodness of fit indexes of MCLHB-DRR models

| **Index** | **Dutch MCLHB-DRR scale (model 1)** | **Dutch MCLHB-DRR scale (model 2)** | **English MCLHB-DRR scale** |
|---|---|---|---|
| RMSEA | 0.043 | 0.053 | 0.047 |
| CFI | 0.960 | 0.920 | 0.920 |
| TLI | 0.951 | 0.907 | NK |
| $\chi^2$/df | 2.130 | 2.743 | 2.380 |

Model 1 represents a seven factor model including 23 items (without items 4, 10, 13 and 25); model 2 represents a seven factor model including all 27 items.

*Abbreviations*: RMSEA = Root Mean Squared Error of Approximation, CFI = Comparative Fit Index, TLI = Tucker-Lewis Index, MCLHB-DRR = Motivation to Change Lifestyle and Health Behaviours for Dementia Risk Reduction, NK = not known.

For peer review only

**Table 5**: Confirmatory Factor Analysis report

| Subscales | Item | Factor Loading |
|---|---|---|
| Perceived severity | Q1 | 0.896* |
| | Q2 | 0.953* |
| | Q3 | 0.858* |
| Perceived severity | Q5 | 0.573* |
| | Q6 | 0.842* |
| | Q7 | 0.395* |
| | Q8 | 0.753* |
| | Q9 | 0.487* |
| Perceived benefits | Q11 | 0.842* |
| | Q12 | 0.797* |
| Perceived barriers | Q14 | 0.627* |
| | Q15 | 0.617* |
| | Q16 | 0.734* |
| | Q17 | 0.719* |
| Cues to action | Q18 | 0.671* |
| | Q19 | 0.825* |
| | Q20 | 0.793* |
| | Q21 | 0.717* |
| General health motivation | Q22 | 0.562* |
| | Q23 | 0.815* |
| | Q24 | 0.612* |
| Self-efficacy | Q26 | 0.978* |
| | Q27 | 0.702* |

Results are shown for model 1. *$p < 0.001$.



For peer review only - http://bmjopen.bmj.com/site/about/guidelines.xhtml

## REFERENCES

(1) Prince M, Wimo A, Guerchet M, Ali GC, Wu YT, Prina M. World Alzheimer Report 2015. The Global Impact of Dementia. Alzheimer's Disease International. Alzheimer's Disease International (ADI), London 2015.

(2) Wimo A, Guerchet M, Ali G, Wu Y, Prina AM, Winblad B, et al. The worldwide costs of dementia 2015 and comparisons with 2010. Alzheimer's & Dementia 2017;13(1):1-7.

(3) World Health Organization. Dementia: a public health priority. Dementia 2012.

(4) Ritchie K, Kildea D. Is senile dementia" age-related" or" ageing-related"?—evidence from meta-analysis of dementia prevalence in the oldest old. The Lancet 1995;346(8980):931-934.

(5) Verghese PB, Castellano JM, Holtzman DM. Apolipoprotein E in Alzheimer's disease and other neurological disorders. The Lancet Neurology 2011;10(3):241-252.

(6) Norton S, Matthews FE, Barnes DE, Yaffe K, Brayne C. Potential for primary prevention of Alzheimer's disease: an analysis of population-based data. The Lancet Neurology 2014;13(8):788-794.

(7) Deckers K, van Boxtel MP, Schiepers OJ, de Vugt M, Muñoz Sánchez JL, Anstey KJ, et al. Target risk factors for dementia prevention: a systematic review and Delphi consensus study on the evidence from observational studies. Int J Geriatr Psychiatry 2015;30(3):234-246.

(8) Livingston G, Sommerlad A, Orgeta V, Costafreda SG, Huntley J, Ames D, et al. Dementia prevention, intervention, and care. The Lancet 2017;390(10113):2673-2734.

(9) Kivipelto M, Solomon A, Ahtiluoto S, Ngandu T, Lehtisalo J, Antikainen R, et al. The Finnish geriatric intervention study to prevent cognitive impairment and disability (FINGER): study design and progress. Alzheimer's & Dementia 2013;9(6):657-665.

(10) Vellas B, Carrie I, Gillette-Guyonnet S, Touchon J, Dantoine T, Dartigues JF, et al. MAPT study: a multidomain approach for preventing Alzheimer's disease: design and baseline data. The journal of prevention of Alzheimer's disease 2014;1(1):13.

(11) Richard E, Van den Heuvel E, van Charante, Eric P Moll, Achthoven L, Vermeulen M, Bindels PJ, et al. Prevention of dementia by intensive vascular care (PreDIVA): a cluster-randomized trial in progress. Alzheimer Disease & Associated Disorders 2009;23(3):198-204.

(12) Richard E, Jongstra S, Soininen H, Brayne C, van Charante, Eric P Moll, Meiller Y, et al. Healthy Ageing Through Internet Counselling in the Elderly: the HATICE randomised controlled trial for the prevention of cardiovascular disease and cognitive impairment. BMJ open 2016;6(6):e010806.

(13) Ngandu T, Lehtisalo J, Solomon A, Levälahti E, Ahtiluoto S, Antikainen R, et al. A 2 year multidomain intervention of diet, exercise, cognitive training, and vascular risk monitoring versus control to prevent cognitive decline in at-risk elderly people (FINGER): a randomised controlled trial. The Lancet 2015;385(9984):2255-2263.



For peer review only - http://bmjopen.bmj.com/site/about/guidelines.xhtml

(14) Andrieu S, Guyonnet S, Coley N, Cantet C, Bonnefoy M, Bordes S, et al. Effect of long-term omega 3 polyunsaturated fatty acid supplementation with or without multidomain intervention on cognitive function in elderly adults with memory complaints (MAPT): a randomised, placebo-controlled trial. The Lancet Neurology 2017;16(5):377-389.

(15) van Charante, Eric P Moll, Richard E, Eurelings LS, van Dalen J, Ligthart SA, Van Bussel EF, et al. Effectiveness of a 6-year multidomain vascular care intervention to prevent dementia (preDIVA): a cluster-randomised controlled trial. The Lancet 2016;388(10046):797-805.

(16) Ajzen I. The theory of planned behavior. Organ Behav Hum Decis Process 1991;50(2):179-211.

(17) Bandura A. Self-efficacy conception of anxiety. Anxiety Res 1988;1(2):77-98.

(18) Prochaska JO, Velicer WF. The transtheoretical model of health behavior change. American journal of health promotion 1997;12(1):38-48.

(19) Janz NK, Becker MH. The health belief model: A decade later. Health Educ Q 1984;11(1):1-47.

(20) Locke EA, Latham GP. A theory of goal setting & task performance. : Prentice-Hall, Inc; 1990.

(21) Kim S, Sargent-Cox K, Cherbuin N, Anstey KJ. Development of the motivation to change lifestyle and health behaviours for dementia risk reduction scale. Dementia and geriatric cognitive disorders extra 2014;4(2):172-183.

(22) Champion VL, Skinner CS. The health belief model. Health behavior and health education: Theory, research, and practice 2008;4:45-65.

(23) Beaton DE, Bombardier C, Guillemin F, Ferraz MB. Guidelines for the process of cross-cultural adaptation of self-report measures. Spine 2000;25(24):3186-3191.

(24) Costello AB, Osborne JW. Best practices in exploratory factor analysis: Four recommendations for getting the most from your analysis. Practical assessment, research & evaluation 2005;10(7):1-9.

(25) De Vet HC, Terwee CB, Mokkink LB, Knol DL. Measurement in medicine: a practical guide. : Cambridge University Press; 2011.

(26) Field A. Discovering statistics using IBM SPSS statistics. : sage; 2013.

(27) Awang Z. SEM made simple: A gentle approach to learning Structural Equation Modeling. : MPWS Rich Publication; 2015.

(28) Hu L, Bentler PM. Cutoff criteria for fit indexes in covariance structure analysis: Conventional criteria versus new alternatives. Structural equation modeling: a multidisciplinary journal 1999;6(1):1-55.



For peer review only - http://bmjopen.bmj.com/site/about/guidelines.xhtml

(29) Dementia Australia. Your brain matters, the power of prevention. Available at: https://yourbrainmatters.org.au/. Accessed 15-2-, 2019.

(30) Edwards PJ, Roberts I, Clarke MJ, DiGuiseppi C, Wentz R, Kwan I, et al. Methods to increase response to postal and electronic questionnaires. The Cochrane Library 2009.

(31) McPeake J, Bateson M, O'Neill A. Electronic surveys: how to maximise success. Nurse Researcher (2014 ) 2014;21(3):24.

(32) Centraal Bureau voor de Statistiek. Bevolking; onderwijsniveau; geslacht, leeftijd en migratieachtergrond. 2019; Available at: https://statline.cbs.nl/StatWeb/publication/?VW=T&DM=SLNL&PA=82275NED&LA=NL. Accessed 20-2-, 2019.

For peer review only



For peer review only - http://bmjopen.bmj.com/site/about/guidelines.xhtml

Voornaam Achternaam
Straatnaam 12
1234AB Plaatsnaam
0612345678
demin@umcg.nl
ABCDE
Aangemeld op: 01-02-2020 10:01:16

01-02-2020 10:06:24

Beste Voornaam Achternaam,

Indien u wilt deelnemen aan dit onderzoek, vragen wij u dit toestemmingformulier door middel van een elektronische handtekening te ondertekenen. Hieronder vindt u de voorwaarde voor deelname aan dit onderzoek.

**Wanneer u het toestemmingsformulier ondertekent verklaart u dat:**
- u de informatiefolder met bijlagen en bovenstaande informatie heeft gelezen en hiermee voldoende bent geïnformeerd over het doel en de uitvoering van het onderzoek.
- U de mogelijkheid heeft gehad om aanvullende vragen te stellen (telefonisch of per mail), welke naar tevredenheid zijn beantwoord.
- u genoeg tijd had om te beslissen of u wilt deelnemen.
- u weet dat deelname vrijwillig is en dat u op ieder moment kan beslissen om toch niet mee te doen of te stoppen met het onderzoek.
- u weet dat u op de hoogte gesteld kan worden van medische relevante bevindingen.

**U geeft toestemming:**

– voor deelname aan het landelijk proef-bevolkingsonderzoek naar de beschermende en risicofactoren voor dementie (Demin studie).
– dat u in de toekomst opnieuw benaderd kan worden voor deelname aan aanvullend onderzoek.
– om uw onderzoeksgegevens te koppelen aan gegevens van het Centraal Bureau voor Statistiek (CBS), zoals uw gegevens over woonomgeving (bijvoorbeeld sportfaciliteiten).

Met vriendelijke groet,

Het Demin team
**www.demin.nl**

---------------------------------------------------------------------------------------------------------------
Ik verklaar dat ik deze proefpersoon volledig heb geïnformeerd over het genoemde onderzoek.

Als er tijdens het onderzoek informatie bekend wordt die de toestemming van de proefpersoon zou kunnen beïnvloeden, dan breng ik hem/haar daarvan tijdig op de hoogte.

Naam hoofdonderzoeker:
Handtekening: Datum:
Tijdstip:

*De deelnemer krijgt een volledige informatiebrief, samen met een kopie van het getekende toestemmingsformulier.*

For peer review only

# Reporting checklist for protocol of a clinical trial.

Based on the SPIRIT guidelines.

## Instructions to authors

Complete this checklist by entering the page numbers from your manuscript where readers will find each of the items listed below.

Your article may not currently address all the items on the checklist. Please modify your text to include the missing information. If you are certain that an item does not apply, please write "n/a" and provide a short explanation.

Upload your completed checklist as an extra file when you submit to a journal.

In your methods section, say that you used the SPIRITreporting guidelines, and cite them as:

Chan A-W, Tetzlaff JM, Altman DG, Laupacis A, Gøtzsche PC, Krleža-JeriĆ K, Hróbjartsson A, Mann H, Dickersin K, Berlin J, Doré C, Parulekar W, Summerskill W, Groves T, Schulz K, Sox H, Rockhold FW, Rennie D, Moher D. SPIRIT 2013 Statement: Defining standard protocol items for clinical trials. Ann Intern Med. 2013;158(3):200-207

| | | Reporting Item | Page Number |
|---|---|---|---|
| **Administrative information** | | | |
| Title | #1 | Descriptive title identifying the study design, population, interventions, and, if applicable, trial acronym | 1 |
| Trial registration | #2a | Trial identifier and registry name. If not yet registered, name of intended registry | 3 |
| Trial registration: data set | #2b | All items from the World Health Organization Trial Registration Data Set | All pages, no results |
| Protocol version | #3 | Date and version identifier | 1 |
| Funding | #4 | Sources and types of financial, material, and other support | 21 |
| Roles and responsibilities: contributorship | #5a | Names, affiliations, and roles of protocol contributors | 21 |
| Roles and responsibilities: sponsor contact information | #5b | Name and contact information for the trial sponsor | 1 |

For peer review only

| | | | |
|---|---|---|---|
| Roles and responsibilities: sponsor and funder | #5c | Role of study sponsor and funders, if any, in study design; collection, management, analysis, and interpretation of data; writing of the report; and the decision to submit the report for publication, including whether they will have ultimate authority over any of these activities | 23 |
| Roles and responsibilities: committees | #5d | Composition, roles, and responsibilities of the coordinating centre, steering committee, endpoint adjudication committee, data management team, and other individuals or groups overseeing the trial, if applicable (see Item 21a for data monitoring committee) | 23 |
| **Introduction** | | | |
| Background and rationale | #6a | Description of research question and justification for undertaking the trial, including summary of relevant studies (published and unpublished) examining benefits and harms for each intervention | 5-6 |
| Background and rationale: choice of comparators | #6b | Explanation for choice of comparators | 6 |
| Objectives | #7 | Specific objectives or hypotheses | 7 |
| Trial design | #8 | Description of trial design including type of trial (eg, parallel group, crossover, factorial, single group), allocation ratio, and framework (eg, superiority, equivalence, non-inferiority, exploratory) | 7-8 |
| **Methods: Participants, interventions, and outcomes** | | | |
| Study setting | #9 | Description of study settings (eg, community clinic, academic hospital) and list of countries where data will be collected. Reference to where list of study sites can be obtained | 7 |
| Eligibility criteria | #10 | Inclusion and exclusion criteria for participants. If applicable, eligibility criteria for study centres and individuals who will perform the interventions (eg, surgeons, psychotherapists) | 8-9 |
| Interventions: description | #11a | Interventions for each group with sufficient detail to allow replication, including how and when they will be administered | 9-16 |
| Interventions: modifications | #11b | Criteria for discontinuing or modifying allocated interventions for a given trial participant (eg, drug dose change in response to harms, participant request, or | n/a |

For peer review only

| | | | |
|---|---|---|---|
| | | improving / worsening disease) | |
| Interventions: adherance | #11c | Strategies to improve adherence to intervention protocols, and any procedures for monitoring adherence (eg, drug tablet return; laboratory tests) | 10 |
| Interventions: concomitant care | #11d | Relevant concomitant care and interventions that are permitted or prohibited during the trial | |
| Outcomes | #12 | Primary, secondary, and other outcomes, including the specific measurement variable (eg, systolic blood pressure), analysis metric (eg, change from baseline, final value, time to event), method of aggregation (eg, median, proportion), and time point for each outcome. Explanation of the clinical relevance of chosen efficacy and harm outcomes is strongly recommended | 16-20 |
| Participant timeline | #13 | Time schedule of enrolment, interventions (including any run-ins and washouts), assessments, and visits for participants. A schematic diagram is highly recommended (see Figure) | 11, 16 |
| Sample size | #14 | Estimated number of participants needed to achieve study objectives and how it was determined, including clinical and statistical assumptions supporting any sample size calculations | 9 |
| Recruitment | #15 | Strategies for achieving adequate participant enrolment to reach target sample size | 7 |
| **Methods: Assignment of interventions (for controlled trials)** | | | |
| Allocation: sequence generation | #16a | Method of generating the allocation sequence (eg, computer-generated random numbers), and list of any factors for stratification. To reduce predictability of a random sequence, details of any planned restriction (eg, blocking) should be provided in a separate document that is unavailable to those who enrol participants or assign interventions | 8-9 |
| Allocation concealment mechanism | #16b | Mechanism of implementing the allocation sequence (eg, central telephone; sequentially numbered, opaque, sealed envelopes), describing any steps to conceal the sequence until interventions are assigned | n/a |
| Allocation: implementation | #16c | Who will generate the allocation sequence, who will enrol participants, and who will assign participants to interventions | 8-9 |
| Blinding (masking) | #17a | Who will be blinded after assignment to interventions (eg, trial participants, care providers, outcome assessors, data analysts), and how | n/a, one recruitment strategy per |

For peer review only

| | | | |
|---|---|---|---|
| | | | memory clinic |
| Blinding (masking): emergency unblinding | #17b | If blinded, circumstances under which unblinding is permissible, and procedure for revealing a participant's allocated intervention during the trial | n/a |
| **Methods: Data collection, management, and analysis** | | | |
| Data collection plan | #18a | Plans for assessment and collection of outcome, baseline, and other trial data, including any related processes to promote data quality (eg, duplicate measurements, training of assessors) and a description of study instruments (eg, questionnaires, laboratory tests) along with their reliability and validity, if known. Reference to where data collection forms can be found, if not in the protocol | 11 |
| Data collection plan: retention | #18b | Plans to promote participant retention and complete follow-up, including list of any outcome data to be collected for participants who discontinue or deviate from intervention protocols | 10 |
| Data management | #19 | Plans for data entry, coding, security, and storage, including any related processes to promote data quality (eg, double data entry; range checks for data values). Reference to where details of data management procedures can be found, if not in the protocol | 10-11 |
| Statistics: outcomes | #20a | Statistical methods for analysing primary and secondary outcomes. Reference to where other details of the statistical analysis plan can be found, if not in the protocol | 19 |
| Statistics: additional analyses | #20b | Methods for any additional analyses (eg, subgroup and adjusted analyses) | n/a |
| Statistics: analysis population and missing data | #20c | Definition of analysis population relating to protocol non-adherence (eg, as randomised analysis), and any statistical methods to handle missing data (eg, multiple imputation) | n/a |
| **Methods: Monitoring** | | | |
| Data monitoring: formal committee | #21a | Composition of data monitoring committee (DMC); summary of its role and reporting structure; statement of whether it is independent from the sponsor and competing interests; and reference to where further details about its charter can be found, if not in the protocol. Alternatively, an explanation of why a DMC is not needed | 22 |

For peer review only

| | | | |
|---|---|---|---|
| Data monitoring: interim analysis | #21b | Description of any interim analyses and stopping guidelines, including who will have access to these interim results and make the final decision to terminate the trial | n/a, low risk |
| Harms | #22 | Plans for collecting, assessing, reporting, and managing solicited and spontaneously reported adverse events and other unintended effects of trial interventions or trial conduct | 20-21 |
| Auditing | #23 | Frequency and procedures for auditing trial conduct, if any, and whether the process will be independent from investigators and the sponsor | 16-17 |
| **Ethics and dissemination** | | | |
| Research ethics approval | #24 | Plans for seeking research ethics committee / institutional review board (REC / IRB) approval | 22 |
| Protocol amendments | #25 | Plans for communicating important protocol modifications (eg, changes to eligibility criteria, outcomes, analyses) to relevant parties (eg, investigators, REC / IRBs, trial participants, trial registries, journals, regulators) | n/a |
| Consent or assent | #26a | Who will obtain informed consent or assent from potential trial participants or authorised surrogates, and how (see Item 32) | 22 |
| Consent or assent: ancillary studies | #26b | Additional consent provisions for collection and use of participant data and biological specimens in ancillary studies, if applicable | n/a |
| Confidentiality | #27 | How personal information about potential and enrolled participants will be collected, shared, and maintained in order to protect confidentiality before, during, and after the trial | 21 |
| Declaration of interests | #28 | Financial and other competing interests for principal investigators for the overall trial and each study site | 22 |
| Data access | #29 | Statement of who will have access to the final trial dataset, and disclosure of contractual agreements that limit such access for investigators | 16, 22 |
| Ancillary and post trial care | #30 | Provisions, if any, for ancillary and post-trial care, and for compensation to those who suffer harm from trial participation | n/a, low risk |
| Dissemination policy: trial results | #31a | Plans for investigators and sponsor to communicate trial results to participants, healthcare professionals, the public, and other relevant groups (eg, via publication, reporting in results databases, or other data sharing arrangements), including any publication restrictions | 21 |

For peer review only

| | | | |
|---|---|---|---|
| Dissemination policy: authorship | #31b | Authorship eligibility guidelines and any intended use of professional writers | 21 |
| Dissemination policy: reproducible research | #31c | Plans, if any, for granting public access to the full protocol, participant-level dataset, and statistical code | 21 |
| **Appendices** | | | |
| Informed consent materials | #32 | Model consent form and other related documentation given to participants and authorised surrogates | 21 |
| Biological specimens | #33 | Plans for collection, laboratory evaluation, and storage of biological specimens for genetic or molecular analysis in the current trial and for future use in ancillary studies, if applicable | n/a |

None The SPIRIT checklist is distributed under the terms of the Creative Commons Attribution License CC-BY-ND 3.0. This checklist can be completed online using https://www.goodreports.org/, a tool made by the EQUATOR Network in collaboration with Penelope.ai

For peer review only

BMJ Open

# The uptake and effectiveness of a tailor-made online lifestyle program targeting modifiable risk factors for dementia among middle-aged descendants of people with recently diagnosed dementia: study protocol of a cluster randomised controlled trial (Demin study)



| | |
|---|---|
| Journal: | *BMJ Open* |
| Manuscript ID | bmjopen-2020-039439.R1 |
| Article Type: | Protocol |
| Date Submitted by the Author: | 07-Jul-2020 |
| Complete List of Authors: | Vrijsen, Joyce; University of Groningen, University Medical Centre Groningen, Epidemiology<br>Abu-Hanna, Ameen; University of Amsterdam, Amsterdam UMC, Medical Informatics<br>Maeckelberghe, Els; University of Groningen, University Medical Centre Groningen, Wenckebach Institute for Training and Education<br>De Deyn, Peter Paul; University of Groningen, University Medical Centre Groningen, Neurology and Alzheimer Centre Groningen<br>de Winter, Andrea; University of Groningen, University Medical Centre Groningen, Health Sciences<br>Reesink, Fransje; University of Groningen, University Medical Centre Groningen, Neurology and Alzheimer Centre Groningen<br>Oude Voshaar, Richard; University of Groningen, University Medical Center Groningen, Psychiatry<br>Buskens, Erik; University of Groningen, University Medical Centre Groningen, Epidemiology<br>de Rooij, Sophia; Medical Spectrum Twente, Medical School<br>Smidt, Nynke; University of Groningen, University Medical Centre Groningen, Epidemiology |
| <b>Primary Subject Heading</b>: | Public health |
| Secondary Subject Heading: | Epidemiology, Public health |
| Keywords: | Dementia < NEUROLOGY, Risk management < HEALTH SERVICES ADMINISTRATION & MANAGEMENT, EPIDEMIOLOGY, PREVENTIVE MEDICINE, PUBLIC HEALTH |
| | |



SCHOLARONE™
Manuscripts

*I, the Submitting Author has the right to grant and does grant on behalf of all authors of the Work (as defined in the below author licence), an exclusive licence and/or a non-exclusive licence for contributions from authors who are: i) UK Crown employees; ii) where BMJ has agreed a CC-BY licence shall apply, and/or iii) in accordance with the terms applicable for US Federal Government officers or employees acting as part of their official duties; on a worldwide, perpetual, irrevocable, royalty-free basis to BMJ Publishing Group Ltd ("BMJ") its licensees and where the relevant Journal is co-owned by BMJ to the co-owners of the Journal, to publish the Work in this journal and any other BMJ products and to exploit all rights, as set out in our licence.*

*The Submitting Author accepts and understands that any supply made under these terms is made by BMJ to the Submitting Author unless you are acting as an employee on behalf of your employer or a postgraduate student of an affiliated institution which is paying any applicable article publishing charge ("APC") for Open Access articles. Where the Submitting Author wishes to make the Work available on an Open Access basis (and intends to pay the relevant APC), the terms of reuse of such Open Access shall be governed by a Creative Commons licence – details of these licences and which Creative Commons licence will apply to this Work are set out in our licence referred to above.*

*Other than as permitted in any relevant BMJ Author's Self Archiving Policies, I confirm this Work has not been accepted for publication elsewhere, is not being considered for publication elsewhere and does not duplicate material already published. I confirm all authors consent to publication of this Work and authorise the granting of this licence.*

For peer review only

**TITLE (WORD COUNT: 4427)**

# The uptake and effectiveness of a tailor-made online lifestyle program targeting modifiable risk factors for dementia among middle-aged descendants of people with recently diagnosed dementia: study protocol of a cluster randomised controlled trial (Demin study)

**Issue date:** 7 July 2020

**AUTHORS**

Vrijsen J[1]*, Abu-Hanna A[2], Maeckelberghe ELM[3], De Deyn PP[4], de Winter AF[5], Reesink FE[4], Oude Voshaar RC[6], Buskens E[1], de Rooij SEJA[7], Smidt N[1], for the Demin consortium.

*Corresponding author. University Medical Centre Groningen, Department of Epidemiology, Hanzeplein 1, PO Box 30 001, FA40, 9700 RB Groningen, The Netherlands. E-mail: j.vrijsen@umcg.nl; Phone: +31625650782

**Author affiliations**

1 University of Groningen, University Medical Centre Groningen, Department of Epidemiology, Groningen, the Netherlands

2 University of Amsterdam, Amsterdam UMC, Department of Medical Informatics, Amsterdam, the Netherlands

3 University of Groningen, University Medical Centre Groningen, Wenckebach Institute for Training and Education, Groningen, the Netherlands

4 University of Groningen, University Medical Centre Groningen, Department of Neurology and Alzheimer Centre Groningen, Groningen, the Netherlands

5 University of Groningen, University Medical Centre Groningen, Department of Health Sciences, Groningen, the Netherlands

6 University of Groningen, University Medical Centre Groningen, Department of Psychiatry, Groningen, the Netherlands

7 Medical Spectrum Twente, Medical School, Enschede, the Netherlands



For peer review only - http://bmjopen.bmj.com/site/about/guidelines.xhtml

**Demin consortium (28)**

Abma EM, Abu-Hanna A, Buskens E, Claassen JAHR, De Deyn PP, Feitsma T , Hempenius L, Hoogmoed J, Kamper A, Koek HL, Kragt JJ, Lagro J, Lambooij SLE, Langedijk M, Maeckelberghe ELM, Mattace Raso FUS, Meinardi MC, Oude Voshaar RC, Reesink FE, de Rooij SEJA, Scheepmaker AJTM, Smidt N, Spies P, Taekema D, Verkuyl J, Vingerhoets R, Vrijsen J, de Winter AF.

For peer review only



For peer review only - http://bmjopen.bmj.com/site/about/guidelines.xhtml

## ABSTRACT

**Introduction** Descendants of dementia patients have a higher risk to develop dementia. This study aims to investigate the uptake and effectiveness of an online tailor-made lifestyle program for Dementia Risk Reduction (DRR) among middle-aged descendants of people with recently diagnosed late-onset dementia.

**Methods and analysis** Demin is a cluster randomised controlled trial, aiming to include 21 memory clinics of which thirteen will be randomly allocated to the passive (poster and flyer in waiting room) and eight to the active recruitment strategy (additional personal invitation by members of the team of the memory clinic). We aim to recruit 378 participants (40-60 years) with a parent who is recently diagnosed with Alzheimer's Disease or Vascular Dementia at one of the participating memory clinics. All participants receive a dementia risk assessment (online questionnaire, physical examination and blood sample) and subsequently an online tailor-made lifestyle advice regarding protective (Mediterranean diet, low/moderate alcohol consumption, high cognitive activity) and risk factors (physical inactivity, smoking, loneliness, cardiovascular disease, hypertension, high cholesterol, diabetes, obesity, renal dysfunction, depression) for dementia. The primary outcome is the difference in uptake between the two recruitment strategies. Secondary outcomes are change(s) in 1) the Lifestyle for Brain Health (LIBRA) score, 2) individual health behaviours, 3) health beliefs and attitudes towards DRR and 4) compliance to the tailor-made lifestyle advice. Outcomes will be measured at 3, 6, 9 and 12 months after baseline. The effectiveness of this online tailor-made lifestyle program will be evaluated by comparing Demin participants to a matched control group (Lifelines cohort).

**Ethics and dissemination** This study has been approved by the Dutch Ministry of Health, Welfare and Sport according to the Population Screening Act. All participants have to give online informed consent using SMS-tan. Findings will be disseminated through peer-reviewed journals and (inter)national conferences.

**Trial registration number** NTR7434




For peer review only - http://bmjopen.bmj.com/site/about/guidelines.xhtml

## ARTICLE SUMMARY

### Strengths and limitations of this study

- This is the first multicentre trial that focuses on dementia risk reduction in middle-aged descendants of recently diagnosed patients with Alzheimer's disease or Vascular dementia.
- The program gives participants insight in their risk and protective factors for dementia and provides a tailor-made online lifestyle advice with regard to thirteen modifiable risk factors for dementia, taking the stages of (health behaviour) change into account.
- The application ensures the privacy of the participants by using SMS-tan for logging in their personal account and signing the electronic informed consent form.
- The web-based application (demin.nl) functions fully automatically, making it easy to implement the study in other memory clinics and settings.
- Changing health behaviour is difficult and it is unclear whether a tailor-made online lifestyle advice is sufficient to change health behaviour and to maintain a healthy lifestyle.

**KEY WORDS**

Dementia

Health behaviour

Risk reduction behaviour

Lifestyle

Middle aged



For peer review only - http://bmjopen.bmj.com/site/about/guidelines.xhtml

For peer review only

## INTRODUCTION

Dementia is considered a major public health concern [1]. Due to the ageing population the number of dementia cases will increase substantially in the next decades. In 2015, more than 46 million people worldwide were affected by dementia and this number is expected to increase to 131 million by 2050 [2]. This rise in people with dementia carries a high economic and social burden for society [1]. In 2015, global costs of dementia reached 818 billion US dollars and will increase further [3]. Currently, no curative treatments are available. Therefore, prevention is a key element to counteract the dementia epidemic [4,5].

The most common types of dementia are Alzheimer's disease (AD) (60-70%) ,Vascular dementia (VD) (15-20%) or a combination of AD and VD (mixed dementia) [6–8]. The presence of a first-degree relative with AD doubles the risk for developing AD [9]. This increased risk has several reasons. Firstly, descendants of people with AD more often have a higher genetic predisposition for AD (e.g. carrier of the Apo lipoprotein E (APOE) ε4 allele) [9]. Secondly, high blood pressure, vascular diseases and other vascular risk factors (i.e. diabetes type 2, obesity, hypercholesterolemia) often cluster in families [10]. Lastly, psychosocial behaviour runs in the family and also affects health behaviour and lifestyle [11,12]. Not surprisingly, individuals with a parent who is recently diagnosed with AD or VD often worry about their own risk of developing dementia. Therefore, this life event (parental diagnosis of dementia) might encourage the willingness of individuals to change their health behaviour [13].

Parental family history has been associated with an increased risk of dementia independently of known genetic risk factors [9,14]. Therefore, a healthy lifestyle might be beneficial for individuals with a positive family history. Over the last decade, evidence of modifiable risk factors for dementia has been mounting [4,6,15]. The Lancet commission on dementia prevention, intervention and care demonstrated that 35% of the dementia cases is attributable to modifiable risk factors (i.e. less education, hearing loss, midlife hypertension, midlife obesity, smoking, depression, physical inactivity, social isolation and diabetes) and recommended to start interventions including more



For peer review only - http://bmjopen.bmj.com/site/about/guidelines.xhtml

childhood education, promotion of physical exercise, reduction of smoking, maintaining social engagement and management of hypertension, diabetes, obesity, depression and hearing loss [4,6,16]. Other major risk factors are hyperlipidaemia, coronary heart disease, renal dysfunction, Mediterranean diet and cognitive activity [15].

Only few studies examined the effectiveness of targeting these modifiable factors on cognitive decline and dementia incidence through a multi-domain intervention, such as the (Finnish Geriatric Intervention Study to Prevent Cognitive Impairment and Disability) FINGER study [17], the (Prevention of Dementia by Intensive Vascular care) PreDIVA study [18] and the (The Multi-domain Alzheimer Preventive Trial) MAPT study [19]. These studies, with a follow-up varying from two to six years, found small or non-significant effects on cognition in older participants (e.g. >60 years) [17–19]. Starting multi-domain interventions earlier in life might be promising as cognitive decline begins already in midlife [20,21]. However, since dementia is mainly prevalent in the elderly, a long follow-up period of approximately 20 years would be required in order to determine the effectiveness of interventions on dementia incidence [20–22]. Furthermore, tailoring interventions improves the effectiveness of health behaviour change interventions [23]. Web-based interventions have the potential to support health behaviour change as there is the opportunity to tailor lifestyle advice [24–27]. They were especially effective when a theoretical basis or conceptual framework (e.g. Health belief model (HBM), Trans theoretical model (TTM), Theory of planned behaviour (TPB), I(integrated)-Change model [28–32]), behaviour change techniques (e.g. providing feedback on performance and information on the consequences of unhealthy behaviour) and several modes of delivery had been used [33].

The first challenge of health behaviour change interventions is to achieve a high level of uptake for screening (e.g. assessing risk and protective factors for dementia), reflecting the willingness to participate. A systematic review identified a large variation in uptake in health checks and lifestyle intervention programs [34], depending on the type of recruitment strategy. The two main types of strategies for recruitment are the active and passive recruitment strategy. Active recruitment involves a

For peer review only



For peer review only - http://bmjopen.bmj.com/site/about/guidelines.xhtml

personal invitation by the project staff and healthcare providers (e.g. proactive) and passive recruitment involves recruitment of participants through various channels such as flyers and advertisements (e.g. reactive) [35]. The most effective recruitment strategy is proactive referral from a healthcare provider, while displaying posters and flyers showed to be less effective [36]. Uptake also depends on other factors as described in social cognition models (e.g. knowledge, perceived susceptibility and severity, facilitators, benefits and barriers, and attitude towards such interventions) [28–32]. These factors are essential and useful to make a well-informed decision about dementia risk assessment, considering the possible benefits and harms. Therefore, information on dementia, the risk and protective factors for dementia, heritability, and how to tackle risk and protective factors for dementia are important factors in the development of a web-based intervention. A previous study showed that the majority of the Dutch general population is unaware of the relationship between modifiable risk factors and brain health, particularly regarding major cardiovascular risk factors (e.g. hypertension, hypercholesterolemia and coronary heart disease) [37]. Having a parent who is recently diagnosed with AD or VD could have led to an increased knowledge on dementia and risk perception [13]. Therefore, middle-aged descendants of recently diagnosed people with AD or VD might be receptive to assess their risk and motivated to adopt a healthier lifestyle as they just realized their (familial) risk [13,38].

To our knowledge, none of the health behaviour intervention studies were aimed at a specific group of middle-aged adults with increased risk for dementia due to their parental family history of dementia. Therefore, this study aims to investigate the uptake and effectiveness of a tailor-made online lifestyle program for dementia risk reduction among middle-aged descendants of recently diagnosed (in the last six months) people with AD or VD in the Netherlands. This will give insight in to what extent it is feasible to recruit middle-aged descendants of people with AD or VD at the memory clinics and whether these potential participants are willing to participate in a tailor-made online lifestyle program in order to reduce their dementia risk,

## METHODS AND ANALYSIS



For peer review only - http://bmjopen.bmj.com/site/about/guidelines.xhtml

**Study setting and design**

This study is a pragmatic cluster randomized controlled trial (RCT), including 21 participating memory clinics in the Netherlands who are randomly allocated to a passive or active recruitment of participants. Memory clinics allocated to the active recruitment strategy invite potential participants face-to-face by a member of the team of the memory clinic to participate in the tailor-made online lifestyle program for dementia risk reduction (also called the Demin study), next to posters and flyers that are placed in the waiting room of the memory clinic. Memory clinics allocated to the passive recruitment strategy, do not invite potential participants pro-actively, but invite potential participants to participate in the Demin study by posters and flyers that are placed in the waiting room of the memory clinic.

Patients with AD or VD (or their caregivers) receive an envelope either at the registration desk of the memory clinic or after the consult of the patient (only with active recruitment). This envelope is addressed to the middle-aged descendants of patients with recently diagnosed AD or VD and includes a patient information form (PIF) with information about the content of the study, the advantages and disadvantages of study participation and how potential participants can participate. Potential participants (one family member per patient) are asked to register themselves (e.g. making an account) on the Demin website (www.demin.nl), by using the memory clinic specific login access code, which is reported on the front page of the PIF and represents the memory clinic in which the parent was diagnosed. The decision to participate is confirmed by the participants by signing the online informed consent form (electronic signature by using SMS-tan). After signing this form, individuals from both recruitment strategies are able to log in to their personalized website 'My Demin' and continue the intervention in an equal manner. The personalized website 'My Demin' is secured and only accessible for the participant by logging in with their personal e-mail address, password and SMS-tan code. 'My Demin' contains the following information:  1) My personal (account) information, 2) Message inbox, 3) My online questionnaires, 4) My personal health profile including online tailor-made lifestyle advice. After participants have completed the online questionnaire, they automatically receive a message with a request to make an appointment for physical examination including a fasting blood sample. Moreover, participants can invite siblings to participate in the study in 'My Demin'.



For peer review only - http://bmjopen.bmj.com/site/about/guidelines.xhtml

The functionalities provided by the Demin website are based on the literature and input we received from people with a parent with dementia (focus group discussions).

**Randomization of memory clinics**

To prevent contamination between the two recruitment strategies, randomization is performed at the level of the memory clinics. To enhance comparability between the intervention (participants of the active recruitment strategy) and control group (participants of the passive recruitment strategy), the memory clinics will be matched and randomised by a statistician, who is blind to the identity of the memory clinics and not involved in the study. Firstly, all participating memory clinics will be matched into pairs based on the following criteria: (i) number of newly diagnosed dementia (VD, AD or mixed dementia) patients seen per year (range vary from 60 to 350 patients per year) and (ii) the average social economic position (SEP) of the population living around the memory clinic (neighbourhood SEP), based on data from Statistics Netherlands [39]. Secondly, the matched memory clinics will be randomized (pairwise randomization) to an active recruitment strategy or passive recruitment strategy using a computer-generated random number list. As we expect a higher response rate in the active recruitment strategy group, we use an active : passive recruitment strategy ratio of 8:13 (see sample size calculations).

**Study population**

Eligible participants are middle-aged individuals (40-60 years old) with a parent who is recently (less than 6 months ago) diagnosed with AD or VD (or mixed dementia) at one of the participating memory clinics in the Netherlands (see acknowledgement). Individuals should provide informed consent, be able to fill out an online Dutch questionnaire. Pregnant women are excluded from participation.

**Sample size calculations**

The primary outcome measure is uptake, which is defined as the percentage of eligible individuals that signed the online informed consent form and completed baseline assessment (online questionnaire and physical examination and a fasting blood sample). In order to detect a difference of 20% in uptake

For peer review only

between the passive and active recruitment strategy (30% versus 50%), we need 94 participants in each group to achieve a power of 80% with alpha levels of 0.05 (total = 188 participants). To take cluster randomization into account, we use the formula 1+ ((n-1)*ICC) (inflation factor), where n is the average number of included participants per memory clinic and the ICC the Intra Class Correlation [40]. The ICC is unknown, but an ICC of 0.05 is a common value for cluster randomized controlled trials in hospitals [41]. The estimated average of included participants per memory clinic per year is n=15 using a passive recruitment strategy and n=25 using an active recruitment strategy, taking into account non-response. With unequal cluster sizes, 'n' is replaced by 'm', where m is the sum of $(M)^2/\text{sum}(M)$ $((15^2+25^2) / (15+25))$ [42]. This results in a sample size inflation factor of (1+ ((21.25-1)*0.05)= 2.01. Therefore, the total number of participants needed is 378 (2.01 * 188). In order to recruit 378 participants, we need 21 memory clinics, of which eight memory clinics (responsible for 189 included participants) will be allocated to the active recruitment strategy and thirteen memory clinics (responsible for 189 included participants) will be allocated to the passive recruitment strategy.

**Demin website**

The Demin website is available for everyone and provides information about dementia, heredity of dementia, risk and protective factors for dementia, and how to tackle potential risk factors for dementia. The health information will be provided by written text and in an audio-visual format, such as a spoken animation, to assure inclusion of participants with different levels of health literacy. [43].. According to the cognitive theory of multimedia learning (CTML), people process visual and auditory information through different channels [44,45]. It is known that health information provided by various channels, such as written text and spoken animations, improves information processing compared to information only provided through written text or spoken animations [44,45]. The instructions for registration (making an account, signing informed consent) are also provided as written text as visual screenshots representing the steps of the registration process.

**Online tailor-made lifestyle program for dementia risk reduction**

For peer review only



For peer review only - http://bmjopen.bmj.com/site/about/guidelines.xhtml

After participants give online informed consent, participants have access to the online tailor-made lifestyle program for dementia risk reduction, which consists of 1) a dementia risk assessment and 2) an online tailor-made lifestyle advice including a personal health profile targeting risk and protective factors for dementia.

**1. Dementia risk assessment**

The dementia risk assessment consists of filling out an online questionnaire (in 'My Demin') and physical examination, including a fasting blood sample, at one of the 21 participating memory clinics in order to determine whether risk and protective factors are present. In order to minimize the amount of missing data, validation and skip-and-fail rules were implemented in the online questionnaire. Furthermore, automatic reminders are sent to the participant if the online questionnaire was not filled in within two weeks. Physical examination will be conducted by the team of the local memory clinic and includes the following measurements: height (in cm) (SECA 222 stadiometer), body weight (in kg) without shoes (SECA 761 scale), waist- and hip circumference (in cm) (SECA 200 measuring tape), and three measurements of diastolic and systolic blood pressure (in mmHg) (Welch Allyn 'Spot Vital Signs' [46]). After physical examination, which takes approximately 15 minutes, a fasting blood sample (maximum of 21 ml) is taken for direct laboratory measurement of glucose, HbA1C, total cholesterol, High-density-lipoprotein (HDL), Low-density-lipoprotein (LDL), triglycerides and serum creatinine. The results of the physical examination (height, body weight, blood pressure, waist- and hip circumference) are sent to the researcher (J. Vrijsen) to check the entry of the results by the participants. The results of the direct laboratory measurements are sent to the medical doctor (E.M. Abma) of the University Medical Centre Groningen to check for deviating values.

**Risk and protective factors for dementia**

Through the online questionnaire and physical examination, data on thirteen currently known protective (i.e. Mediterranean diet, low/moderate alcohol consumption, cognitive activity) and risk factors (i.e. physical inactivity, smoking, loneliness, cardiovascular diseases, hypertension, high cholesterol, diabetes mellitus, obesity, renal dysfunction, depression) for dementia are collected



For peer review only - http://bmjopen.bmj.com/site/about/guidelines.xhtml

[6,15,47]. See **Table 1** for an overview of the assessment measures. The measurements of these risk and protective factors are described in **Supplementary file 1**.

**Table 1.** Assessment measures at baseline and follow up

| | **Baseline** | **3 months** | **6 months** | **9 months** | **12months** |
|---|---|---|---|---|---|
| **RISK AND PROTECTIVE FACTORS** | | | | | |
| Smoking | Q | Q | Q | Q | Q |
| Physical inactivity (SQUASH, IPAQ) | Q | Q | Q | Q | Q |
| Mediterranean diet (FFQ) | Q | Q | Q | Q | Q |
| Alcohol consumption (FFQ) | Q | Q | Q | Q | Q |
| High cognitive activity (CRIq) | Q | Q | Q | Q | Q |
| Loneliness (de Jong Gierveld, 6-item) | Q | Q | Q | Q | Q |
| Cardiovascular diseases (CVD) | Q | Q | Q | Q | Q |
| Obesity (body weight, height) | Q+ PE | Q | Q | Q | Q+PE |
| Hypertension (SBD, DBP) | Q+PE | Q | Q | Q | Q+PE |
| High cholesterol (LDL, HDL, TC) | Q+FBS | Q | Q | Q | Q+FBS |
| Diabetes Mellitus (glucose, HbA1C) | Q+FBS | Q | Q | Q | Q+FBS |
| Renal dysfunction (eGFR) | Q+FBS | Q | Q | Q | Q+FBS |
| Depression (CES-D) | Q | Q | Q | Q | Q |

*SQUASH* Short Questionnaire to Assess Health-enhancing physical activity, *IPAQ* International Physical Activity Questionnaire, *FFQ* Food Frequency Questionnaire, *CRIq* Cognitive Reserve Index questionnaire (adapted), *CVD* Cardiovascular diseases, *SBP* Systolic Blood Pressure, *DBP* Diastolic Blood Pressure, *HDL* high-density lipoproteins, *LDL* low-density lipoproteins, *TC* total cholesterol, *HbA1C* Haemoglobin A1C, *eGFR* estimated Glomerular Filtration Rate, *CES-D* Centre for Epidemiological Studies Depression Scale

Q: Online questionnaire, PE: Physical examination, FBS: Fasting blood sample

**2a. Personal health profile**

For peer review only



For peer review only - http://bmjopen.bmj.com/site/about/guidelines.xhtml

After completion of the baseline dementia risk assessment (including the data entry of the physical examination and laboratory measurements), a personal health profile is automatically provided in the personal account of the participants (My Demin). The personal health profile gives an overview of the presence of the risk and protective factors for dementia. According to the Lifestyle for Brain Health (LIBRA) score, each risk and protective factor [47–49] is categorized into one of the following categories: 1) room for improvement, 2) remember to manage well, 3) keep this up (see **Table 2**). The "Keep this up" category represent factors that participants are currently managing well or diseases they do not have. The "Room for improvement" category represents the factors that could be improved by health behaviour change (e.g. quit smoking, become more physical active, change diet, drink less alcohol). The category "Remember to manage well" is assigned when a risk factor (i.e. cardiovascular disease, hypertension, high cholesterol, diabetes mellitus, renal dysfunction and depression) is present, but the disease is managed well as participants have regular meetings with their general practitioner for disease control (diabetes mellitus) or use medication for disease management (cardiovascular disease, hypertension, high cholesterol, renal dysfunction and depression) (see **Figure 1**).

**[INSERT FIGURE 1 ABOUT HERE]**



For peer review only - http://bmjopen.bmj.com/site/about/guidelines.xhtml

**Table 2.** Definition for the 3 categories in the personal health profile at baseline

| Modifiable risk factors | Keep this up | Remember to manage well | Room for improvement |
|---|---|---|---|
| **Diet** | MIND-diet score = 14 points | n.a. | MIND-diet score < 14 points |
| **Alcohol consumption** | Average number of units of alcohol per week ≤ 7 and number of units per day is: ≤ 3 for women or ≤ 4 for men | n.a. | Average number of units of alcohol per week > 7 or number of units per day is: > 3 for women or > 4 for men |
| **Cognitive activity** | paid working hours ≥ 24 or CRIq score ≥ 50 | n.a. | paid working hours < 24 and CRIq score < 50 |
| **Physical activity** | (MVPA / week ≥ 150 and Sitting time ≤ 8 hours / day) or (MVPA / week < 150 and sitting time < 4 hours / day) | n.a. | (Sitting time > 8 hours / day) or (Sitting time ≥ 4 hours / day and MVPA / week < 150) |
| **Smoking** | Past or never smoker | n.a. | Current smoker |
| **Loneliness** | De Jong Gierveld score < 2 | n.a. | De Jong Gierveld score ≥ 2 |
| **Cardiovascular diseases (CVD)** | no CVD | at least one CVD and receives medical treatment | at least one CVD and no medical treatment |
| **Weight** | BMI ≥ 18.5 and BMI < 25.0 | n.a. | BMI < 18.5 or BMI ≥ 25.0 |



For peer review only - http://bmjopen.bmj.com/site/about/guidelines.xhtml

| | | | |
|---|---|---|---|
| **Blood pressure** | DBP < 90 mmHg and SBP < 140 and no medical treatment | DBP < 90 mmHg and SBP < 140 and medical treatment | DBP ≥ 90 mmHg or SBP ≥ 140 mmHg |
| **Cholesterol** | (LDL ≤ 2.5 mmol/l and TC/HDL ≤ 8) and no medical treatment | (LDL ≤ 2.5 mmol/l and TC/HDL ≤ 8) and medical treatment | LDL > 2.5 mmol/l or TC/HDL > 8 |
| **Diabetes Mellitus** | glucose < 7.0 mmol and HbA1C ≤ 53 mmol/mol | (HbA1C ≤ 53 mmol/mol and medical treatment) or (glucose < 7.0 mmol and HbA1C > 53 mmol/mol and medical treatment) | (HbA1C > 53 mmol/mol and no medical treatment) or (glucose ≥ 7.0 mmol and HbA1C > 53 mmol/mol) or (glucose ≥ 7.0 mmol and HbA1C ≤ 53 mmol/mol and no medical treatment) |
| **Kidney** | eGFR ≥ 60 ml/min/1.73 $m^2$ | eGFR < 60 ml/min/1.73 $m^2$ and medical treatment | eGFR < 60 ml/min/1.73 $m^2$ and no medical treatment |
| **Depression** | CES-D < 16 points | CES-D ≥ 16 points and medical treatment | CES-D ≥ 16 points and no medical treatment |

*MIND-diet* Mediterranean-DASH Diet Intervention for Neurodegenerative Delay, *CRIq* Cognitive Reserve Index questionnaire, *MVPA* Moderate to vigorous physical activity, CVD Cardiovascular diseases, *BMI* Body mass index, *DBP* diastolic blood pressure, *SBP* systolic blood pressure, *LDL* low-



For peer review only - http://bmjopen.bmj.com/site/about/guidelines.xhtml

density lipoproteins, *TC* total cholesterol, *HDL* high-density lipoproteins, *HbA1C* Haemoglobin A1C, *eGFR* estimated Glomerular Filtration Rate, *CES-D* Centre for Epidemiological Studies Depression Scale

For peer review only



For peer review only - http://bmjopen.bmj.com/site/about/guidelines.xhtml

**2b. Tailor-made online lifestyle advice for dementia risk reduction**

Participants also receive an online tailor-made lifestyle advice targeting risk factors associated with dementia and following the Dutch guidelines for a healthy diet, alcohol consumption, physical activity, diabetes mellitus, renal dysfunction and cardiovascular health including cholesterol levels and BMI [50–54]. For each risk and protective factor, information is given about (i) the norm (cut-off point for not having this risk factor), (ii) the association between the risk factor and dementia and (iii) lifestyle advice how to tackle this factor. The online lifestyle advice was tailored to the participants based on (i) the presence of risk factors, (ii) the strength of the association between the risk factors and dementia [15,47] and (iii) the stages of change of the health behaviour related risk factors (physical inactivity, diet, alcohol consumption, smoking behaviour, cognitive activity, social activity). The stages of change are determined by asking "Which statement fits best for you?", where each answer option reflects one of the following stages of change: pre-contemplation, contemplation, preparation, action and maintenance [29]. It is known that participants who are in the preparation and action stage are more willing to change their health behaviour, therefore lifestyle advice for these factors are given first [29].

In case medically relevant findings are found, including untreated diabetes mellitus (glucose $\geq$ 7.0 mmol/l or (glucose $\geq$ 6.1 mmol/l and HbA1C > 53 mmol/mol)), untreated renal dysfunction (estimated Glomerular Filtration Rate (eGFR) $\leq$ 60 ml/min/1.73 m2) and increased risk for developing cardiovascular diseases (CVD) (CVD risk $\geq$ 10% according to the Dutch SCORE formula [54]), participants receive, in addition to the online tailor-made lifestyle advice, a separate message in their personal inbox with the recommendation to contact their general practitioner to verify the results and discuss whether treatment is needed.

**Outcome measures and measurements**

Participants are invited to fill in the online questionnaire at baseline and four times (3, 6, 9 and 12 months after baseline measurement) during one year follow-up. Physical examination, including the fasting blood sample for direct laboratory measurements, is only done at baseline and 12 months after



For peer review only - http://bmjopen.bmj.com/site/about/guidelines.xhtml

329 baseline measurement (see **Supplementary file 2**). Data from the online questionnaires and physical
330 examination are stored automatically in an electronic Case Report Form (eCRF) data management
331 program, which is only accessible by the researchers involved in this study. Data from the direct
332 laboratory measurement are entered manually in the electronic Case Report Form (eCRF) data
333 management program. Every month, memory clinics are requested to provide information about 1) the
334 number of eligible participants (e.g. new cases of AD and VD), 2) the number of envelopes that are
335 given away, and 3) any difficulties with the recruitment of participants. In order to keep participating
336 memory clinics involved in the study, every three months newsletters are sent around and memory
337 clinics are contacted monthly to evaluate the uptake.

338

339 **Primary outcome**

340 The primary outcome is the difference in uptake (e.g. the percentage of eligible people that signed the
341 online informed consent form and completed risk assessment of the total number of eligible people)
342 between the active and passive recruitment strategy. The total number of eligible people in each
343 recruitment group (active versus passive) are based on the number of new cases of AD or VD in all
344 memory clinics during the recruitment period, assuming an average of one child per dementia patient
345 receiving the envelope with the PIF including a login access number.

346

347 **Secondary outcomes**

348 Secondary outcomes include:

349 **1)** The change in Lifestyle for Brain Health (LIBRA) score. The LIBRA score has been validated
350 among individuals in midlife and reflects an individual's potential to reduce their risk on developing
351 late-onset dementia [47]. The LIBRA score consists of twelve currently known protective (i.e.
352 Mediterranean diet, low/moderate alcohol consumption, cognitive activity) and risk factors (i.e.
353 physical inactivity, smoking, cardiovascular diseases, hypertension, high cholesterol, diabetes
354 mellitus, obesity, renal dysfunction, depression) for dementia (13, 14,31) and ranges from -5.9 (low
355 risk for developing dementia) to 12.7 (high risk for developing dementia).

For peer review only

A one point increase in the LIBRA score is associated with a 19% higher risk for dementia [47,55]. The definitions and corresponding scores for the three protective and ten risk factors for dementia are described in **Table 3**.

**Table 3.** Definition of risk and protective factors for dementia in the LIBRA score and corresponding scores

| Modifiable risk factors | | **Definition** | **Score** |
|---|---|---|---|
| **Protective factors** | | | |
| **1** | High cognitive activity | Score ≥ 50 points on the Cognitive Reserve Index questionnaire (leisure time activities) (CRIq) or hours of paid work ≥ 24 hours | -3.2 |
| **2** | Mediterranean diet | MIND-diet score (0-14) = 14 points | -1.7 |
| **3** | Low/moderate alcohol consumption | Average number of glasses of alcohol a week ≤ 7 and number of glasses a day is:<br>≤ 3 glasses for women (no binge drinking)<br>≤ 4 glasses for men (no binge drinking) | -1.0 |
| **Risk factors** | | | |
| **4** | Cardiovascular diseases (CVD) | Presence of at least one of the follow diseases: history of angina pectoris, myocardial infarction, transient ischemic attacks, stroke or peripheral arterial diseases | +1.0 |
| **5** | Physical inactivity | Not fulfilling Dutch Norm for Physical activity defined as ≥ 150 min/week physical activity of moderate to vigorous intensity, measured with the SQUASH questionnaire | +1.1 |
| **6** | Renal dysfunction | Estimated glomerular filtration rate ≤ 60 ml/min/1.73 | +1.1 |
| **7** | Diabetes Mellitus | Glucose (capillary blood) > 7.0 mmol/l or HbA1c > 53 mmol/mol | +1.3 |
| **8** | High cholesterol | LDL > 2.5 mmol/l or TC/HDL ≥ 8 | +1.4 |
| **9** | Smoking | Current smoker | +1.5 |



For peer review only - http://bmjopen.bmj.com/site/about/guidelines.xhtml

| | | | |
|---|---|---|---|
| **10** | Obesity | BMI ≥ 30 | +1.6 |
| **11** | Hypertension | SBP > 140 mmHg or DBP > 90 mmHg | +1.6 |
| **12** | Depression | Score ≥ 16 points on the Centre for Epidemiologic Studies Depression scale (CES-D) | +2.1 |

*LDL* low-density lipoproteins, *TC* total cholesterol, *HDL* high-density lipoproteins, *BMI* Body mass index, *SBP* systolic blood pressure, *DBP* diastolic blood pressure

**2)** The change in the individual health behaviours, including physical activity (minutes of MVPA per week), diet (MIND-diet score; 0-14), alcohol consumption (number of glasses of alcohol per week), smoking behaviour (current smoker (yes/no) and number of cigarettes/cigars a day), cognitive activity (leisure-time cognitive activity score and number of hours paid work), loneliness (overall loneliness score; 0-6) and social activity (number of contacts per two weeks) and their stage of change over time. The stages of change are categorized into pre-contemplation (1), contemplation (2), preparation (3), action (4) and maintenance (5) [29].

**3)** Changes in beliefs and attitudes with regard to dementia risk reduction are measured using the Motivation to Change Lifestyle and Health Behaviour for Dementia Risk Reduction Scale (MCLHB-DRR scale) [56,57]. The MCLHB-DRR scale is based on the Health Belief Model [28], which explains health-related behaviours. Seven subscales of the Health Belief Model were included: perceived susceptibility, perceived severity, perceived benefits, perceives barriers, cues to action, general health motivation and self-efficacy. Participants are asked to rate all items on a 5-point Likert scale, ranging from strongly disagree (score=1) to strongly agree (score=5). A minimum score of 23 and a maximum score of 115 can be achieved. A higher score reflects a higher motivation to change their lifestyle and health behaviour for dementia risk reduction. The Dutch version of the MCLHB-DRR scale, consisting of 23 items, has shown to be valid in the Dutch general population aged between 30 and 80 years old [58].

For peer review only

**4)** Percentage of participants that indicated in the questionnaire that they have followed up the tailor-made online lifestyle advice ("On what risk factors did you receive lifestyle advice?" and "Did you follow up the tailor-made lifestyle advice since the last questionnaire (with regard to [risk factor])"? , but also the percentage of participants that indicated that they have followed up the advice to consult their General Practitioner ("Did you have contact with your general practitioner after receiving feedback on the risk and protective factors?").

**Statistical analyses**

First, descriptive characteristics will be explored. The difference in uptake between the two recruitment strategies will be examined using multilevel logistic regression analyses in order to correct for clustering at memory clinic level. We will calculate the percentage with the corresponding 95% confidence interval (CI) and use an alpha of 0.05 to test statistical significance.
The effectiveness of the online tailor-made lifestyle program for dementia risk reduction will be determined by, firstly comparing the change in LIBRA score, the individual risk factors and the MCLHB-DRR score between the active and passive recruitment strategy, and secondly comparing participants of the Demin study (active and passive recruitment strategy) to a control group consisting of Lifelines participants (large population-based cohort study (n> 167.000)) (www.lifelines.nl)[59] in outcome. Lifelines participants (age 40 – 60 years) with a parent with dementia will be matched (using propensity scores) on non-modifiable risk factors (age, gender and education) for dementia to participants of the Demin. Subsequently, multilevel analyses will be performed to examine the change in the LIBRA score and the individual health behaviours over time. In addition, possible confounding and interaction effects will be identified and corrected for in the analysis. We will calculate relative risks (RR) with 95% confidence intervals (CI) and use an alpha of 0.05 to test significance.

**Adverse events**

The risk classification of this intervention is considered negligible, since only information and health advice is provided. Serious adverse events as a result of the intervention are not expected, thus no data safety and monitoring board is installed. Potential participants are informed about possible adverse



For peer review only - http://bmjopen.bmj.com/site/about/guidelines.xhtml

409 events. For example dementia risk assessment may help raising the awareness of their susceptibility in order to motivate health behaviour change [28], however risk assessment could also have an unfavourable effect. Participants may become anxious about developing dementia and could experience more stress if they receive their health profile. Therefore, participants are clearly informed that the presence or absence of risk and protective factors is not a reassurance that they will develop dementia later in life. Furthermore, participants are informed that there is the possibility that unexpected medical findings can be found. In this case, participants receive a separate message in their personal inbox with the recommendation to contact their general practitioner to verify the results (hypertension, high cholesterol, renal dysfunction, diabetes) and discuss whether treatment is needed. Participants may consider online risk assessment as a privacy risk. In this study, all personal information is kept separately from the research data, and participants use a SMS-tan code to login in their personal account.

**Patient and Public Involvement**

Descendants of people with dementia were involved in the development of the Demin website. We assessed the knowledge, beliefs and attitudes towards dementia and dementia risk reduction among descendants of people with dementia (focus group discussions). The results of the focus group discussions were used to develop the Demin website in order to improve the participant recruitment and encourage health behaviour change among participants.

**Ethics and dissemination**

This study is approved by the Dutch ministry of Health, Welfare and Sport according to the Dutch Population Screening Act. Research which is considered to be Population Screening on the ground of the Population Screening Act, for which ministerial approval is required, does not have to be assessed on the basis of the Medical Research Involving Human Subjects Act [60]. Population screening is defined as ‘medical research in persons carried out on an entire population or a category thereof aimed at the detection of certain types of disease or certain risk indicators for the benefit of the participating subjects’[61]. This project focuses on the attenuations of risk factors for dementia. Since these risk



For peer review only - http://bmjopen.bmj.com/site/about/guidelines.xhtml

factors are merely lifestyle factors, a positive impact beyond dementia may be expected. Due to a healthy lifestyle more healthy life years are added to people's lives, which may ultimately increase the risk on dementia as age is an important risk factor for dementia. This research is conducted in accordance to the international ethical guidelines [62].

All participants give informed consent to participate in this study, by signing an electronic informed consort form using SMS-tan (see **Supplementary file 3**). Authorship will be allocated using the guidelines for authorship defined by the International Committees of Medical Journal Editors (ICMJE) [63]. The results of the trial will be submitted to an international peer-reviewed journal and presented at national and international conferences.

**Acknowledgements** The authors would like to thank the Board of Directors and the collaborators of the participating memory clinics for the local approval and collaboration to conduct this multicentre study: Albert Schweitzer hospital (Dordrecht), Gelre hospital (Apeldoorn), University Medical Centre Groningen (Groningen), Medical Centre Leeuwarden (Leeuwarden), Nij Smellinghe (Drachten), Isala Zwolle (Zwolle), Martini hospital (Groningen), Haga hospital (Den Haag), Scheper hospital (Emmen), Refaja hospital (Stadskanaal), St. Jans Gasthuis (Weert), University Medical Centre Utrecht (Utrecht), Reinier de Graaf Gasthuis (Delft), Maxima Medical Centre (Eindhoven), Radboud University Medical Centre (Nijmegen), TweeSteden hospital (Tilburg), Erasmus Medical Centre (Rotterdam), Ommelanden Hospital Groningen (Scheemda), Rijnstate hospital (Arnhem and Zevenaar).

**Collaborators** The members of the Demin consortium are: Elske Marije Abma (MD), Ameen Abu-Hanna (PhD), Erik Buskens (MD, PhD), Jürgen Claassen (MD, PhD), Peter Paul De Deyn (MD, PhD), Theo Feitsma (MD), Liesbeth Hempenius (MD, PhD), Jan Hoogmoed (MD), Ad Kamper (MD, PhD), Dineke Koek (MD, PhD), Jolijn Kragt (MD, PhD), Joep Lagro (MD, PhD), Els Lambooij (MD), Marc Langedijk (MD), Els Maeckelberghe (PhD), Francesco Mattace Raso (MD, PhD), Marieke Meinardi (MD, PhD), Richard Oude Voshaar (MD, PhD), Fransje Reesink (MD, PhD), Sophia de Rooij (MD,



For peer review only - http://bmjopen.bmj.com/site/about/guidelines.xhtml

PhD), Antoinette Scheepmaker (MD), Nynke Smidt (PhD), Petra Spies (MD), Diana Taekema (MD, PhD), Jos Verkuyl (MD), Ralf Vingerhoets (MD), Joyce Vrijsen (MSc), Andrea de Winter (PhD).

**Author contributions** JV contributed to the study concept and design, drafting of the manuscript and critical revision of the manuscript. NS and SR conceived the idea, were responsible for data acquisition, contributed to the study concept and design, and the critical revision of the manuscript. AAH, EM, PD, AW, FR, ROV, and EB contributed to the study concept and design, and the critical revision of the manuscript. All authors read and approved the final manuscript.

**Funding** This study was supported by grants from the Netherlands Organisation for Health Research and Development (ZonMw), subprogram prevention program (project number: 531002008).

**Competing interests** None declared

**Data availability** The data collected during this study will be available from the corresponding author upon reasonable request. This study aims to include data from 378 middle-aged participants recruited through participating memory clinics in the Netherlands. The final dataset will include data on physical examination, laboratory data from fasting blood samples and self-reported data including demographic characteristics, health and health behaviour. We will make the data and associated documentation available to users conditional on a data-sharing agreement that provides for: (1) a commitment to using the data only for research purposes, (2) a commitment to securing the data using appropriate computer technology, and (3) a commitment to destroying or returning the data after analyses are completed.

**Patient consent** Obligatory

**Ethics approval** This study is approved by the Dutch ministry of Health, Welfare and Sport according to the population screening act. In addition, all participating memory clinics approved the study.

**Supplementary files**

Supplementary file 1 Measures Dementia Risk Assessment

Supplementary file 2 Overview of measurements at baseline and follow up

Supplementary file 3 Consent form model

For peer review only

492 **REFERENCES**

493 1 World Health Organization. Dementia: a public health priority. *World Heal Organ* 2012;:112.

494 2 Prince M, Wimo A, Guerchet M, *et al.* World Alzheimer Report 2015: The Global Impact of
495 Dementia - An analysis of prevalence, incidence, cost and trends. *Alzheimer's Dis Int* 2015;:84.
496 doi:10.1111/j.0963-7214.2004.00293.x

497 3 Wimo A, Guerchet M, Ali G-C, *et al.* The worldwide costs of dementia 2015 and comparisons with
498 2010. *Alzheimer's Dement* 2017;**13**:1–7. doi:10.1016/j.jalz.2016.07.150

499 4 Norton S, Matthews FE, Barnes DE, *et al.* Potential for primary prevention of Alzheimer's disease:
500 an analysis of population-based data. *Lancet Neurol* 2014;**13**:788–94. doi:10.1016/S1474-
501 4422(14)70136-X

502 5 Sindi S, Mangialasche F, Kivipelto M. Advances in the prevention of Alzheimer's Disease.
503 *F1000Prime Rep* 2015;**7**:50. doi:10.12703/P7-50

504 6 Livingston G, Sommerlad A, Orgeta V, *et al.* The Lancet Commissions Dementia prevention,
505 intervention, and care. Published Online First: 2017. doi:10.1016/S0140-6736(17)31363-6

506 7 Fratiglioni L, Launer LJ, Andersen K, *et al.* Incidence of dementia and major subtypes in Europe:
507 A collaborative study of population-based cohorts. *Neurology* 2000;**54**:1–10.

508 8 Rizzi L, Rosset I, Roriz-Cruz M. Global Epidemiology of Dementia: Alzheimer's and Vascular
509 Types. *Biomed Res Int* 2014;**2014**:1–8. doi:10.1155/2014/908915

510 9 Scarabino D, Gambina G, Broggio E, *et al.* Influence of family history of dementia in the
511 development and progression of late-onset Alzheimer's disease. *Am J Med Genet Part B*
512 *Neuropsychiatr Genet* 2016;**171**:250–6. doi:10.1002/ajmg.b.32399

513 10 van Exel E, Eikelenboom P, Comijs H, *et al.* Vascular factors and markers of inflammation in
514 offspring with a parental history of late-onset Alzheimer disease. *Arch Gen Psychiatry*
515 2009;**66**:1263–70. doi:10.1001/archgenpsychiatry.2009.146

516 11 Muñoz M, Pong-Wong R, Canela-Xandri O, *et al.* Evaluating the contribution of genetics and
517 familial shared environment to common disease using the UK Biobank. *Nat Genet*
518 2016;**48**:980–3. doi:10.1038/ng.3618

519 12 Borenstein AR, Copenhaver CI, Mortimer JA. Early-Life Risk Factors for Alzheimer Disease.



For peer review only - http://bmjopen.bmj.com/site/about/guidelines.xhtml

*Alzheimer Dis Assoc Disord* 2006;**20**:63–72. doi:10.1097/01.wad.0000201854.62116.d7

13 Andersson L, Stanich J. Life events and their impact on health attitudes and health behavior. *Arch Gerontol Geriatr* 1996;**23**:163–77. doi:10.1016/0167-4943(96)00716-9

14 Wolters FJ, van der Lee SJ, Koudstaal PJ, *et al.* Parental family history of dementia in relation to subclinical brain disease and dementia risk. *Neurology* 2017;**88**:1642–9. doi:10.1212/WNL.0000000000003871

15 Deckers K, van Boxtel MPJ, Schiepers OJG, *et al.* Target risk factors for dementia prevention: a systematic review and Delphi consensus study on the evidence from observational studies. *Int J Geriatr Psychiatry* 2015;**30**:234–46. doi:10.1002/gps.4245

16 de Bruijn RF, Bos MJ, Portegies ML, *et al.* The potential for prevention of dementia across two decades: the prospective, population-based Rotterdam Study. *BMC Med* 2015;**13**:132. doi:10.1186/s12916-015-0377-5

17 Ngandu T, Lehtisalo J, Solomon A, *et al.* A 2 year multidomain intervention of diet, exercise, cognitive training, and vascular risk monitoring versus control to prevent cognitive decline in at-risk elderly people (FINGER): a randomised controlled trial. *Lancet (London, England)* 2015;**385**:2255–63. doi:10.1016/S0140-6736(15)60461-5

18 van Charante EPM, Richard E, Eurelings LS, *et al.* Effectiveness of a 6-year multidomain vascular care intervention to prevent dementia (preDIVA): a cluster-randomised controlled trial. *Lancet* 2016;**388**:797–805. doi:10.1016/S0140-6736(16)30950-3

19 Vellas B, Carrie I, Gillette-Guyonnet S, *et al.* MAPT Study: A Multidomain Approach for Preventing Alzheimer's Disease: Design and Baseline Data. *J Prev Alzheimer's Dis* 2014;**1**:13–22.

20 Singh-Manoux A, Kivimaki M, Glymour MM, *et al.* Timing of onset of cognitive decline: results from Whitehall II prospective cohort study. *BMJ* 2012;**344**:d7622. doi:10.1136/BMJ.D7622

21 Sperling RA, Aisen PS, Beckett LA, *et al.* Toward defining the preclinical stages of Alzheimer's disease: recommendations from the National Institute on Aging-Alzheimer's Association workgroups on diagnostic guidelines for Alzheimer's disease. *Alzheimers Dement* 2011;**7**:280–92. doi:10.1016/j.jalz.2011.03.003



For peer review only - http://bmjopen.bmj.com/site/about/guidelines.xhtml

22 Rajan KB, Wilson RS, Weuve J, *et al.* Cognitive impairment 18 years before clinical diagnosis of Alzheimer disease dementia. *Neurology* 2015;**85**:898–904. doi:10.1212/WNL.0000000000001774

23 Webb TL, Joseph J, Yardley L, *et al.* Using the internet to promote health behavior change: a systematic review and meta-analysis of the impact of theoretical basis, use of behavior change techniques, and mode of delivery on efficacy. *J Med Internet Res* 2010;**12**:e4. doi:10.2196/jmir.1376

24 van Stralen MM, de Vries H, Mudde AN, *et al.* The long-term efficacy of two computer-tailored physical activity interventions for older adults: main effects and mediators. *Health Psychol* 2011;**30**:442–52. doi:10.1037/a0023579

25 Schulz DN, Kremers SPJ, Vandelanotte C, *et al.* Effects of a web-based tailored multiple-lifestyle intervention for adults: a two-year randomized controlled trial comparing sequential and simultaneous delivery modes. *J Med Internet Res* 2014;**16**:e26. doi:10.2196/jmir.3094

26 Jahangiry L, Montazeri A, Najafi M, *et al.* An interactive web-based intervention on nutritional status, physical activity and health-related quality of life in patient with metabolic syndrome: a randomized-controlled trial (The Red Ruby Study). *Nutr Diabetes* 2017;**7**:e240. doi:10.1038/nutd.2016.35

27 Murray E. Web-based interventions for behavior change and self-management: potential, pitfalls, and progress. *Med 20* 2012;**1**:e3. doi:10.2196/med20.1741

28 Janz NK, Becker MH. The Health Belief Model: A Decade Later. *Heal Educ Behav* 1984;**11**:1–47. doi:10.1177/109019818401100101

29 Prochaska JO, Velicer WF. The transtheoretical model of health behavior change. *Am J Health Promot* 1997;**12**:38–48.http://www.ncbi.nlm.nih.gov/pubmed/10170434 (accessed 24 Feb 2017).

30 Hagger MS, Chatzisarantis NLD. Integrating the theory of planned behaviour and self-determination theory in health behaviour: A meta-analysis. *Br J Health Psychol* 2009;**14**:275–302. doi:10.1348/135910708X373959

31 Bandura A. Social Cognitive Theory: An Agentic Perspective. *Annu Rev Psychol* 2001;**52**:1–26.

For peer review only



For peer review only - http://bmjopen.bmj.com/site/about/guidelines.xhtml

doi:10.1146/annurev.psych.52.1.1

32 de Vries H. An Integrated Approach for Understanding Health Behavior; The I-Change Model as an Example. *Psychol Behav Sci Int J* 2017;**2**. doi:10.19080/pbsij.2017.02.555585

33 Webb TL, Joseph J, Yardley L, *et al.* Using the internet to promote health behavior change: a systematic review and meta-analysis of the impact of theoretical basis, use of behavior change techniques, and mode of delivery on efficacy. *J Med Internet Res* 2010;**12**:e4. doi:10.2196/jmir.1376

34 Koopmans B, Nielen MM, Schellevis FG, *et al.* Non-participation in population-based disease prevention programs in general practice. *BMC Public Health* 2012;**12**:856. doi:10.1186/1471-2458-12-856

35 Yancey AK, Ortega AN, Kumanyika SK. EFFECTIVE RECRUITMENT AND RETENTION OF MINORITY RESEARCH PARTICIPANTS. *Annu Rev Public Health* 2006;**27**:1–28. doi:10.1146/annurev.publhealth.27.021405.102113

36 Bracken K, Askie L, Keech AC, *et al.* Recruitment strategies in randomised controlled trials of men aged 50 years and older: a systematic review. *BMJ Open* 2019;**9**. doi:10.1136/BMJOPEN-2018-025580

37 Heger I, Deckers K, van Boxtel M, *et al.* Dementia awareness and risk perception in middle-aged and older individuals: baseline results of the MijnBreincoach survey on the association between lifestyle and brain health. *BMC Public Health* 2019;**19**:678. doi:10.1186/s12889-019-7010-z

38 Rosenberg A, Coley N, Soulier A, *et al.* Experiences of dementia and attitude towards prevention: a qualitative study among older adults participating in a prevention trial. *BMC Geriatr* 2020;**20**:99. doi:10.1186/s12877-020-1493-4

39 Centraal Bureau voor de Statistiek. Percentage hoger opgeleiden van 15 laar en ouder - Bevolkingskern (2011). 2011.http://www.cbsinuwbuurt.nl/#bevolkingskern2011_percentage_inwoners_hogere_opleiding (accessed 18 Feb 2019).

40 DONNER A, BIRKETT N, BUCK C. RANDOMIZATION BY CLUSTER. *Am J Epidemiol* 1981;**114**:906–14. doi:10.1093/oxfordjournals.aje.a113261

For peer review only



For peer review only - http://bmjopen.bmj.com/site/about/guidelines.xhtml

41 Kul S, Vanhaecht K, Panella M. Intraclass correlation coefficients for cluster randomized trials in care pathways and usual care: hospital treatment for heart failure. *BMC Health Serv Res* 2014;**14**:84. doi:10.1186/1472-6963-14-84

42 Rutterford C, Copas A, Eldridge S. Methods for sample size determination in cluster randomized trials. *Int J Epidemiol* 2015;**44**:1051–67. doi:10.1093/ije/dyv113

43 Meppelink CS, Weert JC van, Haven CJ, *et al.* The Effectiveness of Health Animations in Audiences With Different Health Literacy Levels: An Experimental Study. *J Med Internet Res* 2015;**17**. doi:10.2196/jmir.3979

44 Mayer R. Multimedia Learning. In: Ross BH, ed. *The psychology of learning and motivation*. San Diego: : Academic Press 2002. 85–139.

45 Paivio A. *Mental representations : a dual coding approach*. Oxford University Press 1986.

46 Jones CR, Taylor K, Poston L, *et al.* Validation of the Welch Allyn 'Vital Signs' oscillometric blood pressure monitor. *J Hum Hypertens* 2001;**15**:191–5. doi:10.1038/sj.jhh.1001141

47 Schiepers OJG, Köhler S, Deckers K, *et al.* Lifestyle for Brain Health (LIBRA): a new model for dementia prevention. *Int J Geriatr Psychiatry* Published Online First: February 2017. doi:10.1002/gps.4700

48 Deckers K, van Boxtel MPJ, Schiepers OJG, *et al.* Target risk factors for dementia prevention: a systematic review and Delphi consensus study on the evidence from observational studies. *Int J Geriatr Psychiatry* 2015;**30**:234–46. doi:10.1002/gps.4245

49 Vos SJB, van Boxtel MPJ, Schiepers OJG, *et al.* Modifiable Risk Factors for Prevention of?Dementia in Midlife, Late Life and the Oldest-Old: Validation of the LIBRA Index. *J Alzheimer's Dis* 2017;**58**:537–47. doi:10.3233/JAD-161208

50 De Grauw W, De Leest K, Schenk P, *et al.* NHG-Standaard Chronische Nierschade. *TPO - Prakt* 2018;**13**:26–9. doi:10.1007/s12503-018-0065-y

51 Gezondheidsraad. Richtlijnen goede voeding 2015 Gezondheidsraad. https://www.gezondheidsraad.nl/sites/default/files/201524_richtlijnen_goede_voeding_2015.pdf (accessed 20 Mar 2017).

52 Gezondheidsraad. Beweegrichtlijnen 2017. Den Haag: 2017.

For peer review only

53 Rutten G, De Grauw W, Nijpels G, *et al.* NHG-Standaard Diabetes mellitus type 2. 2013.

54 NHG-Standaard Cardiovasculair risicomanagement(Tweede herziening). *Huisarts Wet* 2012;**1**:14–28.https://www.nhg.org/standaarden/volledig/cardiovasculair-risicomanagement (accessed 20 Jun 2017).

55 Deckers K, Kohler S, van Boxtel M, *et al.* Lack of associations between modifiable risk factors and dementia in the very old: findings from the Cambridge City over-75s cohort study. *Aging Ment Health* 2017;:1–7. doi:10.1080/13607863.2017.1280767

56 Kim S, Sargent-Cox K, Cherbuin N, *et al.* Development of the motivation to change lifestyle and health behaviours for dementia risk reduction scale. *Dement Geriatr Cogn Dis Extra* 2014;**4**:172–83. doi:10.1159/000362228

57 Beaton DE, Bombardier C, Guillemin F, *et al.* Guidelines for the Process of Cross-Cultural Adaptation of Self-Report Measures. *Spine (Phila Pa 1976)* 2000;**25**:3186–91. doi:10.1097/00007632-200012150-00014

58 Joxhorst T, Vrijsen J, Niebuur J, *et al.* Cross-cultural validation of the motivation to change lifestyle and health behaviours for dementia risk reduction scale in the Dutch general population. *BMC Public Health* 2020;**20**:788. doi:10.1186/s12889-020-08737-y

59 Scholtens S, Smidt N, Swertz MA, *et al.* Cohort Profile: LifeLines, a three-generation cohort study and biobank. *Int J Epidemiol* 2015;**44**:1172–80. doi:10.1093/ije/dyu229

60 Central Committee on Research Involving Human Subjects. Population Screening Act. 2020.https://english.ccmo.nl/investigators/legal-framework-for-medical-scientific-research/laws/population-screening-act (accessed 18 Jun 2020).

61 Central Committee on Research Involving Human Subjects. The definition of population screening. 2020.https://english.ccmo.nl/investigators/types-of-research/other-types-of-research/population-research/definition (accessed 24 Jun 2020).

62 Council for International Organizations of Medical Sciences. International Ethical Guidelines for Biomedical Research Involving Human Subjects. *Bull Med Ethics* 2002;**182**:17–23.

63 Editorial A. Recommendations for the Conduct, Reporting, Editing and Publication of Scholarly Work in Medical Journals. *Curr Pediatr* 2017;**16**:90–106. doi:10.15690/vsp.v16i2.1710



For peer review only

For peer review only - http://bmjopen.bmj.com/site/about/guidelines.xhtml

**License statement** I, the Submitting Author has the right to grant and does grant on behalf of all authors of the Work (as defined in the below author licence), an exclusive licence and/or a non-exclusive licence for contributions from authors who are: i) UK Crown employees; ii) where BMJ has agreed a CC-BY licence shall apply, and/or iii) in accordance with the terms applicable for US Federal Government officers or employees acting as part of their official duties; on a worldwide, perpetual, irrevocable, royalty-free basis to BMJ Publishing Group Ltd ("BMJ") its licensees and where the relevant Journal is co-owned by BMJ to the co-owners of the Journal, to publish the Work in BMJ Open and any other BMJ products and to exploit all rights, as set out in our licence.

The Submitting Author accepts and understands that any supply made under these terms is made by BMJ to the Submitting Author unless you are acting as an employee on behalf of your employer or a postgraduate student of an affiliated institution which is paying any applicable article publishing charge ("APC") for Open Access articles. Where the Submitting Author wishes to make the Work available on an Open Access basis (and intends to pay the relevant APC), the terms of reuse of such Open Access shall be governed by a Creative Commons licence – details of these licences and which Creative Commons licence will apply to this Work are set out in our licence referred to above.

For peer review only

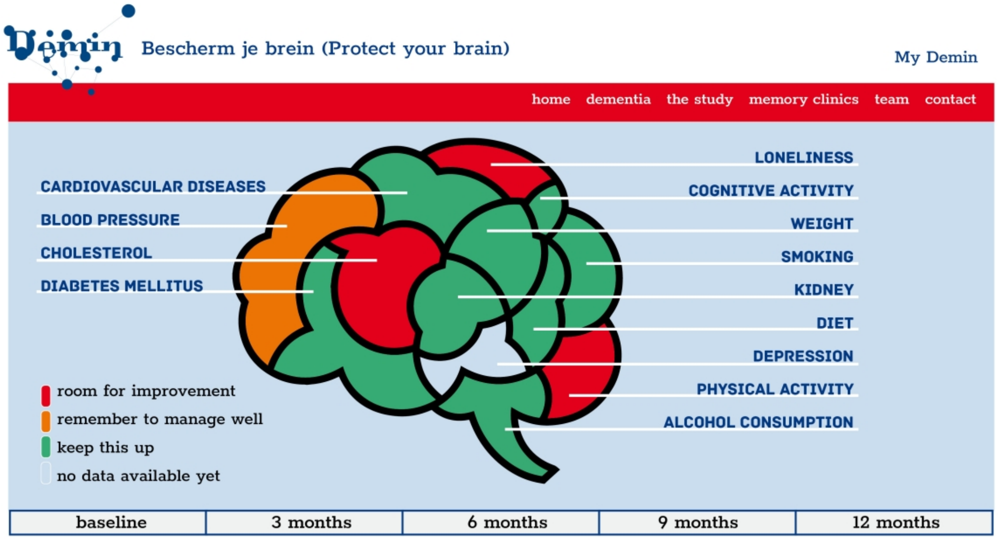


Figure 1. Example of a personal health profile

291x158mm (300 x 300 DPI)

**Supplementary file 1: Measures Dementia Risk Assessment**

Through the online questionnaire and physical examination, data on thirteen currently known protective (i.e. Mediterranean diet, low/moderate alcohol consumption, cognitive activity) and risk factors (i.e. physical inactivity, smoking, loneliness, cardiovascular diseases, hypertension, high cholesterol, diabetes mellitus, obesity, renal dysfunction, depression) for dementia are collected [1–3]. The measurements of these risk and protective factors are described below.

**Protective factors**

**Mediterranean diet**

The Mediterranean-DASH diet intervention for neurodegenerative delay (MIND) has shown to slow down cognitive decline [4] and to decrease the risk of developing AD [5]. Therefore, adherence to the MIND-diet is determined with a number of items of the Food Frequency Questionnaire (FFQ), which is a reliable and valid instrument to measure intake of a specified list of food items in the general populations [6,7]. The following healthy food groups of the MIND-diet were included in the questionnaire, such as vegetables (especially green leafy vegetables), nuts, berries, beans, whole grains, seafood, poultry, olive oil [4,5]. Also five unhealthy food groups of the MIND-diet including red meat, butter, cheese, sweets and fried/fast food were asked [4,5]. Based on the intake of the food groups, adherence to the MIND-diet is determined (0-14). A score of 14 represent good adherence to the MIND-diet *(See **Table 1** for the MIND-diet scoring table).*

**Table 1.** MIND-diet scoring table [5]

| MIND components | Recommended quantity | Max score |
|---|---|---|
| Whole grains | ≥ 3 serving spoons / day | 1 |
| Green leafy | ≥ 6 serving spoons / week | 1 |
| Other vegetables | ≥ 1 serving spoon / day | 1 |
| Berries (including other fruits) | ≥ 2 portions / week * | 1 |
| Red Meats and products | < 4 portions / week | 1 |

For peer review only

| | | |
|---|---|---|
| Fish | ≥ 1 portion / week | 1 |
| Poultry | ≥ 2 portions / week | 1 |
| Beans | > 3 serving spoons /week | 1 |
| Nuts | ≥ 5 portions /week * | 1 |
| Fast/ fried food | < 1time / week | 1 |
| Butter, margarine | < 1 teaspoon/ day | 1 |
| Cheese | < 1 slice / week | 1 |
| Pastries, sweets | < 5 portions / week | 1 |
| Olive Oil (used as primary oil) | yes | 1 |
| **Total score** | | **14** |

** One portion is a handful of the given component*

**Low/moderate alcohol consumption**

Alcohol consumption was measured using the FFQ [6], including questions regarding the frequency of alcohol use (e.g. no consumption last month, 1 day per month, 2-3 days per month, 1 day per week, 2-3 days per week, 4-5 days per week, 6-7 days per week) and the average number of glasses of alcohol per day (range from zero to more than twelve) was asked. Subsequently, the average number of glasses per month was calculated in order to classify participants into: (i) non-alcohol consumers, (ii) low/moderate alcohol consumers or (iii) excessive alcohol consumers [8]. Participants adhere to the national recommendations for no to low/moderate alcohol consumption, if participants drink one glass or less alcohol per day on average, without binge drinking (more than three glasses alcohol on one day for females and more than four glasses alcohol on one day for males)) [9].

**High cognitive activity**

Cognitive activity is assessed with the leisure time section of the Cognitive Reserve Index questionnaire (CRIq) (22). CRIq aims to measure cognitive reserve (CR), which is based on education, working activity and leisure time activity. For this study we are interested in the current cognitive

For peer review only

activities of the participants. Therefore, cognitive activity is determined by measuring working activity and leisure time activity. The frequency of eighteen leisure time activity is asked (e.g. (i) never, (ii) less than once a month, (iii) once a month, (iv) once every 2 weeks, (v) several times a week). Subsequently, a leisure time cognitive activity score is calculated, ranging from to 18 to 108, where a score of 50 or higher represent high cognitive activity (based on results of a survey on the knowledge, beliefs and attitudes towards dementia risk reduction among the general population of Groningen, see Table 2 and 3).

Additionally, participants are asked if they have a paid job and if so how many hours they spend on their job per week. High cognitive activity is defined as (i) working at least 24 hours per week or (ii) a leisure time cognitive activity score of at least 50.

For peer review only

**Table 2.** Cognitive activity (leisure time) scores stratified for education level and having a paid job in a survey conducted among the general population in Groningen

| **Education level** | **Work** | **Leisure time score** | |
|---|---|---|---|
| | | mean(SD) | (min-max) |
| Low (n=105) | no work (n=75) | 39.57 (11.16) | 13 – 63 |
| | work (n=30) | 41.73 (11.12) | 25 – 68 |
| Middle (n=154) | no work (n=72) | 47.03 (9.95) | 26 – 76 |
| | work (n=82) | 45.20 (9.49) | 25 – 64 |
| High (n=390) | no work (n= 135) | 51.93 (10.19) | 18 – 75 |
| | work (n=255) | 48.32 (8.97) | 23 – 74 |

**Table 3.** Cognitive activity (leisure time) scores stratified for education level and having a paid job in a survey conducted among the general population in Groningen (subgroup: 40 – 60 year old)

| **Education level** | **Work** | **Leisure time score** | |
|---|---|---|---|
| | | mean(SD) | (min-max) |

| | | | |
|---|---|---|---|
| Low (n=29) | no work (n=7) | 39.71 (9.67) | 21 – 49 |
| | work (n=22) | 40.50 (10.52) | 25 – 58 |
| Middle (n=68) | no work (n=9) | 43. 89 (13.15) | 26 – 66 |
| | work (n=59) | 46.34 (8.87) | 25 – 64 |
| High (n=140) | no work (n= 16) | 50.56 (10.59) | 37 – 69 |
| | work (n=124) | 49.91 (9.24) | 23 – 74 |

**Risk factors**

**Physical inactivity**

Physical activity levels are determined using the Short Questionnaire to Asses Health enhancing physical activity (SQUASH), a self-reported questionnaire and commonly used instrument in the Netherlands to assess physical activity [10]. The SQUASH questionnaire has shown to be valid and reliable in measuring physical activity among the Dutch population [11–14]. The SQUASH questionnaire includes questions on multiple activities referring to an average week in the last month, including actively commuting (walking, cycling) to (voluntary) work or school, physical activity at (voluntary) work or school, household activities and leisure time activities, including walking, cycling, gardening and sports. Participants were asked to fill in how many days a week they engaged in the activities (frequency), the average time per day spent on each activity (hours and minutes; duration) and the intensity at which they did the activity (low, moderate, high) [10]. A standardized methodology was followed to calculate physical activity levels. Briefly, results from the SQUASH questionnaire are automatically converted to minutes per week spent in light (LPA) and moderate to vigorous (MVPA) intensity activities based on Metabolic Equivalent Tasks (METs) derived from the Ainsworth's compendium of physical activity [15]. Physical activity levels are divided into the following categories: 0 minutes MVPA per week, 0 to 149 minutes MVPA per week, 150 to 299 minutes MVPA per week and 300 minutes MVPA per week and more. Physical inactivity is defined as less than 150 minutes per week MVPA [16].

For peer review only

Additionally, the questionnaire contained information on sitting behaviour, which is divided into sitting during transportation, working hours, watching television or using the computer at home. Participants are asked to fill in the number of hours and minutes on an average day in the past seven days during the week and on an average day during the weekend. This is similar to the sitting measure of the International Physical Activity Questionnaire (IPAQ) which has shown to be valid and reliable [17]. Sitting time was divided into the following 4 categories: (i) less than 4 hours a day, (ii) 4 to 8 hours a day, (iii) 8 to 11 hours a day and (iv) at least 11 hours a day or more [18]. Prolonged sitting time was defined as sitting at least for 8 hours a day or more.

Participants are physically inactive if they (i) are sitting on average more than 8 hours a day, irrespective of the physical activity, or (ii) are sitting on average 4 hours or more a day and are less active than 150 minutes MVPA per week.

**Smoking**

Participants are asked three questions to measure smoking behaviour: (i) whether they have smoked in the past month, and (ii) whether they have smoked in the past, for at least one year [19]. Smoking behaviour is categorized into non-smoker, past smoker and current smoker. Current smokers are defined as people who reported smoking in the past month. Past smokers reported smoking for at least one year, but did not smoke in the past month.

**Loneliness**

Loneliness is measured using De Jong Gierveld Loneliness Scale , which is a reliable and valid instrument to measure emotional, social and overall loneliness [20]. Possible answers on this 6-item scale are: (i) yes!, (ii) yes, (iii) more or less, (iv), no, (v) no!. The overall loneliness score is calculated by counting the neutral and negative ("no!", "no", or "more or less") answers on items 4, 5 and 6 (social loneliness score) and by counting the positive ("more or less"", "yes" or "yes!") answers on items 1,2 and 3 (emotional loneliness score). Subsequently, the overall loneliness score is categorized into: (i) not lonely (0-1), (ii) moderate lonely (2-4), (iii) severe lonely (5-6). Loneliness is defined as an overall loneliness score of 2 or higher [20].

For peer review only

 

**Cardiovascular diseases**

Participants are asked whether they have suffered or still suffer from one of the following cardiovascular diseases: angina pectoris, myocardial infarction, transient ischemic attack (TIA), stroke or peripheral arterial diseases (yes/no). Presence of a cardiovascular disease is defined as having at least one of the above mentioned diseases.

**Hypertension**

Hypertension is determined based on the blood pressure measurement in which the systolic and diastolic blood pressure is measured both three times consecutively. The average of the second and the third measurement is used to determine the presence of hypertension. Hypertension is present: (i) if the systolic blood pressure is higher than 140 mmHg or diastolic blood pressure is higher than 90 mmHg [21], or (ii) if participants indicate that they receive medication (i.e. diuretics, beta blockers, ACE-inhibitors, angiotensin 2 antagonists and calcium antagonists) for their hypertension .

**High cholesterol**

High cholesterol is defined based on direct laboratory measurements using the fasting blood samples and self-reported questionnaires. High cholesterol is present if (i) the Low Density Lipoprotein (LDL) is higher than 2.5 mmol/l or (ii) the ratio of total cholesterol (TC) and High Density Lipoprotein (HDL) is higher than 8 mmol/l [22] or (iii) participants indicate that they receive medication (i.e. simvastinin, atorvastatin, rosuvastatin, pravastatin, ezetimib) to lower their cholesterol levels.

**Diabetes Mellitus**

The presence of diabetes mellitus (or impaired blood glucose levels) is based on direct laboratory measurements using the fasting blood samples and self-reported questionnaires. Diabetes Mellitus is defined as: (i) glucose (fasting capillary blood) of 7.0 mmol/l or higher, or (ii) glucose (fasting capillary blood) lower than 7.0 mmol/l accompanied by HbA1C levels higher than 53 mmol/mol [23].

For peer review only

HbA1C provides additional information on the average blood glucose levels during the previous month, while glucose may differ during the day [23].

**Obesity**

Body weight and body height are measured during physical examination in order to determine their Body Mass Index (BMI=kg/$m^2$)[24]. Obesity is present if BMI is 30 kg/$m^2$ or higher [25].

**Renal dysfunction**

The presence of renal dysfunction is based on direct laboratory measurements (serum creatinine levels) using the fasting blood samples and self-reported questionnaires [26]. Subsequently, the estimated glomerular filtration rate (eGFR) is calculated using the 2009 CKD-EPI creatinine equation [27,28] in order to determine participant's renal function [28]. Renal dysfunction is present if (i) eGFR is lower than 60 ml/min/1.73 $m^2$ [29], or (ii) participants indicate that they receive medical treatment for the established renal dysfunction.

**Depression**

The level of depressive symptoms is measured using the Centre for Epidemiologic Studies Depression scale (CES-D). The CES-D consists of 20 items and is a reliable and valid tool to measure the current level of depressive symptoms in the general population [30]. Answer options for each item are: rarely or none of the time (0), some or a little of the time (1), occasionally or a moderate amount of time (2), and most of all of the time (3). Total score varying from 0 to 60, indicates the level of depressive symptoms, a higher score reflects a higher level of depressive symptoms. Depression is defined as (i) having a score of 16 or higher [31], or (ii) participants indicate that they receive medical treatment for their depressive symptoms.

For peer review only

## REFERENCES


1 Livingston G, Sommerlad A, Orgeta V, *et al.* The Lancet Commissions Dementia prevention, intervention, and care. Published Online First: 2017. doi:10.1016/S0140-6736(17)31363-6

2 Deckers K, van Boxtel MPJ, Schiepers OJG, *et al.* Target risk factors for dementia prevention: a systematic review and Delphi consensus study on the evidence from observational studies. *Int J Geriatr Psychiatry* 2015;**30**:234–46. doi:10.1002/gps.4245

3 Schiepers OJG, Köhler S, Deckers K, *et al.* Lifestyle for Brain Health (LIBRA): a new model for dementia prevention. *Int J Geriatr Psychiatry* Published Online First: 28 February 2017. doi:10.1002/gps.4700

4 Morris MC, Tangney CC, Wang Y, *et al.* MIND diet slows cognitive decline with aging. *Alzheimer's Dement* 2015;**11**:1015–22. doi:10.1016/j.jalz.2015.04.011

5 Morris MC, Tangney CC, Wang Y, *et al.* MIND diet associated with reduced incidence of Alzheimer's disease. *Alzheimer's Dement* 2015;**11**:1007–14. doi:10.1016/j.jalz.2014.11.009

6 Molag ML, de Vries JHM, Duif N, *et al.* Selecting informative food items for compiling food-frequency questionnaires: comparison of procedures. *Br J Nutr* 2010;**104**:446–56. doi:10.1017/S0007114510000401

7 Siebelink E, Geelen A, de Vries JHM. Self-reported energy intake by FFQ compared with actual energy intake to maintain body weight in 516 adults. *Br J Nutr* 2011;**106**:274–81. doi:10.1017/S0007114511000067

8 Anstey KJ, Mack HA, Cherbuin N. Alcohol Consumption as a Risk Factor for Dementia and Cognitive Decline: Meta-Analysis of Prospective Studies. *Am J Geriatr Psychiatry* 2009;**17**:542–55. doi:10.1097/JGP.0b013e3181a2fd07

9 Gezondheidsraad. Richtlijnen goede voeding 2015 Gezondheidsraad. https://www.gezondheidsraad.nl/sites/default/files/201524_richtlijnen_goede_voeding_2015.pdf (accessed 20 Mar 2017).


For peer review only

10 Wendel-Vos G, Schuit A. SQUASH: Short Questionnaire to Assess Health enhancing physical activity. Bilthoven, Netherlands: 2004.

11 Wagenmakers R, van den Akker-Scheek I, Groothoff JW, *et al.* Reliability and validity of the short questionnaire to assess health-enhancing physical activity (SQUASH) in patients after total hip arthroplasty. *BMC Musculoskelet Disord* 2008;**9**:141. doi:10.1186/1471-2474-9-141

12 de Hollander EL, Zwart L, de Vries SI, *et al.* The SQUASH was a more valid tool than the OBiN for categorizing adults according to the Dutch physical activity and the combined guideline. *J Clin Epidemiol* 2012;**65**:73–81. doi:10.1016/j.jclinepi.2011.05.005

13 Wendel-Vos G. Reproducibility and relative validity of the short questionnaire to assess health-enhancing physical activity. *J Clin Epidemiol* 2003;**56**:1163–9. doi:10.1016/S0895-4356(03)00220-8

14 Nicolaou M, Gademan MGJ, Snijder MB, *et al.* Validation of the SQUASH Physical Activity Questionnaire in a Multi-Ethnic Population: The HELIUS Study. *PLoS One* 2016;**11**:e0161066. doi:10.1371/journal.pone.0161066

15 Ainsworth BE, Haskell WL, Herrmann SD, *et al.* 2011 Compendium of Physical Activities: a second update of codes and MET values. *Med Sci Sports Exerc* 2011;**43**:1575–81. doi:10.1249/MSS.0b013e31821ece12

16 Gezondheidsraad. Beweegrichtlijnen 2017. Den Haag: 2017. file://zkh/dfs/Gebruikers22/VrijsenJ/Data/Downloads/Beweegrichtlijnen_2017.pdf (accessed 14 Sep 2017).

17 CRAIG CL, MARSHALL AL, SJÖSTRÖM M, *et al.* International Physical Activity Questionnaire: 12-Country Reliability and Validity. *Med Sci Sport Exerc* 2003;**35**:1381–95. doi:10.1249/01.MSS.0000078924.61453.FB

18 van der Ploeg HP, Chey T, Korda RJ, *et al.* Sitting Time and All-Cause Mortality Risk in 222 497 Australian Adults. *Arch Intern Med* 2012;**172**:494. doi:10.1001/archinternmed.2011.2174

For peer review only

19 World Health Organization. Global Adult Tobacco Survey Collaborative Group. Tobacco Questions for Surveys: A Subset of Key Questions from the Global Adult Tobacco Survey (GATS) 2 nd Edition. 2011;:6–9. doi:10.1007/s00406-006-0651-9

20 Gierveld JDJ, Tilburg T Van. A 6-Item Scale for Overall, Emotional, and Social Loneliness. *Res Aging* 2006;**28**:582–98. doi:10.1177/0164027506289723

21 NHG-Standaard Cardiovasculair risicomanagement(Tweede herziening). *Huisarts Wet* 2012;**1**:14–28.https://www.nhg.org/standaarden/volledig/cardiovasculair-risicomanagement (accessed 20 Jun 2017).

22 NHG-Standaard Cardiovasculair risicomanagement(Tweede herziening). *Huisarts Wet* 2012;**1**:14–28.

23 Rutten G, De Grauw W, Nijpels G, *et al.* NHG-Standaard Diabetes mellitus type 2. 2013.

24 World Health Organization (WHO). Body mass index - BMI. http://www.euro.who.int/en/health-topics/disease-prevention/nutrition/a-healthy-lifestyle/body-mass-index-bmi (accessed 6 May 2019).

25 Van Binsbergen J, Langens F, Dapper A, *et al.* NHG-Standaard Obesitas | NHG. 2010. https://www.nhg.org/standaarden/volledig/nhg-standaard-obesitas (accessed 4 Dec 2019).

26 Perrone RD, Madias NE, Levey AS. Serum creatinine as an index of renal function: new insights into old concepts. *Clin Chem* 1992;**38**.http://clinchem.aaccjnls.org/content/38/10/1933.short (accessed 4 Apr 2017).

27 Levey AS, Stevens LA, Schmid CH, *et al.* A new equation to estimate glomerular filtration rate. *Ann Intern Med* 2009;**150**:604–12. doi:10.7326/0003-4819-150-9-200905050-00006

28 Levey AS, Inker LA, Coresh J. GFR estimation: From physiology to public health. *Am J Kidney Dis* 2014;**63**:820–34. doi:10.1053/j.ajkd.2013.12.006

29 De Grauw W, De Leest K, Schenk P, *et al.* NHG-Standaard Chronische Nierschade. *TPO - Prakt* 2018;**13**:26–9. doi:10.1007/s12503-018-0065-y


For peer review only

30 Smarr KL, Keefer AL. Measures of depression and depressive symptoms: Beck Depression Inventory-II (BDI-II), Center for Epidemiologic Studies Depression Scale (CES-D), Geriatric Depression Scale (GDS), Hospital Anxiety and Depression Scale (HADS), and Patient Health Questionna. *Arthritis Care Res (Hoboken)* 2011;**63**:S454–66. doi:10.1002/acr.20556

31 Smarr KL, Keefer AL. Measures of depression and depressive symptoms: Beck Depression Inventory-II (BDI-II), Center for Epidemiologic Studies Depression Scale (CES-D), Geriatric Depression Scale (GDS), Hospital Anxiety and Depression Scale (HADS), and Patient Health Questionna. *Arthritis Care Res (Hoboken)* 2011;**63**:S454–66. doi:10.1002/acr.20556

For peer review only

**Supplementary file 2. Overview of assessment measures at baseline and follow up**

**Table 1.** Assessment measures at baseline and follow up

| | Baseline | 3 months | 6 months | 9 months | 12months |
|---|---|---|---|---|---|
| **GENERAL INFORMATION** | | | | | |
| Age, gender, ethnicity, education and postal code | Q | | | | |
| Participation in the Lifelines cohort | Q | | | | |
| Medical family history | Q | | | | |
| Health literacy (S-TOFHLA, 3-items) | Q | | | | |
| **RISK AND PROTECTIVE FACTORS** | | | | | |
| Smoking | Q | Q | Q | Q | Q |
| Physical inactivity (SQUASH, IPAQ sitting measure) | Q | Q | Q | Q | Q |
| Mediterranean diet (FFQ) | Q | Q | Q | Q | Q |
| Alcohol consumption (FFQ) | Q | Q | Q | Q | Q |
| High cognitive activity (CRIq adapted) | Q | Q | Q | Q | Q |
| Loneliness (de Jong Gierveld, 6-items) | Q | Q | Q | Q | Q |
| Cardiovascular diseases (CVD) | Q | Q | Q | Q | Q |
| Obesity (body weight, height) | Q+ PE | Q | Q | Q | Q+PE |
| Hypertension (SBD, DBP) | Q+PE | Q | Q | Q | Q+PE |
| High cholesterol (LDL, HDL, TC) | Q+BS | Q | Q | Q | Q+BS |
| Diabetes Mellitus[1] (glucose, | Q+BS | Q | Q | Q | Q+BS |

For peer review only

| | | | | | |
|---|---|---|---|---|---|
| HbA1C) | | | | | |
| Renal dysfunction (eGFR) | Q+BS | Q | Q | Q | Q+BS |
| Depression (CES-D) | Q | Q | Q | Q | Q |
| **OTHER PARAMETERS** | | | | | |
| Medical treatment of disease | Q | Q | Q | Q | Q |
| Motivation to change lifestyle (MCLHB-DRR) | Q | Q | Q | Q | Q |
| Stages of change | Q | Q | Q | Q | Q |
| Hearing problems | Q | Q | Q | Q | Q |
| Subjective stress (LDI) | Q | | | | Q |
| Memory complaints | Q | | | | |
| Quality of life (2 items of SF36, VAS-score) | Q | | | | Q |
| Perceived living environment | Q | | | | Q |
| Compliance lifestyle advice per individual health behaviour | | Q | Q | Q | Q |
| Compliance advice contact with GP | | Q | Q | Q | Q |

*SQUASH* Short Questionnaire to Assess Health-enhancing physical activity, *IPAQ* International Physical Activity Questionnaire, *FFQ* Food Frequency Questionnaire, *CRIq* Cognitive Reserve Index questionnaire, *CVD* Cardiovascular diseases, *SBP* Systolic Blood Pressure, *DBP* Diastolic Blood Pressure, *HDL* high-density lipoproteins, *LDL* low-density lipoproteins, *TC* total cholesterol, *HbA1C* Hemoglobin A1C, *eGFR* estimated Glomerular Filtration Rate, *CES-D* Centre for Epidemiological Studies Depression Scale, *MCLHB-DRR* Motivation to Change Lifestyle and Health Behavior for Dementia Risk Reduction Scale, LDI Long-term Difficulties Inventory, *SF36* Short Form 36 items, *VAS* Visual Analogue Scale

Q: Online questionnaire; PE: Physical examination; BS: Blood sample

For peer review only

Voornaam Achternaam
Straatnaam 12
1234AB Plaatsnaam
0612345678
demin@umcg.nl
ABCDE
Aangemeld op: 01-02-2020 10:01:16

01-02-2020 10:06:24

Beste Voornaam Achternaam,

Indien u wilt deelnemen aan dit onderzoek, vragen wij u dit toestemmingformulier door middel van een elektronische handtekening te ondertekenen. Hieronder vindt u de voorwaarde voor deelname aan dit onderzoek.

**Wanneer u het toestemmingsformulier ondertekent verklaart u dat:**
- u de informatiefolder met bijlagen en bovenstaande informatie heeft gelezen en hiermee voldoende bent geïnformeerd over het doel en de uitvoering van het onderzoek.
- U de mogelijkheid heeft gehad om aanvullende vragen te stellen (telefonisch of per mail), welke naar tevredenheid zijn beantwoord.
- u genoeg tijd had om te beslissen of u wilt deelnemen.
- u weet dat deelname vrijwillig is en dat u op ieder moment kan beslissen om toch niet mee te doen of te stoppen met het onderzoek.
- u weet dat u op de hoogte gesteld kan worden van medische relevante bevindingen.

**U geeft toestemming:**
– voor deelname aan het landelijk proef-bevolkingsonderzoek naar de beschermende en risicofactoren voor dementie (Demin studie).
– dat u in de toekomst opnieuw benaderd kan worden voor deelname aan aanvullend onderzoek.
– om uw onderzoeksgegevens te koppelen aan gegevens van het Centraal Bureau voor Statistiek (CBS), zoals uw gegevens over woonomgeving (bijvoorbeeld sportfaciliteiten).

Met vriendelijke groet,

Het Demin team
**www.demin.nl**

---------------------------------------------------------------------------------------------------------------
Ik verklaar dat ik deze proefpersoon volledig heb geïnformeerd over het genoemde onderzoek.

Als er tijdens het onderzoek informatie bekend wordt die de toestemming van de proefpersoon zou kunnen beïnvloeden, dan breng ik hem/haar daarvan tijdig op de hoogte.

Naam hoofdonderzoeker:
Handtekening: Datum:
Tijdstip:

*De deelnemer krijgt een volledige informatiebrief, samen met een kopie van het getekende toestemmingsformulier.*

For peer review only

# Reporting checklist for protocol of a clinical trial.

Based on the SPIRIT guidelines.

## Instructions to authors

Complete this checklist by entering the page numbers from your manuscript where readers will find each of the items listed below.

Your article may not currently address all the items on the checklist. Please modify your text to include the missing information. If you are certain that an item does not apply, please write "n/a" and provide a short explanation.

Upload your completed checklist as an extra file when you submit to a journal.

In your methods section, say that you used the SPIRITreporting guidelines, and cite them as:

Chan A-W, Tetzlaff JM, Altman DG, Laupacis A, Gøtzsche PC, Krleža-JeriĆ K, Hróbjartsson A, Mann H, Dickersin K, Berlin J, Doré C, Parulekar W, Summerskill W, Groves T, Schulz K, Sox H, Rockhold FW, Rennie D, Moher D. SPIRIT 2013 Statement: Defining standard protocol items for clinical trials. Ann Intern Med. 2013;158(3):200-207

| | | Reporting Item | Page Number |
|---|---|---|---|
| **Administrative information** | | | |
| Title | #1 | Descriptive title identifying the study design, population, interventions, and, if applicable, trial acronym | 1 |
| Trial registration | #2a | Trial identifier and registry name. If not yet registered, name of intended registry | 3 |
| Trial registration: data set | #2b | All items from the World Health Organization Trial Registration Data Set | All pages, no results |
| Protocol version | #3 | Date and version identifier | 1 |
| Funding | #4 | Sources and types of financial, material, and other support | 21 |
| Roles and responsibilities: contributorship | #5a | Names, affiliations, and roles of protocol contributors | 21 |
| Roles and responsibilities: sponsor contact information | #5b | Name and contact information for the trial sponsor | 1 |

For peer review only

| | | | |
|---|---|---|---|
| Roles and responsibilities: sponsor and funder | #5c | Role of study sponsor and funders, if any, in study design; collection, management, analysis, and interpretation of data; writing of the report; and the decision to submit the report for publication, including whether they will have ultimate authority over any of these activities | 23 |
| Roles and responsibilities: committees | #5d | Composition, roles, and responsibilities of the coordinating centre, steering committee, endpoint adjudication committee, data management team, and other individuals or groups overseeing the trial, if applicable (see Item 21a for data monitoring committee) | 23 |
| **Introduction** | | | |
| Background and rationale | #6a | Description of research question and justification for undertaking the trial, including summary of relevant studies (published and unpublished) examining benefits and harms for each intervention | 5-6 |
| Background and rationale: choice of comparators | #6b | Explanation for choice of comparators | 6 |
| Objectives | #7 | Specific objectives or hypotheses | 7 |
| Trial design | #8 | Description of trial design including type of trial (eg, parallel group, crossover, factorial, single group), allocation ratio, and framework (eg, superiority, equivalence, non-inferiority, exploratory) | 7-8 |
| **Methods: Participants, interventions, and outcomes** | | | |
| Study setting | #9 | Description of study settings (eg, community clinic, academic hospital) and list of countries where data will be collected. Reference to where list of study sites can be obtained | 7 |
| Eligibility criteria | #10 | Inclusion and exclusion criteria for participants. If applicable, eligibility criteria for study centres and individuals who will perform the interventions (eg, surgeons, psychotherapists) | 8-9 |
| Interventions: description | #11a | Interventions for each group with sufficient detail to allow replication, including how and when they will be administered | 9-16 |
| Interventions: modifications | #11b | Criteria for discontinuing or modifying allocated interventions for a given trial participant (eg, drug dose change in response to harms, participant request, or | n/a |

For peer review only

| | | | |
|---|---|---|---|
| | | improving / worsening disease) | |
| Interventions: adherance | #11c | Strategies to improve adherence to intervention protocols, and any procedures for monitoring adherence (eg, drug tablet return; laboratory tests) | 10 |
| Interventions: concomitant care | #11d | Relevant concomitant care and interventions that are permitted or prohibited during the trial | |
| Outcomes | #12 | Primary, secondary, and other outcomes, including the specific measurement variable (eg, systolic blood pressure), analysis metric (eg, change from baseline, final value, time to event), method of aggregation (eg, median, proportion), and time point for each outcome. Explanation of the clinical relevance of chosen efficacy and harm outcomes is strongly recommended | 16-20 |
| Participant timeline | #13 | Time schedule of enrolment, interventions (including any run-ins and washouts), assessments, and visits for participants. A schematic diagram is highly recommended (see Figure) | 11, 16 |
| Sample size | #14 | Estimated number of participants needed to achieve study objectives and how it was determined, including clinical and statistical assumptions supporting any sample size calculations | 9 |
| Recruitment | #15 | Strategies for achieving adequate participant enrolment to reach target sample size | 7 |
| **Methods: Assignment of interventions (for controlled trials)** | | | |
| Allocation: sequence generation | #16a | Method of generating the allocation sequence (eg, computer-generated random numbers), and list of any factors for stratification. To reduce predictability of a random sequence, details of any planned restriction (eg, blocking) should be provided in a separate document that is unavailable to those who enrol participants or assign interventions | 8-9 |
| Allocation concealment mechanism | #16b | Mechanism of implementing the allocation sequence (eg, central telephone; sequentially numbered, opaque, sealed envelopes), describing any steps to conceal the sequence until interventions are assigned | n/a |
| Allocation: implementation | #16c | Who will generate the allocation sequence, who will enrol participants, and who will assign participants to interventions | 8-9 |
| Blinding (masking) | #17a | Who will be blinded after assignment to interventions (eg, trial participants, care providers, outcome assessors, data analysts), and how | n/a, one recruitment strategy per |

For peer review only

| | | | |
|---|---|---|---|
| | | | memory clinic |
| Blinding (masking): emergency unblinding | #17b | If blinded, circumstances under which unblinding is permissible, and procedure for revealing a participant's allocated intervention during the trial | n/a |
| **Methods: Data collection, management, and analysis** | | | |
| Data collection plan | #18a | Plans for assessment and collection of outcome, baseline, and other trial data, including any related processes to promote data quality (eg, duplicate measurements, training of assessors) and a description of study instruments (eg, questionnaires, laboratory tests) along with their reliability and validity, if known. Reference to where data collection forms can be found, if not in the protocol | 11 |
| Data collection plan: retention | #18b | Plans to promote participant retention and complete follow-up, including list of any outcome data to be collected for participants who discontinue or deviate from intervention protocols | 10 |
| Data management | #19 | Plans for data entry, coding, security, and storage, including any related processes to promote data quality (eg, double data entry; range checks for data values). Reference to where details of data management procedures can be found, if not in the protocol | 10-11 |
| Statistics: outcomes | #20a | Statistical methods for analysing primary and secondary outcomes. Reference to where other details of the statistical analysis plan can be found, if not in the protocol | 19 |
| Statistics: additional analyses | #20b | Methods for any additional analyses (eg, subgroup and adjusted analyses) | n/a |
| Statistics: analysis population and missing data | #20c | Definition of analysis population relating to protocol non-adherence (eg, as randomised analysis), and any statistical methods to handle missing data (eg, multiple imputation) | n/a |
| **Methods: Monitoring** | | | |
| Data monitoring: formal committee | #21a | Composition of data monitoring committee (DMC); summary of its role and reporting structure; statement of whether it is independent from the sponsor and competing interests; and reference to where further details about its charter can be found, if not in the protocol. Alternatively, an explanation of why a DMC is not needed | 22 |

For peer review only

| | | | |
|---|---|---|---|
| Data monitoring: interim analysis | #21b | Description of any interim analyses and stopping guidelines, including who will have access to these interim results and make the final decision to terminate the trial | n/a, low risk |
| Harms | #22 | Plans for collecting, assessing, reporting, and managing solicited and spontaneously reported adverse events and other unintended effects of trial interventions or trial conduct | 20-21 |
| Auditing | #23 | Frequency and procedures for auditing trial conduct, if any, and whether the process will be independent from investigators and the sponsor | 16-17 |
| **Ethics and dissemination** | | | |
| Research ethics approval | #24 | Plans for seeking research ethics committee / institutional review board (REC / IRB) approval | 22 |
| Protocol amendments | #25 | Plans for communicating important protocol modifications (eg, changes to eligibility criteria, outcomes, analyses) to relevant parties (eg, investigators, REC / IRBs, trial participants, trial registries, journals, regulators) | n/a |
| Consent or assent | #26a | Who will obtain informed consent or assent from potential trial participants or authorised surrogates, and how (see Item 32) | 22 |
| Consent or assent: ancillary studies | #26b | Additional consent provisions for collection and use of participant data and biological specimens in ancillary studies, if applicable | n/a |
| Confidentiality | #27 | How personal information about potential and enrolled participants will be collected, shared, and maintained in order to protect confidentiality before, during, and after the trial | 21 |
| Declaration of interests | #28 | Financial and other competing interests for principal investigators for the overall trial and each study site | 22 |
| Data access | #29 | Statement of who will have access to the final trial dataset, and disclosure of contractual agreements that limit such access for investigators | 16, 22 |
| Ancillary and post trial care | #30 | Provisions, if any, for ancillary and post-trial care, and for compensation to those who suffer harm from trial participation | n/a, low risk |
| Dissemination policy: trial results | #31a | Plans for investigators and sponsor to communicate trial results to participants, healthcare professionals, the public, and other relevant groups (eg, via publication, reporting in results databases, or other data sharing arrangements), including any publication restrictions | 21 |

For peer review only

| | | | |
|---|---|---|---|
| Dissemination policy: authorship | #31b | Authorship eligibility guidelines and any intended use of professional writers | 21 |
| Dissemination policy: reproducible research | #31c | Plans, if any, for granting public access to the full protocol, participant-level dataset, and statistical code | 21 |
| **Appendices** | | | |
| Informed consent materials | #32 | Model consent form and other related documentation given to participants and authorised surrogates | 21 |
| Biological specimens | #33 | Plans for collection, laboratory evaluation, and storage of biological specimens for genetic or molecular analysis in the current trial and for future use in ancillary studies, if applicable | n/a |

None The SPIRIT checklist is distributed under the terms of the Creative Commons Attribution License CC-BY-ND 3.0. This checklist can be completed online using https://www.goodreports.org/, a tool made by the EQUATOR Network in collaboration with Penelope.ai

For peer review only

BMJ Open

# The uptake and effectiveness of a tailor-made online lifestyle program targeting modifiable risk factors for dementia among middle-aged descendants of people with recently diagnosed dementia: study protocol of a cluster randomised controlled trial (Demin study)

| | |
|---|---|
| Journal: | *BMJ Open* |
| Manuscript ID | bmjopen-2020-039439.R2 |
| Article Type: | Protocol |
| Date Submitted by the Author: | 09-Sep-2020 |
| Complete List of Authors: | Vrijsen, Joyce; University of Groningen, Epidemiology<br>Abu-Hanna, Ameen; University of Amsterdam, Amsterdam UMC, Medical Informatics<br>Maeckelberghe, Els; University of Groningen, University Medical Centre Groningen, Wenckebach Institute for Training and Education<br>De Deyn, Peter Paul; University of Groningen, University Medical Centre Groningen, Neurology and Alzheimer Centre Groningen<br>de Winter, Andrea; University of Groningen, University Medical Centre Groningen, Health Sciences<br>Reesink, Fransje; University of Groningen, University Medical Centre Groningen, Neurology and Alzheimer Centre Groningen<br>Oude Voshaar, Richard; University of Groningen, University Medical Center Groningen, Psychiatry<br>Buskens, Erik; University of Groningen, University Medical Centre Groningen, Epidemiology<br>de Rooij, Sophia; Medical Spectrum Twente, Medical School<br>Smidt, Nynke; University of Groningen, University Medical Centre Groningen, Epidemiology |
| <b>Primary Subject Heading</b>: | Public health |
| Secondary Subject Heading: | Epidemiology, Public health |
| Keywords: | Dementia < NEUROLOGY, Risk management < HEALTH SERVICES ADMINISTRATION & MANAGEMENT, EPIDEMIOLOGY, PREVENTIVE MEDICINE, PUBLIC HEALTH |
| | |

SCHOLARONE™
Manuscripts

*I, the Submitting Author has the right to grant and does grant on behalf of all authors of the Work (as defined in the below author licence), an exclusive licence and/or a non-exclusive licence for contributions from authors who are: i) UK Crown employees; ii) where BMJ has agreed a CC-BY licence shall apply, and/or iii) in accordance with the terms applicable for US Federal Government officers or employees acting as part of their official duties; on a worldwide, perpetual, irrevocable, royalty-free basis to BMJ Publishing Group Ltd ("BMJ") its licensees and where the relevant Journal is co-owned by BMJ to the co-owners of the Journal, to publish the Work in this journal and any other BMJ products and to exploit all rights, as set out in our licence.*

*The Submitting Author accepts and understands that any supply made under these terms is made by BMJ to the Submitting Author unless you are acting as an employee on behalf of your employer or a postgraduate student of an affiliated institution which is paying any applicable article publishing charge ("APC") for Open Access articles. Where the Submitting Author wishes to make the Work available on an Open Access basis (and intends to pay the relevant APC), the terms of reuse of such Open Access shall be governed by a Creative Commons licence – details of these licences and which Creative Commons licence will apply to this Work are set out in our licence referred to above.*

*Other than as permitted in any relevant BMJ Author's Self Archiving Policies, I confirm this Work has not been accepted for publication elsewhere, is not being considered for publication elsewhere and does not duplicate material already published. I confirm all authors consent to publication of this Work and authorise the granting of this licence.*

For peer review only

**TITLE (WORD COUNT: 4525)**

The uptake and effectiveness of a tailor-made online lifestyle program targeting modifiable risk factors for dementia among middle-aged descendants of people with recently diagnosed dementia: study protocol of a cluster randomised controlled trial (Demin study)

**Issue date:** 1 September 2020

**AUTHORS**

Vrijsen J[1]*, Abu-Hanna A[2], Maeckelberghe ELM[3], De Deyn PP[4], de Winter AF[5], Reesink FE[4], Oude Voshaar RC[6], Buskens E[1], de Rooij SEJA[7], Smidt N[1], for the Demin consortium.

*Corresponding author. University Medical Centre Groningen, Department of Epidemiology, Hanzeplein 1, PO Box 30 001, FA40, 9700 RB Groningen, The Netherlands. E-mail: j.vrijsen@umcg.nl; Phone: +31625650782

**Author affiliations**

1 University of Groningen, University Medical Centre Groningen, Department of Epidemiology, Groningen, the Netherlands

2 University of Amsterdam, Amsterdam UMC, Department of Medical Informatics, Amsterdam, the Netherlands

3 University of Groningen, University Medical Centre Groningen, Wenckebach Institute for Training and Education, Groningen, the Netherlands

4 University of Groningen, University Medical Centre Groningen, Department of Neurology and Alzheimer Centre Groningen, Groningen, the Netherlands

5 University of Groningen, University Medical Centre Groningen, Department of Health Sciences, Groningen, the Netherlands

6 University of Groningen, University Medical Centre Groningen, Department of Psychiatry, Groningen, the Netherlands

7 Medical Spectrum Twente, Medical School, Enschede, the Netherlands

For peer review only

**Demin consortium (28)**

Abma EM, Abu-Hanna A, Buskens E, Claassen JAHR, De Deyn PP, Feitsma T , Hempenius L, Hoogmoed J, Kamper A, Koek HL, Kragt JJ, Lagro J, Lambooij SLE, Langedijk M, Maeckelberghe ELM, Mattace Raso FUS, Meinardi MC, Oude Voshaar RC, Reesink FE, de Rooij SEJA, Scheepmaker AJTM, Smidt N, Spies P, Taekema D, Verkuyl J, Vingerhoets R, Vrijsen J, de Winter AF.

For peer review only



For peer review only - http://bmjopen.bmj.com/site/about/guidelines.xhtml

**ABSTRACT**

**Introduction** Descendants of dementia patients have a higher risk to develop dementia. This study aims to investigate the uptake and effectiveness of an online tailor-made lifestyle program for Dementia Risk Reduction (DRR) among middle-aged descendants of people with recently diagnosed late-onset dementia.

**Methods and analysis** Demin is a cluster randomised controlled trial, aiming to include 21 memory clinics of which thirteen will be randomly allocated to the passive (poster and flyer in waiting room) and eight to the active recruitment strategy (additional personal invitation by members of the team of the memory clinic). We aim to recruit 378 participants (40-60 years) with a parent who is recently diagnosed with Alzheimer's Disease or Vascular Dementia at one of the participating memory clinics. All participants receive a dementia risk assessment (online questionnaire, physical examination and blood sample) and subsequently an online tailor-made lifestyle advice regarding protective (Mediterranean diet, low/moderate alcohol consumption, high cognitive activity) and risk factors (physical inactivity, smoking, loneliness, cardiovascular disease, hypertension, high cholesterol, diabetes, obesity, renal dysfunction, depression) for dementia. The primary outcome is the difference in uptake between the two recruitment strategies. Secondary outcomes are change(s) in 1) the Lifestyle for Brain Health (LIBRA) score, 2) individual health behaviours, 3) health beliefs and attitudes towards DRR and 4) compliance to the tailor-made lifestyle advice. Outcomes will be measured at 3, 6, 9 and 12 months after baseline. The effectiveness of this online tailor-made lifestyle program will be evaluated by comparing Demin participants to a matched control group (Lifelines cohort).

**Ethics and dissemination** This study has been approved by the Dutch Ministry of Health, Welfare and Sport according to the Population Screening Act. All participants have to give online informed consent using SMS-tan. Findings will be disseminated through peer-reviewed journals and (inter)national conferences.

**Trial registration number** NTR7434

For peer review only



For peer review only - http://bmjopen.bmj.com/site/about/guidelines.xhtml

## ARTICLE SUMMARY

### Strengths and limitations of this study

- This is the first multicentre trial that focuses on dementia risk reduction in middle-aged descendants of recently diagnosed patients with Alzheimer's disease or Vascular dementia.
- The program gives participants insight in their risk and protective factors for dementia and provides a tailor-made online lifestyle advice with regard to thirteen modifiable risk factors for dementia, taking the stages of (health behaviour) change into account.
- The application ensures the privacy of the participants by using SMS-tan for logging in their personal account and signing the electronic informed consent form.
- The web-based application (demin.nl) functions fully automatically, making it easy to implement the study in other memory clinics and settings.
- Changing health behaviour is difficult and it is unclear whether a tailor-made online lifestyle advice is sufficient to change health behaviour and to maintain a healthy lifestyle.

**KEY WORDS**

Dementia

Health behaviour

Risk reduction behaviour

Lifestyle

Middle aged



For peer review only - http://bmjopen.bmj.com/site/about/guidelines.xhtml

## INTRODUCTION

Dementia is considered a major public health concern [1]. Due to the ageing population the number of dementia cases will increase substantially in the next decades. In 2015, more than 46 million people worldwide were affected by dementia and this number is expected to increase to 131 million by 2050 [2]. This rise in people with dementia carries a high economic and social burden for society [1]. In 2015, global costs of dementia reached 818 billion US dollars and will increase further [3]. Currently, no curative treatments are available. Therefore, prevention is a key element to counteract the dementia epidemic [4,5].

The most common types of dementia are Alzheimer's disease (AD) (60-70%) ,Vascular dementia (VD) (15-20%) or a combination of AD and VD (mixed dementia) [6–8]. The presence of a first-degree relative with AD doubles the risk for developing AD [9]. This increased risk has several reasons. Firstly, descendants of people with AD more often have a higher genetic predisposition for AD (e.g. carrier of the Apo lipoprotein E (APOE) ε4 allele) [9]. Secondly, high blood pressure, vascular diseases and other vascular risk factors (i.e. diabetes type 2, obesity, hypercholesterolemia) often cluster in families [10]. Lastly, psychosocial behaviour runs in the family and also affects health behaviour and lifestyle [11,12]. Not surprisingly, individuals with a parent who is recently diagnosed with AD or VD often worry about their own risk of developing dementia. Therefore, this life event (parental diagnosis of dementia) might encourage the willingness of individuals to change their health behaviour [13].

Parental family history has been associated with an increased risk of dementia independently of known genetic risk factors [9,14]. Therefore, a healthy lifestyle might be beneficial for individuals with a positive family history, especially for APOE ε4 carriers [15–18]. Over the last decade, evidence of modifiable risk factors for dementia has been mounting [4,6,19]. The Lancet commission on dementia prevention, intervention and care demonstrated that 35% of the dementia cases is attributable to modifiable risk factors (i.e. less education, hearing loss, midlife hypertension, midlife obesity, smoking, depression, physical inactivity, social isolation and diabetes) and recommended to start



For peer review only - http://bmjopen.bmj.com/site/about/guidelines.xhtml

interventions including more childhood education, promotion of physical exercise, reduction of smoking, maintaining social engagement and management of hypertension, diabetes, obesity, depression and hearing loss [4,6,20]. Other major risk factors are hyperlipidaemia, coronary heart disease, renal dysfunction, Mediterranean diet and cognitive activity [19].

Only few studies examined the effectiveness of targeting these modifiable factors on cognitive decline and dementia incidence through a multi-domain intervention, such as the (Finnish Geriatric Intervention Study to Prevent Cognitive Impairment and Disability) FINGER study [21], the (Prevention of Dementia by Intensive Vascular care) PreDIVA study [22] and the (The Multi-domain Alzheimer Preventive Trial) MAPT study [23]. These studies, with a follow-up varying from two to six years, found small or non-significant effects on cognition in older participants (e.g. >60 years) [21–23]. Starting multi-domain interventions earlier in life might be promising as cognitive decline begins already in midlife [24,25]. However, since dementia is mainly prevalent in the elderly, a long follow-up period of approximately 20 years would be required in order to determine the effectiveness of interventions on dementia incidence [24–26]. Furthermore, tailoring interventions improves the effectiveness of health behaviour change interventions [27]. Web-based interventions have the potential to support health behaviour change as there is the opportunity to tailor lifestyle advice [28–31]. They were especially effective when a theoretical basis or conceptual framework (e.g. Health belief model (HBM), Trans theoretical model (TTM), Theory of planned behaviour (TPB), I(integrated)-Change model [32–36]), behaviour change techniques (e.g. providing feedback on performance and information on the consequences of unhealthy behaviour) and several modes of delivery had been used [27].

The first challenge of health behaviour change interventions is to achieve a high level of uptake for screening (e.g. assessing risk and protective factors for dementia), reflecting the willingness to participate. A systematic review identified a large variation in uptake in health checks and lifestyle intervention programs [37], depending on the type of recruitment strategy. The two main types of strategies for recruitment are the active and passive recruitment strategy. Active recruitment involves a



For peer review only - http://bmjopen.bmj.com/site/about/guidelines.xhtml

personal invitation by the project staff and healthcare providers (e.g. proactive) and passive recruitment involves recruitment of participants through various channels such as flyers and advertisements (e.g. reactive) [38]. The most effective recruitment strategy is proactive referral from a healthcare provider, while displaying posters and flyers showed to be less effective [39,40]. Uptake also depends on other factors as described in social cognition models (e.g. knowledge, perceived susceptibility and severity, facilitators, benefits and barriers, and attitude towards such interventions) [32–36]. These factors are essential and useful to make a well-informed decision about dementia risk assessment, considering the possible benefits and harms. Therefore, information on dementia, the risk and protective factors for dementia, heritability, and how to tackle risk and protective factors for dementia are important factors in the development of a web-based intervention. A previous study showed that the majority of the Dutch general population is unaware of the relationship between modifiable risk factors and brain health, particularly regarding major cardiovascular risk factors (e.g. hypertension, hypercholesterolemia and coronary heart disease) [41]. It is shown that this lack of knowledge is a barrier to the uptake and maintenance of healthy behaviours for middle-aged individuals [42]. Having a parent who is recently diagnosed with AD or VD could have led to an increased knowledge on dementia and risk perception [13]. Therefore, middle-aged descendants of recently diagnosed people with AD or VD might be receptive to assess their risk and motivated to adopt a healthier lifestyle as they just realized their (familial) risk [13,43]. Although we expect that the uptake in the active recruitment strategy will be higher compared to the passive recruitment strategy, participants recruited via the passive recruitment strategy might be more intrinsically motivated to adopt and maintain their healthy lifestyle and less likely to drop out of the study.

To our knowledge, none of the health behaviour intervention studies were aimed at a specific group of middle-aged adults with increased risk for dementia due to their parental family history of dementia. Therefore, this study aims to investigate the uptake and effectiveness of a tailor-made online lifestyle program for dementia risk reduction among middle-aged descendants of recently diagnosed (in the last six months) people with AD or VD in the Netherlands. This will give insight in to what extent it is feasible to recruit middle-aged descendants of people with AD or VD at the memory clinics and



For peer review only - http://bmjopen.bmj.com/site/about/guidelines.xhtml

whether these potential participants are willing to participate in a tailor-made online lifestyle program in order to reduce their dementia risk.

## METHODS AND ANALYSIS

### Study setting and design

This study is a pragmatic cluster randomized controlled trial (RCT), including 21 participating memory clinics in the Netherlands who are randomly allocated to a passive or active recruitment of participants. Memory clinics allocated to the active recruitment strategy invite potential participants face-to-face by a member of the team of the memory clinic to participate in the tailor-made online lifestyle program for dementia risk reduction (also called the Demin study), next to posters and flyers that are placed in the waiting room of the memory clinic. Memory clinics allocated to the passive recruitment strategy, do not invite potential participants pro-actively, but invite potential participants to participate in the Demin study by posters and flyers that are placed in the waiting room of the memory clinic.

Patients with AD or VD (or their caregivers) receive an envelope either at the registration desk of the memory clinic or after the consult of the patient (only with active recruitment). This envelope is addressed to the middle-aged descendants of patients with recently diagnosed AD or VD and includes a patient information form (PIF) with information about the content of the study, the advantages and disadvantages of study participation and how potential participants can participate. Potential participants (one family member per patient) are asked to register themselves (e.g. making an account) on the Demin website (www.demin.nl), by using the memory clinic specific login access code, which is reported on the front page of the PIF and represents the memory clinic in which the parent was diagnosed. The decision to participate is confirmed by the participants by signing the online informed consent form (electronic signature by using SMS-tan). After signing this form, individuals from both recruitment strategies are able to log in to their personalized website 'My Demin' and continue the intervention in an equal manner. The personalized website 'My Demin' is secured and only accessible for the participant by logging in with their personal e-mail address, password and SMS-tan code. 'My Demin' contains the following information: 1) My personal (account) information, 2) Message inbox,



For peer review only - http://bmjopen.bmj.com/site/about/guidelines.xhtml

3) My online questionnaires, 4) My personal health profile including online tailor-made lifestyle advice. After participants have completed the online questionnaire, they automatically receive a message with a request to make an appointment for physical examination including a fasting blood sample. Moreover, participants can invite siblings to participate in the study in 'My Demin'. The functionalities provided by the Demin website are based on the literature and input we received from people with a parent with dementia (focus group discussions).

**Randomization of memory clinics**

To prevent contamination between the two recruitment strategies, randomization is performed at the level of the memory clinics. To enhance comparability between the intervention (participants of the active recruitment strategy) and control group (participants of the passive recruitment strategy), the memory clinics will be matched and randomised by a statistician, who is blind to the identity of the memory clinics and not involved in the study. Firstly, all participating memory clinics will be matched into pairs based on the following criteria: (i) number of newly diagnosed dementia (VD, AD or mixed dementia) patients seen per year (range vary from 60 to 350 patients per year) and (ii) the average social economic position (SEP) of the population living around the memory clinic (neighbourhood SEP), based on data from Statistics Netherlands [44]. Secondly, the matched memory clinics will be randomized (pairwise randomization) to an active recruitment strategy or passive recruitment strategy using a computer-generated random number list. As we expect a higher response rate in the active recruitment strategy group, we use an active : passive recruitment strategy ratio of 8:13 (see sample size calculations).

**Study population**

Eligible participants are middle-aged individuals (40-60 years old) with a parent who is recently (less than 6 months ago) diagnosed with AD or VD (or mixed dementia) at one of the participating memory clinics in the Netherlands (see acknowledgement). Individuals should provide informed consent, be able to fill out an online Dutch questionnaire. Pregnant women are excluded from participation.



For peer review only - http://bmjopen.bmj.com/site/about/guidelines.xhtml

**Sample size calculations**

The primary outcome measure is uptake, which is defined as the percentage of eligible individuals that signed the online informed consent form and completed baseline assessment (online questionnaire and physical examination and a fasting blood sample). In order to detect a difference of 20% in uptake between the passive and active recruitment strategy (30% versus 50%), we need 94 participants in each group to achieve a power of 80% with alpha levels of 0.05 (total = 188 participants). To take cluster randomization into account, we use the formula 1+ ((n-1)*ICC) (inflation factor), where n is the average number of included participants per memory clinic and the ICC the Intra Class Correlation [45]. The ICC is unknown, but an ICC of 0.05 is a common value for cluster randomized controlled trials in hospitals [46]. The estimated average of included participants per memory clinic per year is n=15 using a passive recruitment strategy and n=25 using an active recruitment strategy, taking into account non-response. With unequal cluster sizes, 'n' is replaced by 'm', where m is the sum of $(M)^2$/sum(M) $((15^2+25^2) / (15+25))$ [47]. This results in a sample size inflation factor of (1+ ((21.25-1)*0.05)= 2.01. Therefore, the total number of participants needed is 378 (2.01 * 188). In order to recruit 378 participants, we need 21 memory clinics, of which eight memory clinics (responsible for 189 included participants) will be allocated to the active recruitment strategy and thirteen memory clinics (responsible for 189 included participants) will be allocated to the passive recruitment strategy.

**Demin website**

The Demin website is available for everyone and provides information about dementia, heredity of dementia, risk and protective factors for dementia, and how to tackle potential risk factors for dementia. The health information will be provided by written text and in an audio-visual format, such as a spoken animation, to assure inclusion of participants with different levels of health literacy [48]. According to the cognitive theory of multimedia learning (CTML), people process visual and auditory information through different channels [49,50]. It is known that health information provided by various channels, such as written text and spoken animations, improves information processing compared to information only provided through written text or spoken animations [49,50]. The



For peer review only - http://bmjopen.bmj.com/site/about/guidelines.xhtml

instructions for registration (making an account, signing informed consent) are also provided as written text as visual screenshots representing the steps of the registration process.

**Online tailor-made lifestyle program for dementia risk reduction**

After participants give online informed consent, participants have access to the online tailor-made lifestyle program for dementia risk reduction, which consists of 1) a dementia risk assessment and 2) an online tailor-made lifestyle advice including a personal health profile targeting risk and protective factors for dementia.

**1. Dementia risk assessment**

The dementia risk assessment consists of filling out an online questionnaire (in 'My Demin') and physical examination, including a fasting blood sample, at one of the 21 participating memory clinics in order to determine whether risk and protective factors are present. In order to minimize the amount of missing data, validation and skip-and-fail rules were implemented in the online questionnaire. Furthermore, automatic reminders are sent to the participant if the online questionnaire was not filled in within two weeks. Physical examination will be conducted by the team of the local memory clinic and includes the following measurements: height (in cm) (SECA 222 stadiometer), body weight (in kg) without shoes (SECA 761 scale), waist- and hip circumference (in cm) (SECA 200 measuring tape), and three measurements of diastolic and systolic blood pressure (in mmHg) (Welch Allyn 'Spot Vital Signs' [51]). After physical examination, which takes approximately 15 minutes, a fasting blood sample (maximum of 21 ml) is taken for direct laboratory measurement of glucose, HbA1C, total cholesterol, High-density-lipoprotein (HDL), Low-density-lipoprotein (LDL), triglycerides and serum creatinine. The results of the physical examination (height, body weight, blood pressure, waist- and hip circumference) are sent to the researcher (J. Vrijsen) to check the entry of the results by the participants. The results of the direct laboratory measurements are sent to the medical doctor (E.M. Abma) of the University Medical Centre Groningen to check for deviating values.

**Risk and protective factors for dementia**



For peer review only - http://bmjopen.bmj.com/site/about/guidelines.xhtml

278 Through the online questionnaire and physical examination, data on thirteen currently known
279 protective (i.e. Mediterranean diet, low/moderate alcohol consumption, cognitive activity) and risk
280 factors (i.e. physical inactivity, smoking, loneliness, cardiovascular diseases, hypertension, high
281 cholesterol, diabetes mellitus, obesity, renal dysfunction, depression) for dementia are collected
282 [6,19,52]. See **Table 1** for an overview of the assessment measures. The measurements of these risk
283 and protective factors are described in **Supplementary file 1**.
284

**Table 1.** Assessment measures at baseline and follow up

| | **Baseline** | **3 months** | **6 months** | **9 months** | **12months** |
|---|---|---|---|---|---|
| **RISK AND PROTECTIVE FACTORS** | | | | | |
| Smoking | Q | Q | Q | Q | Q |
| Physical inactivity (SQUASH, IPAQ) | Q | Q | Q | Q | Q |
| Mediterranean diet (FFQ) | Q | Q | Q | Q | Q |
| Alcohol consumption (FFQ) | Q | Q | Q | Q | Q |
| High cognitive activity (CRIq) | Q | Q | Q | Q | Q |
| Loneliness (de Jong Gierveld, 6-item) | Q | Q | Q | Q | Q |
| Cardiovascular diseases (CVD) | Q | Q | Q | Q | Q |
| Obesity (body weight, height) | Q+PE | Q | Q | Q | Q+PE |
| Hypertension (SBD, DBP) | Q+PE | Q | Q | Q | Q+PE |
| High cholesterol (LDL, HDL, TC) | Q+FBS | Q | Q | Q | Q+FBS |
| Diabetes Mellitus (glucose, HbA1C) | Q+FBS | Q | Q | Q | Q+FBS |
| Renal dysfunction (eGFR) | Q+FBS | Q | Q | Q | Q+FBS |
| Depression (CES-D) | Q | Q | Q | Q | Q |

*SQUASH* Short Questionnaire to Assess Health-enhancing physical activity, *IPAQ* International Physical Activity Questionnaire, *FFQ* Food Frequency Questionnaire, *CRIq* Cognitive Reserve Index questionnaire (adapted), *CVD* Cardiovascular diseases, *SBP* Systolic Blood Pressure, *DBP* Diastolic Blood Pressure, *HDL*



For peer review only - http://bmjopen.bmj.com/site/about/guidelines.xhtml

high-density lipoproteins, *LDL* low-density lipoproteins, *TC* total cholesterol, *HbA1C* Haemoglobin A1C, *eGFR* estimated Glomerular Filtration Rate, *CES-D* Centre for Epidemiological Studies Depression Scale

Q: Online questionnaire, PE: Physical examination, FBS: Fasting blood sample

**2a. Personal health profile**

After completion of the baseline dementia risk assessment (including the data entry of the physical examination and laboratory measurements), a personal health profile is automatically provided in the personal account of the participants (My Demin). The personal health profile gives an overview of the presence of the risk and protective factors for dementia, without including the weight of the risk and protective factors. According to the Lifestyle for Brain Health (LIBRA) score, each risk and protective factor [19,52,53] is categorized into one of the following categories: 1) room for improvement, 2) remember to manage well, 3) keep this up (see **Table 2**). The “Keep this up” category represent factors that participants are currently managing well or diseases they do not have. The “Room for improvement” category represents the factors that could be improved by health behaviour change (e.g. quit smoking, become more physical active, change diet, drink less alcohol). The category “Remember to manage well” is assigned when a risk factor (i.e. cardiovascular disease, hypertension, high cholesterol, diabetes mellitus, renal dysfunction and depression) is present, but the disease is managed well as participants have regular meetings with their general practitioner for disease control (diabetes mellitus) or use medication for disease management (cardiovascular disease, hypertension, high cholesterol, renal dysfunction and depression) (see **Figure 1**).

**[INSERT FIGURE 1 ABOUT HERE]**

For peer review only



For peer review only - http://bmjopen.bmj.com/site/about/guidelines.xhtml

**Table 2.** Definition for the 3 categories in the personal health profile at baseline

| Modifiable risk factors | Keep this up | Remember to manage well | Room for improvement |
|---|---|---|---|
| **Diet** | MIND-diet score = 14 points | n.a. | MIND-diet score < 14 points |
| **Alcohol consumption** | Average number of units of alcohol per week ≤ 7 and number of units per day is: ≤ 3 for women or ≤ 4 for men | n.a. | Average number of units of alcohol per week > 7 or number of units per day is: > 3 for women or > 4 for men |
| **Cognitive activity** | paid working hours ≥ 24 or CRIq score ≥ 50 | n.a. | paid working hours < 24 and CRIq score < 50 |
| **Physical activity** | (MVPA / week ≥ 150 and Sitting time ≤ 8 hours / day) or (MVPA / week < 150 and sitting time < 4 hours / day) | n.a. | (Sitting time > 8 hours / day) or (Sitting time ≥ 4 hours / day and MVPA / week < 150) |
| **Smoking** | Past or never smoker | n.a. | Current smoker |
| **Loneliness** | De Jong Gierveld score < 2 | n.a. | De Jong Gierveld score ≥ 2 |
| **Cardiovascular diseases (CVD)** | no CVD | at least one CVD and receives medical treatment | at least one CVD and no medical treatment |
| **Weight** | BMI ≥ 18.5 and BMI < 25.0 | n.a. | BMI < 18.5 or BMI ≥ 25.0 |



For peer review only - http://bmjopen.bmj.com/site/about/guidelines.xhtml

| | | | |
|---|---|---|---|
| **Blood pressure** | DBP < 90 mmHg and SBP < 140 and no medical treatment | DBP < 90 mmHg and SBP < 140 and medical treatment | DBP ≥ 90 mmHg or SBP ≥ 140 mmHg |
| **Cholesterol** | (LDL ≤ 2.5 mmol/l and TC/HDL ≤ 5) and no medical treatment | (LDL ≤ 2.5 mmol/l and TC/HDL ≤ 5) and medical treatment | LDL > 2.5 mmol/l or TC/HDL > 5 |
| **Diabetes Mellitus** | glucose < 7.0 mmol and HbA1C ≤ 53 mmol/mol | (HbA1C ≤ 53 mmol/mol and medical treatment) or (glucose < 7.0 mmol and HbA1C > 53 mmol/mol and medical treatment) | (HbA1C > 53 mmol/mol and no medical treatment) or (glucose ≥ 7.0 mmol and HbA1C > 53 mmol/mol) or (glucose ≥ 7.0 mmol and HbA1C ≤ 53 mmol/mol and no medical treatment) |
| **Kidney** | eGFR ≥ 60 ml/min/1.73 $m^2$ | eGFR < 60 ml/min/1.73 $m^2$ and medical treatment | eGFR < 60 ml/min/1.73 $m^2$ and no medical treatment |
| **Depression** | CES-D < 16 points | CES-D ≥ 16 points and medical treatment | CES-D ≥ 16 points and no medical treatment |

*MIND-diet* Mediterranean-DASH Diet Intervention for Neurodegenerative Delay, *CRIq* Cognitive Reserve Index questionnaire, *MVPA* Moderate to vigorous physical activity, CVD Cardiovascular diseases, *BMI* Body mass index, *DBP* diastolic blood pressure, *SBP* systolic blood pressure, *LDL* low-



For peer review only - http://bmjopen.bmj.com/site/about/guidelines.xhtml

density lipoproteins, *TC* total cholesterol, *HDL* high-density lipoproteins, *HbA1C* Haemoglobin A1C, *eGFR* estimated Glomerular Filtration Rate, *CES-D* Centre for Epidemiological Studies Depression Scale

For peer review only

**2b. Tailor-made online lifestyle advice for dementia risk reduction**

Participants also receive an online tailor-made lifestyle advice targeting risk factors associated with dementia and following the Dutch guidelines for a healthy diet, alcohol consumption, physical activity, diabetes mellitus, renal dysfunction and cardiovascular health including cholesterol levels and BMI [54–58]. For each risk and protective factor, information is given about (i) the norm (cut-off point for not having this risk factor), (ii) the association between the risk factor and dementia and (iii) lifestyle advice how to tackle this factor. The online lifestyle advice was tailored to the participants based on (i) the presence of risk factors, (ii) the strength of the association between the risk factors and dementia [19,52] and (iii) the stages of change of the health behaviour related risk factors (physical inactivity, diet, alcohol consumption, smoking behaviour, cognitive activity, social activity). The stages of change are determined by asking "Which statement fits best for you?", where each answer option reflects one of the following stages of change: pre-contemplation, contemplation, preparation, action and maintenance [33]. It is known that participants who are in the preparation and action stage are more willing to change their health behaviour, therefore lifestyle advice for these factors are given first [33].

In case medically relevant findings are found, including untreated diabetes mellitus (glucose ≥ 7.0 mmol/l or (glucose ≥ 6.1 mmol/l and HbA1C > 53 mmol/mol)), untreated renal dysfunction (estimated Glomerular Filtration Rate (eGFR) ≤ 60 ml/min/1.73 m2) and increased risk for developing cardiovascular diseases (CVD) (CVD risk ≥ 10% according to the Dutch SCORE formula [58]), participants receive, in addition to the online tailor-made lifestyle advice, a separate message in their personal inbox with the recommendation to contact their general practitioner to verify the results and discuss whether treatment is needed.

**Outcome measures and measurements**

Participants are invited to fill in the online questionnaire at baseline and four times (3, 6, 9 and 12 months after baseline measurement) during one year follow-up. Physical examination, including the fasting blood sample for direct laboratory measurements, is only done at baseline and 12 months after



For peer review only - http://bmjopen.bmj.com/site/about/guidelines.xhtml

baseline measurement (see **Supplementary file 2**). Data from the online questionnaires and physical examination are stored automatically in an electronic Case Report Form (eCRF) data management program, which is only accessible by the researchers involved in this study. Data from the direct laboratory measurement are entered manually in the electronic Case Report Form (eCRF) data management program. Every month, memory clinics are requested to provide information about 1) the number of eligible participants (e.g. new cases of AD and VD), 2) the number of envelopes that are given away, and 3) any difficulties with the recruitment of participants. In order to keep participating memory clinics involved in the study, every three months newsletters are sent around and memory clinics are contacted monthly to evaluate the uptake.

**Primary outcome**

The primary outcome is the difference in uptake (e.g. the percentage of eligible people that signed the online informed consent form and completed risk assessment of the total number of eligible people) between the active and passive recruitment strategy. The total number of eligible people in each recruitment group (active versus passive) are based on the number of new cases of AD or VD in all memory clinics during the recruitment period, assuming an average of one child per dementia patient receiving the envelope with the PIF including a login access number. Due to privacy regulations it is not possible to collect data regarding the reasons for non-participation.

**Secondary outcomes**

Secondary outcomes include:

**1)** The change in Lifestyle for Brain Health (LIBRA) score. The LIBRA score has been validated among individuals in midlife and reflects an individual's potential to reduce their risk on developing late-onset dementia [52]. The LIBRA score consists of twelve currently known protective (i.e. Mediterranean diet, low/moderate alcohol consumption, cognitive activity) and risk factors (i.e. physical inactivity, smoking, cardiovascular diseases, hypertension, high cholesterol, diabetes mellitus, obesity, renal dysfunction, depression) for dementia (13, 14,31) and ranges from -5.9 (low risk for developing dementia) to 12.7 (high risk for developing dementia).



For peer review only - http://bmjopen.bmj.com/site/about/guidelines.xhtml

A one point increase in the LIBRA score is associated with a 19% higher risk for dementia [52,59]. The definitions and corresponding scores for the three protective and ten risk factors for dementia are described in **Table 3**.

**Table 3.** Definition of risk and protective factors for dementia in the LIBRA score and corresponding scores

| Modifiable risk factors | | **Definition** | **Score** |
|---|---|---|---|
| **Protective factors** | | | |
| **1** | High cognitive activity | Score ≥ 50 points on the Cognitive Reserve Index questionnaire (leisure time activities) (CRIq) or hours of paid work ≥ 24 hours | -3.2 |
| **2** | Mediterranean diet | MIND-diet score (0-14) = 14 points | -1.7 |
| **3** | Low/moderate alcohol consumption | Average number of glasses of alcohol a week ≤ 7 and number of glasses a day is:<br>≤ 3 glasses for women (no binge drinking)<br>≤ 4 glasses for men (no binge drinking) | -1.0 |
| **Risk factors** | | | |
| **4** | Cardiovascular diseases (CVD) | Presence of at least one of the follow diseases: history of angina pectoris, myocardial infarction, transient ischemic attacks, stroke or peripheral arterial diseases | +1.0 |
| **5** | Physical inactivity | Not fulfilling Dutch Norm for Physical activity defined as ≥ 150 min/week physical activity of moderate to vigorous intensity, measured with the SQUASH questionnaire | +1.1 |
| **6** | Renal dysfunction | Estimated glomerular filtration rate ≤ 60 ml/min/1.73 | +1.1 |
| **7** | Diabetes Mellitus | Glucose (capillary blood) > 7.0 mmol/l or HbA1c > 53 mmol/mol | +1.3 |
| **8** | High cholesterol | LDL > 2.5 mmol/l or TC/HDL > 5 | +1.4 |
| **9** | Smoking | Current smoker | +1.5 |



For peer review only - http://bmjopen.bmj.com/site/about/guidelines.xhtml

| | | | |
|---|---|---|---|
| **10** | Obesity | BMI ≥ 30 | +1.6 |
| **11** | Hypertension | SBP > 140 mmHg or DBP > 90 mmHg | +1.6 |
| **12** | Depression | Score ≥ 16 points on the Centre for Epidemiologic Studies Depression scale (CES-D) | +2.1 |

*LDL* low-density lipoproteins*, TC* total cholesterol, *HDL* high-density lipoproteins*, BMI* Body mass index, *SBP* systolic blood pressure, *DBP* diastolic blood pressure

**2)** The change in the individual health behaviours, including physical activity (minutes of MVPA per week), diet (MIND-diet score; 0-14), alcohol consumption (number of glasses of alcohol per week), smoking behaviour (current smoker (yes/no) and number of cigarettes/cigars a day), cognitive activity (leisure-time cognitive activity score and number of hours paid work), loneliness (overall loneliness score; 0-6) and social activity (number of contacts per two weeks) and their stage of change over time. The stages of change are categorized into pre-contemplation (1), contemplation (2), preparation (3), action (4) and maintenance (5) [33].

**3)** Changes in beliefs and attitudes with regard to dementia risk reduction are measured using the Motivation to Change Lifestyle and Health Behaviour for Dementia Risk Reduction Scale (MCLHB-DRR scale) [60,61]. The MCLHB-DRR scale is based on the Health Belief Model [32], which explains health-related behaviours. Seven subscales of the Health Belief Model were included: perceived susceptibility, perceived severity, perceived benefits, perceives barriers, cues to action, general health motivation and self-efficacy. Participants are asked to rate all items on a 5-point Likert scale, ranging from strongly disagree (score=1) to strongly agree (score=5). A higher score on each subscale reflects a higher motivation to change their lifestyle and health behaviour for dementia risk reduction. The Dutch version of the MCLHB-DRR scale, consisting of 23 items, has shown to be valid in the Dutch general population aged between 30 and 80 years old [62].

**4)** Percentage of participants that indicated in the questionnaire that they have followed up the tailor-made online lifestyle advice ("On what risk factors did you receive lifestyle advice?" and "Did you

For peer review only



For peer review only - http://bmjopen.bmj.com/site/about/guidelines.xhtml

follow up the tailor-made lifestyle advice since the last questionnaire (with regard to [risk factor])"? , but also the percentage of participants that indicated that they have followed up the advice to consult their General Practitioner ("Did you have contact with your general practitioner after receiving feedback on the risk and protective factors?").

**Statistical analyses**

First, descriptive characteristics will be explored. The difference in uptake between the two recruitment strategies will be examined using multilevel logistic regression analyses in order to correct for clustering at memory clinic level. We will calculate the percentage with the corresponding 95% confidence interval (CI) and use an alpha of 0.05 to test statistical significance.

The effectiveness of the online tailor-made lifestyle program for dementia risk reduction will be determined by, firstly comparing the change in LIBRA score, the individual risk factors and the MCLHB-DRR score between the active and passive recruitment strategy, and secondly comparing participants of the Demin study (active and passive recruitment strategy) to a control group consisting of Lifelines participants (large population-based cohort study (n > 167.000)) (www.lifelines.nl)[63] in outcome. Lifelines participants (age 40 – 60 years) with a parent with dementia will be matched (using propensity scores) on non-modifiable risk factors (age, gender and education) for dementia to participants of the Demin. Subsequently, multilevel analyses will be performed to examine the change in the LIBRA score and the individual health behaviours over time. In addition, possible confounding and interaction effects will be identified and corrected for in the analysis (e.g. health literacy). We will calculate relative risks (RR) with 95% confidence intervals (CI) and use an alpha of 0.05 to test significance.

**Adverse events**

The risk classification of this intervention is considered negligible, since only information and health advice is provided. Serious adverse events as a result of the intervention are not expected, thus no data safety and monitoring board is installed. Potential participants are informed about possible adverse events. For example dementia risk assessment may help raising the awareness of their susceptibility in



For peer review only - http://bmjopen.bmj.com/site/about/guidelines.xhtml

order to motivate health behaviour change [32], however risk assessment could also have an unfavourable effect. Participants may become anxious about developing dementia and could experience more stress if they receive their health profile. Therefore, participants are clearly informed that the presence or absence of risk and protective factors is not a reassurance that they will develop dementia later in life. Furthermore, participants are informed that there is the possibility that unexpected medical findings can be found. In this case, participants receive a separate message in their personal inbox with the recommendation to contact their general practitioner to verify the results (hypertension, high cholesterol, renal dysfunction, diabetes) and discuss whether treatment is needed. Participants may consider online risk assessment as a privacy risk. In this study, all personal information is kept separately from the research data, and participants use a SMS-tan code to login in their personal account.

**Patient and Public Involvement**

Descendants of people with dementia were involved in the development of the Demin website. We assessed the knowledge, beliefs and attitudes towards dementia and dementia risk reduction among descendants of people with dementia (focus group discussions). The results of the focus group discussions were used to develop the Demin website in order to improve the participant recruitment and encourage health behaviour change among participants.

**Ethics and dissemination**

This study is approved by the Dutch ministry of Health, Welfare and Sport according to the Dutch Population Screening Act. Research which is considered to be Population Screening on the ground of the Population Screening Act, for which ministerial approval is required, does not have to be assessed on the basis of the Medical Research Involving Human Subjects Act [64]. Population screening is defined as ‘medical research in persons carried out on an entire population or a category thereof aimed at the detection of certain types of disease or certain risk indicators for the benefit of the participating subjects’[65]. This project focuses on the attenuations of risk factors for dementia. Since these risk factors are merely lifestyle factors, a positive impact beyond dementia may be expected. Due to a



For peer review only - http://bmjopen.bmj.com/site/about/guidelines.xhtml

healthy lifestyle more healthy life years are added to people’s lives, which may ultimately increase the risk on dementia as age is an important risk factor for dementia. This research is conducted in accordance to the international ethical guidelines [66].

All participants give informed consent to participate in this study, by signing an electronic informed consort form using SMS-tan (see **Supplementary file 3**). Authorship will be allocated using the guidelines for authorship defined by the International Committees of Medical Journal Editors (ICMJE) [67]. The results of the trial will be submitted to an international peer-reviewed journal and presented at national and international conferences.

**Acknowledgements** The authors would like to thank the developers of the Demin website (Bruna&Bruna, Rocket Industries, Centric and Research Data Support from the University Medical Centre Groningen) to make this research possible and the Board of Directors and the collaborators of the participating memory clinics for the local approval and collaboration to conduct this multicentre study: Albert Schweitzer hospital (Dordrecht), Gelre hospital (Apeldoorn), University Medical Centre Groningen (Groningen), Medical Centre Leeuwarden (Leeuwarden), Nij Smellinghe (Drachten), Isala Zwolle (Zwolle), Martini hospital (Groningen),  Haga hospital (Den Haag), Scheper hospital (Emmen), Refaja hospital (Stadskanaal), St. Jans Gasthuis (Weert), University Medical Centre Utrecht (Utrecht), Reinier de Graaf Gasthuis (Delft), Maxima Medical Centre (Eindhoven), Radboud University Medical Centre (Nijmegen), TweeSteden hospital (Tilburg), Erasmus Medical Centre (Rotterdam), Ommelanden Hospital Groningen (Scheemda), Rijnstate hospital (Arnhem and Zevenaar).

For peer review only

**Collaborators** The members of the Demin consortium are: Elske Marije Abma (MD), Ameen Abu-Hanna (PhD), Erik Buskens (MD, PhD), Jürgen Claassen (MD, PhD), Peter Paul De Deyn (MD, PhD), Theo Feitsma (MD), Liesbeth Hempenius (MD, PhD), Jan Hoogmoed (MD), Ad Kamper (MD, PhD), Dineke Koek (MD, PhD), Jolijn Kragt (MD, PhD), Joep Lagro (MD, PhD), Els Lambooij (MD), Marc Langedijk (MD), Els Maeckelberghe (PhD), Francesco Mattace Raso (MD, PhD), Marieke Meinardi (MD, PhD), Richard Oude Voshaar (MD, PhD), Fransje Reesink (MD, PhD), Sophia de Rooij (MD,



For peer review only - http://bmjopen.bmj.com/site/about/guidelines.xhtml

PhD), Antoinette Scheepmaker (MD), Nynke Smidt (PhD), Petra Spies (MD), Diana Taekema (MD, PhD), Jos Verkuyl (MD), Ralf Vingerhoets (MD), Joyce Vrijsen (MSc), Andrea de Winter (PhD).

**Author contributions** JV contributed to the study concept and design, drafting of the manuscript and critical revision of the manuscript. NS and SR conceived the idea, were responsible for data acquisition, contributed to the study concept and design, and the critical revision of the manuscript. AAH, EM, PD, AW, FR, ROV, and EB contributed to the study concept and design, and the critical revision of the manuscript. All authors read and approved the final manuscript.

**Funding** This study was supported by grants from the Netherlands Organisation for Health Research and Development (ZonMw), subprogram prevention program (project number: 531002008).

**Competing interests** None declared

**Data availability** The data collected during this study will be available from the corresponding author upon reasonable request. This study aims to include data from 378 middle-aged participants recruited through participating memory clinics in the Netherlands. The final dataset will include data on physical examination, laboratory data from fasting blood samples and self-reported data including demographic characteristics, health and health behaviour. We will make the data and associated documentation available to users conditional on a data-sharing agreement that provides for: (1) a commitment to using the data only for research purposes, (2) a commitment to securing the data using appropriate computer technology, and (3) a commitment to destroying or returning the data after analyses are completed.

**Patient consent** Obligatory

**Ethics approval** This study is approved by the Dutch ministry of Health, Welfare and Sport according to the population screening act. In addition, all participating memory clinics approved the study.

**Supplementary files**

Supplementary file 1 Measures Dementia Risk Assessment

Supplementary file 2 Overview of measurements at baseline and follow up

Supplementary file 3 Consent form model

For peer review only



For peer review only - http://bmjopen.bmj.com/site/about/guidelines.xhtml

## REFERENCES


1 World Health Organization. Dementia: a public health priority. *World Heal Organ* 2012;:112.

2 Prince M, Wimo A, Guerchet M, *et al.* World Alzheimer Report 2015: The Global Impact of Dementia - An analysis of prevalence, incidence, cost and trends. *Alzheimer's Dis Int* 2015;:84. doi:10.1111/j.0963-7214.2004.00293.x

3 Wimo A, Guerchet M, Ali G-C, *et al.* The worldwide costs of dementia 2015 and comparisons with 2010. *Alzheimer's Dement* 2017;**13**:1–7. doi:10.1016/j.jalz.2016.07.150

4 Norton S, Matthews FE, Barnes DE, *et al.* Potential for primary prevention of Alzheimer's disease: an analysis of population-based data. *Lancet Neurol* 2014;**13**:788–94. doi:10.1016/S1474-4422(14)70136-X

5 Sindi S, Mangialasche F, Kivipelto M. Advances in the prevention of Alzheimer's Disease. *F1000Prime Rep* 2015;**7**:50. doi:10.12703/P7-50

6 Livingston G, Sommerlad A, Orgeta V, *et al.* The Lancet Commissions Dementia prevention, intervention, and care. Published Online First: 2017. doi:10.1016/S0140-6736(17)31363-6

7 Fratiglioni L, Launer LJ, Andersen K, *et al.* Incidence of dementia and major subtypes in Europe: A collaborative study of population-based cohorts. *Neurology* 2000;**54**:1–10.

8 Rizzi L, Rosset I, Roriz-Cruz M. Global Epidemiology of Dementia: Alzheimer's and Vascular Types. *Biomed Res Int* 2014;**2014**:1–8. doi:10.1155/2014/908915

9 Scarabino D, Gambina G, Broggio E, *et al.* Influence of family history of dementia in the development and progression of late-onset Alzheimer's disease. *Am J Med Genet Part B Neuropsychiatr Genet* 2016;**171**:250–6. doi:10.1002/ajmg.b.32399

10 van Exel E, Eikelenboom P, Comijs H, *et al.* Vascular factors and markers of inflammation in offspring with a parental history of late-onset Alzheimer disease. *Arch Gen Psychiatry* 2009;**66**:1263–70. doi:10.1001/archgenpsychiatry.2009.146

11 Muñoz M, Pong-Wong R, Canela-Xandri O, *et al.* Evaluating the contribution of genetics and familial shared environment to common disease using the UK Biobank. *Nat Genet* 2016;**48**:980–3. doi:10.1038/ng.3618

12 Borenstein AR, Copenhaver CI, Mortimer JA. Early-Life Risk Factors for Alzheimer Disease.


For peer review only



For peer review only - http://bmjopen.bmj.com/site/about/guidelines.xhtml

*Alzheimer Dis Assoc Disord* 2006;**20**:63–72. doi:10.1097/01.wad.0000201854.62116.d7

13 Andersson L, Stanich J. Life events and their impact on health attitudes and health behavior. *Arch Gerontol Geriatr* 1996;**23**:163–77. doi:10.1016/0167-4943(96)00716-9

14 Wolters FJ, van der Lee SJ, Koudstaal PJ, *et al.* Parental family history of dementia in relation to subclinical brain disease and dementia risk. *Neurology* 2017;**88**:1642–9. doi:10.1212/WNL.0000000000003871

15 Licher S, Ahmad S, Karamujić-Čomić H, *et al.* Genetic predisposition, modifiable-risk-factor profile and long-term dementia risk in the general population. *Nat Med* 2019;**25**:1364–9. doi:10.1038/s41591-019-0547-7

16 Lourida I, Hannon E, Littlejohns TJ, *et al.* Association of Lifestyle and Genetic Risk With Incidence of Dementia. *JAMA* 2019;**322**:430. doi:10.1001/jama.2019.9879

17 Dekhtyar S, Marseglia A, Xu W, *et al.* Genetic risk of dementia mitigated by cognitive reserve: A cohort study. *Ann Neurol* 2019;**86**:68–78. doi:10.1002/ana.25501

18 Kivipelto M, Rovio S, Ngandu T, *et al.* Apolipoprotein E epsilon4 magnifies lifestyle risks for dementia: a population-based study. *J Cell Mol Med* 2008;**12**:2762–71. doi:10.1111/j.1582-4934.2008.00296.x

19 Deckers K, van Boxtel MPJ, Schiepers OJG, *et al.* Target risk factors for dementia prevention: a systematic review and Delphi consensus study on the evidence from observational studies. *Int J Geriatr Psychiatry* 2015;**30**:234–46. doi:10.1002/gps.4245

20 de Bruijn RF, Bos MJ, Portegies ML, *et al.* The potential for prevention of dementia across two decades: the prospective, population-based Rotterdam Study. *BMC Med* 2015;**13**:132. doi:10.1186/s12916-015-0377-5

21 Ngandu T, Lehtisalo J, Solomon A, *et al.* A 2 year multidomain intervention of diet, exercise, cognitive training, and vascular risk monitoring versus control to prevent cognitive decline in at-risk elderly people (FINGER): a randomised controlled trial. *Lancet (London, England)* 2015;**385**:2255–63. doi:10.1016/S0140-6736(15)60461-5

22 van Charante EPM, Richard E, Eurelings LS, *et al.* Effectiveness of a 6-year multidomain vascular care intervention to prevent dementia (preDIVA): a cluster-randomised controlled trial. *Lancet*

For peer review only



For peer review only - http://bmjopen.bmj.com/site/about/guidelines.xhtml

2016;**388**:797–805. doi:10.1016/S0140-6736(16)30950-3

23 Vellas B, Carrie I, Gillette-Guyonnet S, *et al.* MAPT Study: A Multidomain Approach for Preventing Alzheimer's Disease: Design and Baseline Data. *J Prev Alzheimer's Dis* 2014;**1**:13–22.

24 Singh-Manoux A, Kivimaki M, Glymour MM, *et al.* Timing of onset of cognitive decline: results from Whitehall II prospective cohort study. *BMJ* 2012;**344**:d7622. doi:10.1136/BMJ.D7622

25 Sperling RA, Aisen PS, Beckett LA, *et al.* Toward defining the preclinical stages of Alzheimer's disease: recommendations from the National Institute on Aging-Alzheimer's Association workgroups on diagnostic guidelines for Alzheimer's disease. *Alzheimers Dement* 2011;**7**:280–92. doi:10.1016/j.jalz.2011.03.003

26 Rajan KB, Wilson RS, Weuve J, *et al.* Cognitive impairment 18 years before clinical diagnosis of Alzheimer disease dementia. *Neurology* 2015;**85**:898–904. doi:10.1212/WNL.0000000000001774

27 Webb TL, Joseph J, Yardley L, *et al.* Using the internet to promote health behavior change: a systematic review and meta-analysis of the impact of theoretical basis, use of behavior change techniques, and mode of delivery on efficacy. *J Med Internet Res* 2010;**12**:e4. doi:10.2196/jmir.1376

28 van Stralen MM, de Vries H, Mudde AN, *et al.* The long-term efficacy of two computer-tailored physical activity interventions for older adults: main effects and mediators. *Health Psychol* 2011;**30**:442–52. doi:10.1037/a0023579

29 Schulz DN, Kremers SPJ, Vandelanotte C, *et al.* Effects of a web-based tailored multiple-lifestyle intervention for adults: a two-year randomized controlled trial comparing sequential and simultaneous delivery modes. *J Med Internet Res* 2014;**16**:e26. doi:10.2196/jmir.3094

30 Jahangiry L, Montazeri A, Najafi M, *et al.* An interactive web-based intervention on nutritional status, physical activity and health-related quality of life in patient with metabolic syndrome: a randomized-controlled trial (The Red Ruby Study). *Nutr Diabetes* 2017;**7**:e240. doi:10.1038/nutd.2016.35

31 Murray E. Web-based interventions for behavior change and self-management: potential, pitfalls,

For peer review only

and progress. *Med 20* 2012;**1**:e3. doi:10.2196/med20.1741

32 Janz NK, Becker MH. The Health Belief Model: A Decade Later. *Heal Educ Behav* 1984;**11**:1–47. doi:10.1177/109019818401100101

33 Prochaska JO, Velicer WF. The transtheoretical model of health behavior change. *Am J Health Promot* 1997;**12**:38–48.

34 Hagger MS, Chatzisarantis NLD. Integrating the theory of planned behaviour and self-determination theory in health behaviour: A meta-analysis. *Br J Health Psychol* 2009;**14**:275–302. doi:10.1348/135910708X373959

35 Bandura A. Social Cognitive Theory: An Agentic Perspective. *Annu Rev Psychol* 2001;**52**:1–26. doi:10.1146/annurev.psych.52.1.1

36 de Vries H. An Integrated Approach for Understanding Health Behavior; The I-Change Model as an Example. *Psychol Behav Sci Int J* 2017;**2**. doi:10.19080/pbsij.2017.02.555585

37 Koopmans B, Nielen MM, Schellevis FG, *et al.* Non-participation in population-based disease prevention programs in general practice. *BMC Public Health* 2012;**12**:856. doi:10.1186/1471-2458-12-856

38 Yancey AK, Ortega AN, Kumanyika SK. EFFECTIVE RECRUITMENT AND RETENTION OF MINORITY RESEARCH PARTICIPANTS. *Annu Rev Public Health* 2006;**27**:1–28. doi:10.1146/annurev.publhealth.27.021405.102113

39 Bracken K, Askie L, Keech AC, *et al.* Recruitment strategies in randomised controlled trials of men aged 50 years and older: a systematic review. *BMJ Open* 2019;**9**. doi:10.1136/BMJOPEN-2018-025580

40 Van Der Meer V, Nielen MM, Drenthen AJ, *et al.* Cardiometabolic prevention consultation in the Netherlands: screening uptake and detection of cardiometabolic risk factors and diseases – a pilot study. *BMC Fam Pract* 2013;**14**:1. doi:10.1186/1471-2296-14-29

41 Heger I, Deckers K, van Boxtel M, *et al.* Dementia awareness and risk perception in middle-aged and older individuals: baseline results of the MijnBreincoach survey on the association between lifestyle and brain health. *BMC Public Health* 2019;**19**:678. doi:10.1186/s12889-019-7010-z

42 Kelly S, Martin S, Kuhn I, *et al.* Barriers and facilitators to the uptake and maintenance of healthy

For peer review only



For peer review only - http://bmjopen.bmj.com/site/about/guidelines.xhtml

behaviours by people at mid-life: A rapid systematic review. PLoS One. 2016;**11**. doi:10.1371/journal.pone.0145074

43 Rosenberg A, Coley N, Soulier A, *et al.* Experiences of dementia and attitude towards prevention: a qualitative study among older adults participating in a prevention trial. *BMC Geriatr* 2020;**20**:99. doi:10.1186/s12877-020-1493-4

44 Centraal Bureau voor de Statistiek. Percentage hoger opgeleiden van 15 laar en ouder - Bevolkingskern (2011). 2011.http://www.cbsinuwbuurt.nl/#bevolkingskern2011_percentage_inwoners_hogere_opleiding (accessed 18 Feb 2019).

45 DONNER A, BIRKETT N, BUCK C. RANDOMIZATION BY CLUSTER. *Am J Epidemiol* 1981;**114**:906–14. doi:10.1093/oxfordjournals.aje.a113261

46 Kul S, Vanhaecht K, Panella M. Intraclass correlation coefficients for cluster randomized trials in care pathways and usual care: hospital treatment for heart failure. *BMC Health Serv Res* 2014;**14**:84. doi:10.1186/1472-6963-14-84

47 Rutterford C, Copas A, Eldridge S. Methods for sample size determination in cluster randomized trials. *Int J Epidemiol* 2015;**44**:1051–67. doi:10.1093/ije/dyv113

48 Meppelink CS, Weert JC van, Haven CJ, *et al.* The Effectiveness of Health Animations in Audiences With Different Health Literacy Levels: An Experimental Study. *J Med Internet Res* 2015;**17**. doi:10.2196/jmir.3979

49 Mayer R. Multimedia Learning. In: Ross BH, ed. *The psychology of learning and motivation*. San Diego: : Academic Press 2002. 85–139.

50 Paivio A. *Mental representations : a dual coding approach*. Oxford University Press 1986.

51 Jones CR, Taylor K, Poston L, *et al.* Validation of the Welch Allyn 'Vital Signs' oscillometric blood pressure monitor. *J Hum Hypertens* 2001;**15**:191–5. doi:10.1038/sj.jhh.1001141

52 Schiepers OJG, Köhler S, Deckers K, *et al.* Lifestyle for Brain Health (LIBRA): a new model for dementia prevention. *Int J Geriatr Psychiatry* Published Online First: February 2017. doi:10.1002/gps.4700

53 Vos SJB, van Boxtel MPJ, Schiepers OJG, *et al.* Modifiable Risk Factors for Prevention of



For peer review only

For peer review only - http://bmjopen.bmj.com/site/about/guidelines.xhtml

Dementia in Midlife, Late Life and the Oldest-Old: Validation of the LIBRA Index. *J Alzheimer's Dis* 2017;**58**:537–47. doi:10.3233/JAD-161208

54 De Grauw W, De Leest K, Schenk P, *et al.* NHG-Standaard Chronische Nierschade. *TPO - Prakt* 2018;**13**:26–9. doi:10.1007/s12503-018-0065-y

55 Gezondheidsraad. Richtlijnen goede voeding 2015. Den Haag: 2015.

56 Gezondheidsraad. Beweegrichtlijnen 2017. Den Haag: 2017.

57 Rutten G, De Grauw W, Nijpels G, *et al.* NHG-Standaard Diabetes mellitus type 2. 2013.

58 Nederlandse Huisartsen Genootschap. Praktische handleiding bij de NHG-Standaard CVRM (2019). 2019.

59 Deckers K, Köhler S, van Boxtel M, *et al.* Lack of associations between modifiable risk factors and dementia in the very old: findings from the Cambridge City over-75s cohort study. *Aging Ment Health* 2017;:1–7. doi:10.1080/13607863.2017.1280767

60 Kim S, Sargent-Cox K, Cherbuin N, *et al.* Development of the motivation to change lifestyle and health behaviours for dementia risk reduction scale. *Dement Geriatr Cogn Dis Extra* 2014;**4**:172–83. doi:10.1159/000362228

61 Beaton DE, Bombardier C, Guillemin F, *et al.* Guidelines for the Process of Cross-Cultural Adaptation of Self-Report Measures. *Spine (Phila Pa 1976)* 2000;**25**:3186–91. doi:10.1097/00007632-200012150-00014

62 Joxhorst T, Vrijsen J, Niebuur J, *et al.* Cross-cultural validation of the motivation to change lifestyle and health behaviours for dementia risk reduction scale in the Dutch general population. *BMC Public Health* 2020;**20**:788. doi:10.1186/s12889-020-08737-y

63 Scholtens S, Smidt N, Swertz MA, *et al.* Cohort Profile: LifeLines, a three-generation cohort study and biobank. *Int J Epidemiol* 2015;**44**:1172–80. doi:10.1093/ije/dyu229

64 Central Committee on Research Involving Human Subjects. Population Screening Act. 2020.https://english.ccmo.nl/investigators/legal-framework-for-medical-scientific-research/laws/population-screening-act (accessed 18 Jun 2020).

65 Central Committee on Research Involving Human Subjects. The definition of population screening. 2020.https://english.ccmo.nl/investigators/types-of-research/other-types-of-

For peer review only



For peer review only - http://bmjopen.bmj.com/site/about/guidelines.xhtml

668 research/population-research/definition (accessed 24 Jun 2020).

669 66 Council for International Organizations of Medical Sciences. International Ethical Guidelines for

670 Biomedical Research Involving Human Subjects. *Bull Med Ethics* 2002;**182**:17–23.

671 67 Editorial A. Recommendations for the Conduct, Reporting, Editing and Publication of Scholarly

672 Work in Medical Journals. *Curr Pediatr* 2017;**16**:90–106. doi:10.15690/vsp.v16i2.1710

For peer review only

**Figure legends**

**Figure 1.** An example of a personal health profile.

For peer review only



For peer review only - http://bmjopen.bmj.com/site/about/guidelines.xhtml

**License statement** I, the Submitting Author has the right to grant and does grant on behalf of all authors of the Work (as defined in the below author licence), an exclusive licence and/or a non-exclusive licence for contributions from authors who are: i) UK Crown employees; ii) where BMJ has agreed a CC-BY licence shall apply, and/or iii) in accordance with the terms applicable for US Federal Government officers or employees acting as part of their official duties; on a worldwide, perpetual, irrevocable, royalty-free basis to BMJ Publishing Group Ltd ("BMJ") its licensees and where the relevant Journal is co-owned by BMJ to the co-owners of the Journal, to publish the Work in BMJ Open and any other BMJ products and to exploit all rights, as set out in our licence.

The Submitting Author accepts and understands that any supply made under these terms is made by BMJ to the Submitting Author unless you are acting as an employee on behalf of your employer or a postgraduate student of an affiliated institution which is paying any applicable article publishing charge ("APC") for Open Access articles. Where the Submitting Author wishes to make the Work available on an Open Access basis (and intends to pay the relevant APC), the terms of reuse of such Open Access shall be governed by a Creative Commons licence – details of these licences and which Creative Commons licence will apply to this Work are set out in our licence referred to above.



For peer review only - http://bmjopen.bmj.com/site/about/guidelines.xhtml

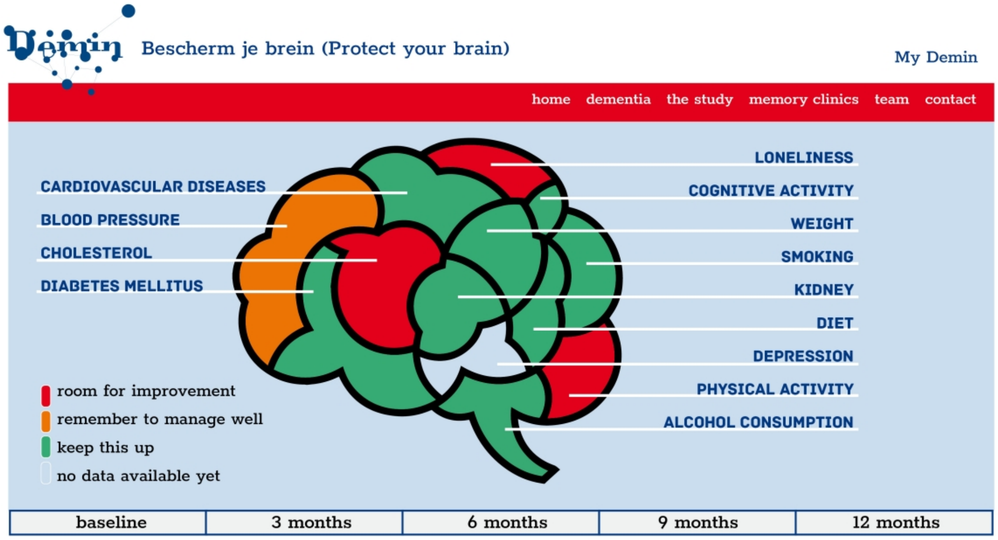


Figure 1. Example of a personal health profile

291x158mm (300 x 300 DPI)

**Supplementary file 1: Measures Dementia Risk Assessment**

Through the online questionnaire and physical examination, data on thirteen currently known protective (i.e. Mediterranean diet, low/moderate alcohol consumption, cognitive activity) and risk factors (i.e. physical inactivity, smoking, loneliness, cardiovascular diseases, hypertension, high cholesterol, diabetes mellitus, obesity, renal dysfunction, depression) for dementia are collected (6,14,41). The measurements of these risk and protective factors are described below.

**Protective factors**

**Mediterranean diet**

The Mediterranean-DASH diet intervention for neurodegenerative delay (MIND) has shown to slow down cognitive decline (1) and to decrease the risk of developing AD (2). Therefore, adherence to the MIND-diet is determined with a number of items of the Food Frequency Questionnaire (FFQ), which is a reliable and valid instrument to measure intake of a specified list of food items in the general populations (3,4). The following healthy food groups of the MIND-diet were included in the questionnaire, such as vegetables (especially green leafy vegetables), nuts, berries, beans, whole grains, seafood, poultry, olive oil (1,2). Also five unhealthy food groups of the MIND-diet including red meat, butter, cheese, sweets and fried/fast food were asked (1,2). Based on the intake of the food groups, adherence to the MIND-diet is determined (0-14). A score of 14 represent good adherence to the MIND-diet *(See **Table 1** for the MIND-diet scoring table).*

For peer review only

**Table 1.** MIND-diet scoring table (2)

| MIND components | Recommended quantity | Max score |
|---|---|---|
| Whole grains | ≥ 3 serving spoons / day | 1 |
| Green leafy | ≥ 6 serving spoons / week | 1 |
| Other vegetables | ≥ 1 serving spoon / day | 1 |
| Berries (including other fruits) | ≥ 2 portions / week * | 1 |
| Red Meats and products | < 4 portions / week | 1 |

| | | |
|---|---|---|
| Fish | ≥ 1 portion / week | 1 |
| Poultry | ≥ 2 portions / week | 1 |
| Beans | > 3 serving spoons /week | 1 |
| Nuts | ≥ 5 portions /week * | 1 |
| Fast/ fried food | < 1time / week | 1 |
| Butter, margarine | < 1 teaspoon/ day | 1 |
| Cheese | < 1 slice / week | 1 |
| Pastries, sweets | < 5 portions / week | 1 |
| Olive Oil (used as primary oil) | yes | 1 |
| **Total score** | | **14** |

** One portion is a handful of the given component*

**Low/moderate alcohol consumption**

Alcohol consumption was measured using the FFQ (5), including questions regarding the frequency of alcohol use (e.g. no consumption last month, 1 day per month, 2-3 days per month, 1 day per week, 2-3 days per week, 4-5 days per week, 6-7 days per week) and the average number of glasses of alcohol per day (range from zero to more than twelve) was asked. Subsequently, the average number of glasses per month was calculated in order to classify participants into: (i) non-alcohol consumers, (ii) low/moderate alcohol consumers or (iii) excessive alcohol consumers (6). Participants adhere to the national recommendations for no to low/moderate alcohol consumption, if participants drink one glass or less alcohol per day on average, without binge drinking (more than three glasses alcohol on one day for females and more than four glasses alcohol on one day for males)) (7).

**High cognitive activity**

Cognitive activity is assessed with the leisure time section of the Cognitive Reserve Index questionnaire (CRIq) (22). CRIq aims to measure cognitive reserve (CR), which is based on education, working activity and leisure time activity. For this study we are interested in the current cognitive

For peer review only

activities of the participants. Therefore, cognitive activity is determined by measuring working activity and leisure time activity. The frequency of eighteen leisure time activity is asked (e.g. (i) never, (ii) less than once a month, (iii) once a month, (iv) once every 2 weeks, (v) several times a week). Subsequently, a leisure time cognitive activity score is calculated, ranging from to 18 to 108, where a score of 50 or higher represent high cognitive activity (based on results of a survey on the knowledge, beliefs and attitudes towards dementia risk reduction among the general population of Groningen, see Table 2 and 3).

Additionally, participants are asked if they have a paid job and if so how many hours they spend on their job per week. High cognitive activity is defined as (i) working at least 24 hours per week or (ii) a leisure time cognitive activity score of at least 50.

**Table 2.** Cognitive activity (leisure time) scores stratified for education level and having a paid job in a survey conducted among the general population in Groningen

| Education level | Work | Leisure time score | |
|---|---|---|---|
| | | mean(SD) | (min-max) |
| Low (n=105) | no work (n=75) | 39.57 (11.16) | 13 – 63 |
| | work (n=30) | 41.73 (11.12) | 25 – 68 |
| Middle (n=154) | no work (n=72) | 47.03 (9.95) | 26 – 76 |
| | work (n=82) | 45.20 (9.49) | 25 – 64 |
| High (n=390) | no work (n= 135) | 51.93 (10.19) | 18 – 75 |
| | work (n=255) | 48.32 (8.97) | 23 – 74 |

**Table 3.** Cognitive activity (leisure time) scores stratified for education level and having a paid job in a survey conducted among the general population in Groningen (subgroup: 40 – 60 year old)

| Education level | Work | Leisure time score | |
|---|---|---|---|
| | | mean(SD) | (min-max) |

For peer review only

| | | | |
|---|---|---|---|
| Low (n=29) | no work (n=7) | 39.71 (9.67) | 21 – 49 |
| | work (n=22) | 40.50 (10.52) | 25 – 58 |
| Middle (n=68) | no work (n=9) | 43. 89 (13.15) | 26 – 66 |
| | work (n=59) | 46.34 (8.87) | 25 – 64 |
| High (n=140) | no work (n= 16) | 50.56 (10.59) | 37 – 69 |
| | work (n=124) | 49.91 (9.24) | 23 – 74 |

For peer review only

**Risk factors**

**Physical inactivity**

Physical activity levels are determined using the Short Questionnaire to Asses Health enhancing physical activity (SQUASH), a self-reported questionnaire and commonly used instrument in the Netherlands to assess physical activity (8). The SQUASH questionnaire has shown to be valid and reliable in measuring physical activity among the Dutch population (9–12). The SQUASH questionnaire includes questions on multiple activities referring to an average week in the last month, including actively commuting (walking, cycling) to (voluntary) work or school, physical activity at (voluntary) work or school, household activities and leisure time activities, including walking, cycling, gardening and sports. Participants were asked to fill in how many days a week they engaged in the activities (frequency), the average time per day spent on each activity (hours and minutes; duration) and the intensity at which they did the activity (low, moderate, high) (8). A standardized methodology was followed to calculate physical activity levels. Briefly, results from the SQUASH questionnaire are automatically converted to minutes per week spent in light (LPA) and moderate to vigorous (MVPA) intensity activities based on Metabolic Equivalent Tasks (METs) derived from the Ainsworth's compendium of physical activity (13). Physical activity levels are divided into the following categories: 0 minutes MVPA per week, 0 to 149 minutes MVPA per week, 150 to 299 minutes MVPA per week and 300 minutes MVPA per week and more. Physical inactivity is defined as less than 150 minutes per week MVPA (14).

Additionally, the questionnaire contained information on sitting behaviour, which is divided into sitting during transportation, working hours, watching television or using the computer at home. Participants are asked to fill in the number of hours and minutes on an average day in the past seven days during the week and on an average day during the weekend. This is similar to the sitting measure of the International Physical Activity Questionnaire (IPAQ) which has shown to be valid and reliable (15). Sitting time was divided into the following 4 categories: (i) less than 4 hours a day, (ii) 4 to 8 hours a day, (iii) 8 to 11 hours a day and (iv) at least 11 hours a day or more (16). Prolonged sitting time was defined as sitting at least for 8 hours a day or more.

Participants are physically inactive if they (i) are sitting on average more than 8 hours a day, irrespective of the physical activity, or (ii) are sitting on average 4 hours or more a day and are less active than 150 minutes MVPA per week.

**Smoking**

Participants are asked three questions to measure smoking behaviour: (i) whether they have smoked in the past month, and (ii) whether they have smoked in the past, for at least one year (17). Smoking behaviour is categorized into non-smoker, past smoker and current smoker. Current smokers are defined as people who reported smoking in the past month. Past smokers reported smoking for at least one year, but did not smoke in the past month.

**Loneliness**

Loneliness is measured using De Jong Gierveld Loneliness Scale , which is a reliable and valid instrument to measure emotional, social and overall loneliness (18). Possible answers on this 6-item scale are: (i) yes!, (ii) yes, (iii) more or less, (iv), no, (v) no!. The overall loneliness score is calculated by counting the neutral and negative ("no!", "no", or "more or less") answers on items 4, 5 and 6 (social loneliness score) and by counting the positive ("more or less"", "yes" or "yes!") answers on items 1,2 and 3 (emotional loneliness score). Subsequently, the overall loneliness score is categorized into: (i) not lonely (0-1), (ii) moderate lonely (2-4), (iii) severe lonely (5-6). Loneliness is defined as an overall loneliness score of 2 or higher (18).

For peer review only

**Cardiovascular diseases**

Participants are asked whether they have suffered or still suffer from one of the following cardiovascular diseases: angina pectoris, myocardial infarction, transient ischemic attack (TIA), stroke or peripheral arterial diseases (yes/no). Presence of a cardiovascular disease is defined as having at least one of the above mentioned diseases.

**Hypertension**

Hypertension is determined based on the blood pressure measurement in which the systolic and diastolic blood pressure is measured both three times consecutively. The average of the second and the third measurement is used to determine the presence of hypertension. Hypertension is present: (i) if the systolic blood pressure is higher than 140 mmHg or diastolic blood pressure is higher than 90 mmHg (19), or (ii) if participants indicate that they receive medication (i.e. diuretics, beta blockers, ACE-inhibitors, angiotensin 2 antagonists and calcium antagonists) for their hypertension .

**High cholesterol**

High cholesterol is defined based on direct laboratory measurements using the fasting blood samples and self-reported questionnaires. High cholesterol is present if (i) the Low Density Lipoprotein (LDL) is higher than 2.5 mmol/l or (ii) the ratio of total cholesterol (TC) and High Density Lipoprotein (HDL) is higher than 5 mmol/l (19) or (iii) participants indicate that they receive medication (i.e. simvastinin, atorvastatin, rosuvastatin, pravastatin, ezetimib) to lower their cholesterol levels.

**Diabetes Mellitus**

The presence of diabetes mellitus (or impaired blood glucose levels) is based on direct laboratory measurements using the fasting blood samples and self-reported questionnaires. Diabetes Mellitus is defined as: (i) glucose (fasting capillary blood) of 7.0 mmol/l or higher, or (ii) glucose (fasting capillary blood) lower than 7.0 mmol/l accompanied by HbA1C levels higher than 53 mmol/mol (20).

For peer review only

HbA1C provides additional information on the average blood glucose levels during the previous month, while glucose may differ during the day (20).

**Obesity**

Body weight and body height are measured during physical examination in order to determine their Body Mass Index (BMI=kg/$m^2$)(21). Obesity is present if BMI is 30 kg/$m^2$ or higher (22).

**Renal dysfunction**

The presence of renal dysfunction is based on direct laboratory measurements (serum creatinine levels) using the fasting blood samples and self-reported questionnaires (23). Subsequently, the estimated glomerular filtration rate (eGFR) is calculated using the 2009 CKD-EPI creatinine equation (24,25) in order to determine participant's renal function (25). Renal dysfunction is present if (i) eGFR is lower than 60 ml/min/1.73 $m^2$ (26), or (ii) participants indicate that they receive medical treatment for the established renal dysfunction.

**Depression**

The level of depressive symptoms is measured using the Centre for Epidemiologic Studies Depression scale (CES-D). The CES-D consists of 20 items and is a reliable and valid tool to measure the current level of depressive symptoms in the general population (27). Answer options for each item are: rarely or none of the time (0), some or a little of the time (1), occasionally or a moderate amount of time (2), and most of all of the time (3). Total score varying from 0 to 60, indicates the level of depressive symptoms, a higher score reflects a higher level of depressive symptoms. Depression is defined as (i) having a score of 16 or higher (27), or (ii) participants indicate that they receive medical treatment for their depressive symptoms.

## REFERENCES


1. Morris MC, Tangney CC, Wang Y, Sacks FM, Barnes LL, Bennett DA, et al. MIND diet slows cognitive decline with aging. Alzheimer's Dement. 2015 Sep;11(9):1015–22.
2. Morris MC, Tangney CC, Wang Y, Sacks FM, Bennett DA, Aggarwal NT. MIND diet associated with reduced incidence of Alzheimer's disease. Alzheimer's Dement. 2015 Sep;11(9):1007–14.
3. Molag ML, de Vries JHM, Duif N, Ocké MC, Dagnelie PC, Goldbohm RA, et al. Selecting informative food items for compiling food-frequency questionnaires: comparison of procedures. Br J Nutr. 2010 Aug 8;104(03):446–56.
4. Siebelink E, Geelen A, de Vries JHM. Self-reported energy intake by FFQ compared with actual energy intake to maintain body weight in 516 adults. Br J Nutr. 2011 Jul 22;106(02):274–81.
5. Molag ML, de Vries JHM, Duif N, Ocké MC, Dagnelie PC, Goldbohm RA, et al. Selecting informative food items for compiling food-frequency questionnaires: comparison of procedures. Br J Nutr. 2010 Aug;104(03):446–56.
6. Anstey KJ, Mack HA, Cherbuin N. Alcohol Consumption as a Risk Factor for Dementia and Cognitive Decline: Meta-Analysis of Prospective Studies. Am J Geriatr Psychiatry. 2009 Jul;17(7):542–55.
7. Gezondheidsraad. Richtlijnen goede voeding 2015. Den Haag; 2015.
8. Wendel-Vos G, Schuit A. SQUASH: Short Questionnaire to Assess Health enhancing physical activity. Bilthoven, Netherlands; 2004.
9. Wagenmakers R, van den Akker-Scheek I, Groothoff JW, Zijlstra W, Bulstra SK, Kootstra JWJ, et al. Reliability and validity of the short questionnaire to assess health-enhancing physical activity (SQUASH) in patients after total hip arthroplasty. BMC Musculoskelet Disord. 2008 Jan 17;9(1):141.
10. de Hollander EL, Zwart L, de Vries SI, Wendel-Vos W. The SQUASH was a more valid tool than the OBiN for categorizing adults according to the Dutch physical activity and the combined guideline. J Clin Epidemiol. 2012 Jan;65(1):73–81.
11. Wendel-Vos G. Reproducibility and relative validity of the short questionnaire to assess health-enhancing physical activity. J Clin Epidemiol. 2003 Dec;56(12):1163–9.
12. Nicolaou M, Gademan MGJ, Snijder MB, Engelbert RHH, Dijkshoorn H, Terwee CB, et al. Validation of the SQUASH Physical Activity Questionnaire in a Multi-Ethnic Population: The HELIUS Study. Buchowski M, editor. PLoS One. 2016 Aug;11(8):e0161066.
13. Ainsworth BE, Haskell WL, Herrmann SD, Meckes N, Bassett DR, Tudor-Locke C, et al. 2011 Compendium of Physical Activities: a second update of codes and MET values. Med Sci Sports Exerc. 2011 Aug;43(8):1575–81.
14. Gezondheidsraad. Beweegrichtlijnen 2017. Den Haag; 2017.
15. CRAIG CL, MARSHALL AL, SJÖSTRÖM M, BAUMAN AE, BOOTH ML, AINSWORTH BE, et al. International Physical Activity Questionnaire: 12-Country Reliability and Validity. Med Sci Sport Exerc. 2003 Aug;35(8):1381–95.
16. van der Ploeg HP, Chey T, Korda RJ, Banks E, Bauman A. Sitting Time and All-Cause Mortality Risk in 222 497 Australian Adults. Arch Intern Med. 2012 Mar 26;172(6):494.
17. World Health Organization. Global Adult Tobacco Survey Collaborative Group. Tobacco Questions for Surveys: A Subset of Key Questions from the Global Adult Tobacco Survey (GATS) 2 nd Edition. 2011;6–9.
18. Gierveld JDJ, Tilburg T Van. A 6-Item Scale for Overall, Emotional, and Social Loneliness. Res Aging. 2006 Sep;28(5):582–98.
19. Nederlandse Huisartsen Genootschap. Praktische handleiding bij de NHG-Standaard CVRM (2019). 2019.
20. Rutten G, De Grauw W, Nijpels G, Houweling S, Van de Laar F, Bilo H, et al. NHG-Standaard Diabetes mellitus type 2. 2013.
21. World Health Organization (WHO). Body mass index - BMI [Internet]. World Health Organization; [cited 2019 May 6]. Available from: http://www.euro.who.int/en/health-topics/disease-prevention/nutrition/a-healthy-lifestyle/body-mass-index-bmi
22. Van Binsbergen J, Langens F, Dapper A, Van Halteren M, Glijsteen R, Cleyndert G, et


For peer review only

al. NHG-Standaard Obesitas | NHG. 2010.
23. Perrone RD, Madias NE, Levey AS. Serum creatinine as an index of renal function: new insights into old concepts. Clin Chem. 1992;38(10).
24. Levey AS, Stevens LA, Schmid CH, Zhang YL, Castro AF, Feldman HI, et al. A new equation to estimate glomerular filtration rate. Ann Intern Med. 2009;150(9):604–12.
25. Levey AS, Inker LA, Coresh J. GFR estimation: From physiology to public health. Am J Kidney Dis. 2014;63(5):820–34.
26. De Grauw W, De Leest K, Schenk P, Scherpbier-De Haan N, Tjin-A-Ton J, Tuut M, et al. NHG-Standaard Chronische Nierschade. TPO - Prakt. 2018;13(5):26–9.
27. Smarr KL, Keefer AL. Measures of depression and depressive symptoms: Beck Depression Inventory-II (BDI-II), Center for Epidemiologic Studies Depression Scale (CES-D), Geriatric Depression Scale (GDS), Hospital Anxiety and Depression Scale (HADS), and Patient Health Questionna. Arthritis Care Res (Hoboken). 2011 Nov;63(S11):S454–66.

For peer review only

**Supplementary file 2. Overview of assessment measures at baseline and follow up**

**Table 1.** Assessment measures at baseline and follow up

| | **Baseline** | **3 months** | **6 months** | **9 months** | **12months** |
|---|---|---|---|---|---|
| **GENERAL INFORMATION** | | | | | |
| Age, gender, ethnicity, education and postal code | Q | | | | |
| Participation in the Lifelines cohort | Q | | | | |
| Medical family history | Q | | | | |
| Health literacy (S-TOFHLA, 3-items) | Q | | | | |
| **RISK AND PROTECTIVE FACTORS** | | | | | |
| Smoking | Q | Q | Q | Q | Q |
| Physical inactivity (SQUASH, IPAQ sitting measure) | Q | Q | Q | Q | Q |
| Mediterranean diet (FFQ) | Q | Q | Q | Q | Q |
| Alcohol consumption (FFQ) | Q | Q | Q | Q | Q |
| High cognitive activity (CRIq adapted) | Q | Q | Q | Q | Q |
| Loneliness (de Jong Gierveld, 6-items) | Q | Q | Q | Q | Q |
| Cardiovascular diseases (CVD) | Q | Q | Q | Q | Q |
| Obesity (body weight, height) | Q+ PE | Q | Q | Q | Q+PE |
| Hypertension (SBD, DBP) | Q+PE | Q | Q | Q | Q+PE |
| High cholesterol (LDL, HDL, TC) | Q+BS | Q | Q | Q | Q+BS |
| Diabetes Mellitus[1] (glucose, | Q+BS | Q | Q | Q | Q+BS |

For peer review only

| | | | | | |
|---|---|---|---|---|---|
| HbA1C) | | | | | |
| Renal dysfunction (eGFR) | Q+BS | Q | Q | Q | Q+BS |
| Depression (CES-D) | Q | Q | Q | Q | Q |
| **OTHER PARAMETERS** | | | | | |
| Medical treatment of disease | Q | Q | Q | Q | Q |
| Motivation to change lifestyle (MCLHB-DRR) | Q | Q | Q | Q | Q |
| Stages of change | Q | Q | Q | Q | Q |
| Hearing problems | Q | Q | Q | Q | Q |
| Subjective stress (LDI) | Q | | | | Q |
| Memory complaints | Q | | | | |
| Quality of life (2 items of SF36, VAS-score) | Q | | | | Q |
| Perceived living environment | Q | | | | Q |
| Compliance lifestyle advice per individual health behaviour | | Q | Q | Q | Q |
| Compliance advice contact with GP | | Q | Q | Q | Q |

*SQUASH* Short Questionnaire to Assess Health-enhancing physical activity, *IPAQ* International Physical Activity Questionnaire, *FFQ* Food Frequency Questionnaire, *CRIq* Cognitive Reserve Index questionnaire, *CVD* Cardiovascular diseases, *SBP* Systolic Blood Pressure, *DBP* Diastolic Blood Pressure, *HDL* high-density lipoproteins, *LDL* low-density lipoproteins, *TC* total cholesterol, *HbA1C* Hemoglobin A1C, *eGFR* estimated Glomerular Filtration Rate, *CES-D* Centre for Epidemiological Studies Depression Scale, *MCLHB-DRR* Motivation to Change Lifestyle and Health Behavior for Dementia Risk Reduction Scale, LDI Long-term Difficulties Inventory, *SF36* Short Form 36 items, *VAS* Visual Analogue Scale

Q: Online questionnaire; PE: Physical examination; BS: Blood sample

For peer review only

Voornaam Achternaam
Straatnaam 12
1234AB Plaatsnaam
0612345678
demin@umcg.nl
ABCDE
Aangemeld op: 01-02-2020 10:01:16

01-02-2020 10:06:24

Beste Voornaam Achternaam,

Indien u wilt deelnemen aan dit onderzoek, vragen wij u dit toestemmingformulier door middel van een elektronische handtekening te ondertekenen. Hieronder vindt u de voorwaarde voor deelname aan dit onderzoek.

**Wanneer u het toestemmingsformulier ondertekent verklaart u dat:**
- u de informatiefolder met bijlagen en bovenstaande informatie heeft gelezen en hiermee voldoende bent geïnformeerd over het doel en de uitvoering van het onderzoek.
- U de mogelijkheid heeft gehad om aanvullende vragen te stellen (telefonisch of per mail), welke naar tevredenheid zijn beantwoord.
- u genoeg tijd had om te beslissen of u wilt deelnemen.
- u weet dat deelname vrijwillig is en dat u op ieder moment kan beslissen om toch niet mee te doen of te stoppen met het onderzoek.
- u weet dat u op de hoogte gesteld kan worden van medische relevante bevindingen.

**U geeft toestemming:**
- voor deelname aan het landelijk proef-bevolkingsonderzoek naar de beschermende en risicofactoren voor dementie (Demin studie).
- dat u in de toekomst opnieuw benaderd kan worden voor deelname aan aanvullend onderzoek.
- om uw onderzoeksgegevens te koppelen aan gegevens van het Centraal Bureau voor Statistiek (CBS), zoals uw gegevens over woonomgeving (bijvoorbeeld sportfaciliteiten).

Met vriendelijke groet,

Het Demin team
**www.demin.nl**

---
Ik verklaar dat ik deze proefpersoon volledig heb geïnformeerd over het genoemde onderzoek.

Als er tijdens het onderzoek informatie bekend wordt die de toestemming van de proefpersoon zou kunnen beïnvloeden, dan breng ik hem/haar daarvan tijdig op de hoogte.

Naam hoofdonderzoeker:
Handtekening: Datum:
Tijdstip:

*De deelnemer krijgt een volledige informatiebrief, samen met een kopie van het getekende toestemmingsformulier.*

For peer review only

# Reporting checklist for protocol of a clinical trial.

Based on the SPIRIT guidelines.

## Instructions to authors

Complete this checklist by entering the page numbers from your manuscript where readers will find each of the items listed below.

Your article may not currently address all the items on the checklist. Please modify your text to include the missing information. If you are certain that an item does not apply, please write "n/a" and provide a short explanation.

Upload your completed checklist as an extra file when you submit to a journal.

In your methods section, say that you used the SPIRITreporting guidelines, and cite them as:

Chan A-W, Tetzlaff JM, Altman DG, Laupacis A, Gøtzsche PC, Krleža-JeriĆ K, Hróbjartsson A, Mann H, Dickersin K, Berlin J, Doré C, Parulekar W, Summerskill W, Groves T, Schulz K, Sox H, Rockhold FW, Rennie D, Moher D. SPIRIT 2013 Statement: Defining standard protocol items for clinical trials. Ann Intern Med. 2013;158(3):200-207

| | | Reporting Item | Page Number |
|---|---|---|---|
| **Administrative information** | | | |
| Title | #1 | Descriptive title identifying the study design, population, interventions, and, if applicable, trial acronym | 1 |
| Trial registration | #2a | Trial identifier and registry name. If not yet registered, name of intended registry | 3 |
| Trial registration: data set | #2b | All items from the World Health Organization Trial Registration Data Set | All pages, no results |
| Protocol version | #3 | Date and version identifier | 1 |
| Funding | #4 | Sources and types of financial, material, and other support | 21 |
| Roles and responsibilities: contributorship | #5a | Names, affiliations, and roles of protocol contributors | 21 |
| Roles and responsibilities: sponsor contact information | #5b | Name and contact information for the trial sponsor | 1 |

For peer review only

| | | | |
|---|---|---|---|
| Roles and responsibilities: sponsor and funder | #5c | Role of study sponsor and funders, if any, in study design; collection, management, analysis, and interpretation of data; writing of the report; and the decision to submit the report for publication, including whether they will have ultimate authority over any of these activities | 23 |
| Roles and responsibilities: committees | #5d | Composition, roles, and responsibilities of the coordinating centre, steering committee, endpoint adjudication committee, data management team, and other individuals or groups overseeing the trial, if applicable (see Item 21a for data monitoring committee) | 23 |
| **Introduction** | | | |
| Background and rationale | #6a | Description of research question and justification for undertaking the trial, including summary of relevant studies (published and unpublished) examining benefits and harms for each intervention | 5-6 |
| Background and rationale: choice of comparators | #6b | Explanation for choice of comparators | 6 |
| Objectives | #7 | Specific objectives or hypotheses | 7 |
| Trial design | #8 | Description of trial design including type of trial (eg, parallel group, crossover, factorial, single group), allocation ratio, and framework (eg, superiority, equivalence, non-inferiority, exploratory) | 7-8 |
| **Methods: Participants, interventions, and outcomes** | | | |
| Study setting | #9 | Description of study settings (eg, community clinic, academic hospital) and list of countries where data will be collected. Reference to where list of study sites can be obtained | 7 |
| Eligibility criteria | #10 | Inclusion and exclusion criteria for participants. If applicable, eligibility criteria for study centres and individuals who will perform the interventions (eg, surgeons, psychotherapists) | 8-9 |
| Interventions: description | #11a | Interventions for each group with sufficient detail to allow replication, including how and when they will be administered | 9-16 |
| Interventions: modifications | #11b | Criteria for discontinuing or modifying allocated interventions for a given trial participant (eg, drug dose change in response to harms, participant request, or | n/a |

For peer review only

| | | | |
|---|---|---|---|
| | | improving / worsening disease) | |
| Interventions: adherance | #11c | Strategies to improve adherence to intervention protocols, and any procedures for monitoring adherence (eg, drug tablet return; laboratory tests) | 10 |
| Interventions: concomitant care | #11d | Relevant concomitant care and interventions that are permitted or prohibited during the trial | |
| Outcomes | #12 | Primary, secondary, and other outcomes, including the specific measurement variable (eg, systolic blood pressure), analysis metric (eg, change from baseline, final value, time to event), method of aggregation (eg, median, proportion), and time point for each outcome. Explanation of the clinical relevance of chosen efficacy and harm outcomes is strongly recommended | 16-20 |
| Participant timeline | #13 | Time schedule of enrolment, interventions (including any run-ins and washouts), assessments, and visits for participants. A schematic diagram is highly recommended (see Figure) | 11, 16 |
| Sample size | #14 | Estimated number of participants needed to achieve study objectives and how it was determined, including clinical and statistical assumptions supporting any sample size calculations | 9 |
| Recruitment | #15 | Strategies for achieving adequate participant enrolment to reach target sample size | 7 |
| **Methods: Assignment of interventions (for controlled trials)** | | | |
| Allocation: sequence generation | #16a | Method of generating the allocation sequence (eg, computer-generated random numbers), and list of any factors for stratification. To reduce predictability of a random sequence, details of any planned restriction (eg, blocking) should be provided in a separate document that is unavailable to those who enrol participants or assign interventions | 8-9 |
| Allocation concealment mechanism | #16b | Mechanism of implementing the allocation sequence (eg, central telephone; sequentially numbered, opaque, sealed envelopes), describing any steps to conceal the sequence until interventions are assigned | n/a |
| Allocation: implementation | #16c | Who will generate the allocation sequence, who will enrol participants, and who will assign participants to interventions | 8-9 |
| Blinding (masking) | #17a | Who will be blinded after assignment to interventions (eg, trial participants, care providers, outcome assessors, data analysts), and how | n/a, one recruitment strategy per |

For peer review only

| | | | |
|---|---|---|---|
| | | | memory clinic |
| Blinding (masking): emergency unblinding | #17b | If blinded, circumstances under which unblinding is permissible, and procedure for revealing a participant's allocated intervention during the trial | n/a |
| **Methods: Data collection, management, and analysis** | | | |
| Data collection plan | #18a | Plans for assessment and collection of outcome, baseline, and other trial data, including any related processes to promote data quality (eg, duplicate measurements, training of assessors) and a description of study instruments (eg, questionnaires, laboratory tests) along with their reliability and validity, if known. Reference to where data collection forms can be found, if not in the protocol | 11 |
| Data collection plan: retention | #18b | Plans to promote participant retention and complete follow-up, including list of any outcome data to be collected for participants who discontinue or deviate from intervention protocols | 10 |
| Data management | #19 | Plans for data entry, coding, security, and storage, including any related processes to promote data quality (eg, double data entry; range checks for data values). Reference to where details of data management procedures can be found, if not in the protocol | 10-11 |
| Statistics: outcomes | #20a | Statistical methods for analysing primary and secondary outcomes. Reference to where other details of the statistical analysis plan can be found, if not in the protocol | 19 |
| Statistics: additional analyses | #20b | Methods for any additional analyses (eg, subgroup and adjusted analyses) | n/a |
| Statistics: analysis population and missing data | #20c | Definition of analysis population relating to protocol non-adherence (eg, as randomised analysis), and any statistical methods to handle missing data (eg, multiple imputation) | n/a |
| **Methods: Monitoring** | | | |
| Data monitoring: formal committee | #21a | Composition of data monitoring committee (DMC); summary of its role and reporting structure; statement of whether it is independent from the sponsor and competing interests; and reference to where further details about its charter can be found, if not in the protocol. Alternatively, an explanation of why a DMC is not needed | 22 |

For peer review only

| | | | |
|---|---|---|---|
| Data monitoring: interim analysis | #21b | Description of any interim analyses and stopping guidelines, including who will have access to these interim results and make the final decision to terminate the trial | n/a, low risk |
| Harms | #22 | Plans for collecting, assessing, reporting, and managing solicited and spontaneously reported adverse events and other unintended effects of trial interventions or trial conduct | 20-21 |
| Auditing | #23 | Frequency and procedures for auditing trial conduct, if any, and whether the process will be independent from investigators and the sponsor | 16-17 |
| **Ethics and dissemination** | | | |
| Research ethics approval | #24 | Plans for seeking research ethics committee / institutional review board (REC / IRB) approval | 22 |
| Protocol amendments | #25 | Plans for communicating important protocol modifications (eg, changes to eligibility criteria, outcomes, analyses) to relevant parties (eg, investigators, REC / IRBs, trial participants, trial registries, journals, regulators) | n/a |
| Consent or assent | #26a | Who will obtain informed consent or assent from potential trial participants or authorised surrogates, and how (see Item 32) | 22 |
| Consent or assent: ancillary studies | #26b | Additional consent provisions for collection and use of participant data and biological specimens in ancillary studies, if applicable | n/a |
| Confidentiality | #27 | How personal information about potential and enrolled participants will be collected, shared, and maintained in order to protect confidentiality before, during, and after the trial | 21 |
| Declaration of interests | #28 | Financial and other competing interests for principal investigators for the overall trial and each study site | 22 |
| Data access | #29 | Statement of who will have access to the final trial dataset, and disclosure of contractual agreements that limit such access for investigators | 16, 22 |
| Ancillary and post trial care | #30 | Provisions, if any, for ancillary and post-trial care, and for compensation to those who suffer harm from trial participation | n/a, low risk |
| Dissemination policy: trial results | #31a | Plans for investigators and sponsor to communicate trial results to participants, healthcare professionals, the public, and other relevant groups (eg, via publication, reporting in results databases, or other data sharing arrangements), including any publication restrictions | 21 |

For peer review only

| | | | |
|---|---|---|---|
| Dissemination policy: authorship | #31b | Authorship eligibility guidelines and any intended use of professional writers | 21 |
| Dissemination policy: reproducible research | #31c | Plans, if any, for granting public access to the full protocol, participant-level dataset, and statistical code | 21 |
| **Appendices** | | | |
| Informed consent materials | #32 | Model consent form and other related documentation given to participants and authorised surrogates | 21 |
| Biological specimens | #33 | Plans for collection, laboratory evaluation, and storage of biological specimens for genetic or molecular analysis in the current trial and for future use in ancillary studies, if applicable | n/a |

None The SPIRIT checklist is distributed under the terms of the Creative Commons Attribution License CC-BY-ND 3.0. This checklist can be completed online using https://www.goodreports.org/, a tool made by the EQUATOR Network in collaboration with Penelope.ai

For peer review only
